# Amino‐Supported Palladium Catalyst for Chemo‐ and Stereoselective Domino Reactions

**DOI:** 10.1002/anie.202011708

**Published:** 2020-11-10

**Authors:** Man‐Bo Li, Jie Yang, Ying Yang, Guo‐Yong Xu, Gen Luo, Jianping Yang, Jan‐E. Bäckvall

**Affiliations:** ^1^ Institute of Physical Science and Information Technology Anhui University Hefei Anhui 230601 P. R. China; ^2^ Department of Organic Chemistry Arrhenius Laboratory Stockholm University SE-10691 Stockholm Sweden; ^3^ Department of Natural Sciences Mid Sweden University SE-85170 Sundsvall Sweden; ^4^ School of Materials Science and Engineering Jiangsu University of Science and Technology Zhenjiang 212003 P. R. China

**Keywords:** amines, cyclizations, heterogeneous catalysis, palladium, supported catalysts

## Abstract

A solid amino‐supported palladium catalyst is used in an oxidative domino reaction for the diastereoselective construction of alkyne‐substituted cyclopentenol compounds. This heterogeneous catalyst exhibits high efficiency and excellent chemoselectivity, as well as good recyclability. The chemoselectivity of the domino reactions was readily controlled by switching the solvent and catalyst. Asymmetric syntheses and an oxidative carbocyclization‐borylation reaction have also been developed based on the heterogeneous palladium catalyst.

## Graphical abstract

An oxidative domino reaction is developed here for the construction of alkyne‐substituted cyclopentenol compounds by using an amino‐supported Pd catalyst. The properties of the heterogeneous catalyst contribute to its high efficiency and excellent chemoselectivity, as well as good recyclability.

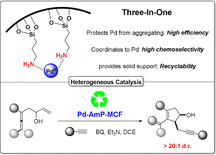

A domino reaction constitutes an efficient approach in organic synthesis involving multiple bond formation, as it produces the target molecule in one pot with high atom and step economy.[^1^, ^2^, ^3^, ^4^, ^5^, ^6^] However, the achievement of high selectivity in a domino reaction is always challenging, considering the possible side reactions during each bond‐forming step.[^3^, ^4^, ^5^, ^6^] During recent years, our group has been involved in Pd‐catalyzed oxidative domino processes for the construction of complex molecules from enallenes (Scheme 1 a).[^7^, ^8^, ^9^, ^10^, ^11^, ^12^] The key feature of these processes is the generation of a vinyl‐palladium intermediate (***Int*** **‐B**) triggered by an allene attack on palladium via a C(sp^3^)−H bond cleavage (***Int*** **‐A**).^[7]^ The diverse reactivity of ***Int*** **‐B** allows flexible domino processes, while at the same time significant selectivity challenges are involved in these reactions.

**Scheme 1:**
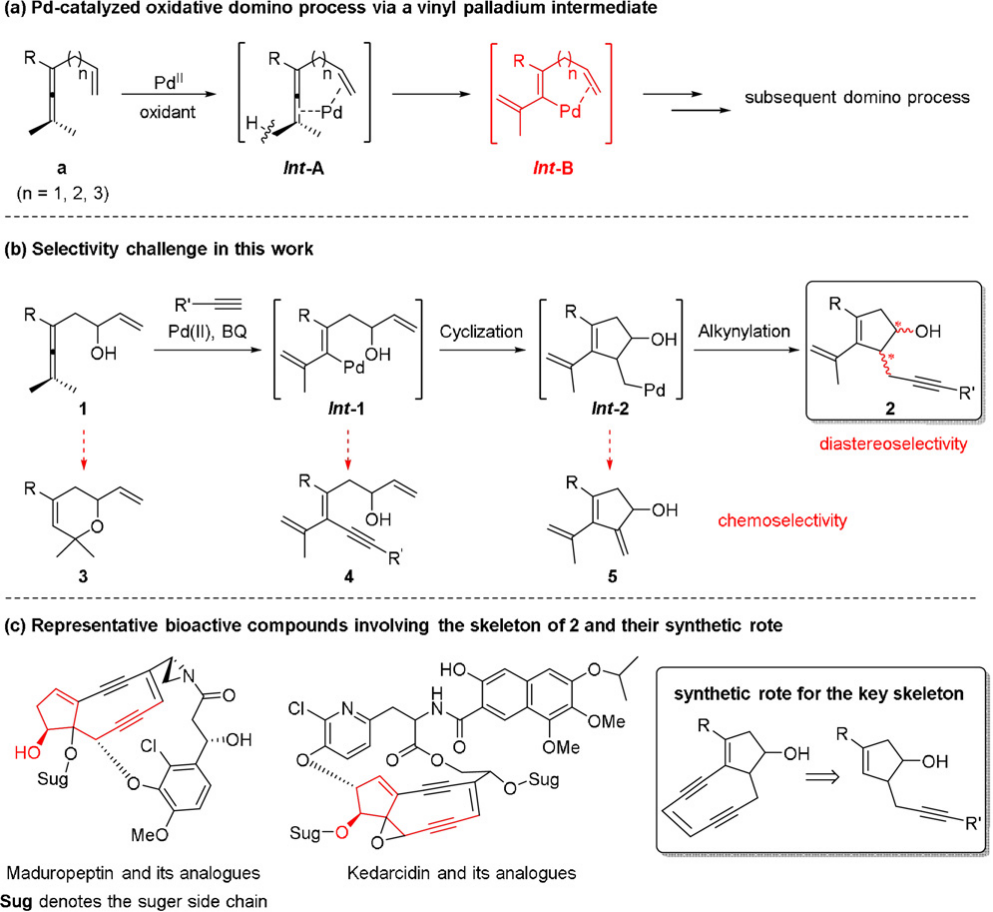
Proposed domino process, selectivity challenge, and representative bioactive compounds.

In the present work, we have designed a Pd‐catalyzed oxidative domino route to enynes **2** with a cyclopentenol unit (Scheme 1 b). This skeleton is a key substructure[^13^, ^14^, ^15^, ^16^, ^17^, ^18^] and synthon^[19]^ of many bioactive compounds. Representative examples include maduropeptin,[^14^, ^15^, ^18^] kedarcidin[^13^, ^16^, ^17^] and their analogues (Scheme 1 c). In vivo studies have shown these molecules to be extremely active against leukemia and melanoma by binding and cleaving duplex DNA at selected sites.[^13^, ^14^, ^15^] Concerning the construction of this important skeleton, the synthetic route in Scheme 1 b would be a highly efficient approach. However, considering the possible side reactions of each palladium intermediate and the newly formed chiral centers during the domino process, chemo‐ and stereoselective formation of **2** would be challenging.

Our initial attempt began with the reaction of **1 a** and phenylacetylene in the presence of 5 mol % of Pd(OAc)_2_ and 1.1 equiv of benzoquinone (BQ) in DCE at room temperature (Table 1). To our delight, the target product **2 a** was obtained in 10 % yield as the *cis*‐diasteromer with high diastereoselectivity (>20:1 d.r.). However, the chemoselectivity was not satisfactory, and **5 a** generated from β‐H elimination of ***Int*** **‐2** was obtained as the major product in 69 % yield (entry 1). To improve the chemoselectivity of the domino reaction for producing **2 a**, attempts were made to optimize the reaction conditions. Replacement of Pd(OAc)_2_ by other homogeneous palladium sources did not give any better results, and by using Pd(PPh_3_)_2_Cl_2_, only non‐oxidized product **3 a** was obtained, which is generated from Pd‐catalyzed intramolecular oxypalladation[^20^, ^21^] (entry 3). Compound **5 a** was the dominant product in most of the solvents (entries 4–8), and was obtained as the sole product in 90 % yield in CHCl_3_. Intriguingly, by using CH_3_CN as the solvent, **4 a** was obtained as the major product in 85 % yield (entry 8). This result could be explained by the interaction between CH_3_CN and Pd^II^, which inhibits the Pd‐catalyzed olefin insertion step.^[22]^ Under the homogeneous reaction conditions, **2 a** was always the minor product. We then turned our attention to a solid catalyst where palladium is immobilized on amino‐functionalized siliceous mesocellular foam[^23^, ^24^] (Pd‐AmP‐MCF). By using this catalyst, our group has successfully realized the oxidative transformations of allenes with high activity and selectivity.[^25^, ^26^, ^27^, ^28^] Surprisingly, highly improved selectivity for **2 a** was observed when the catalyst was switched to Pd‐AmP‐MCF, and **2 a** was now obtained in 44 % yield (entry 9). In our previous work the addition of catalytic amount of AgOTf dramatically improved the activity of this heterogeneous palladium catalyst in oxidative carbonylation of allene amides.^[28]^ However, addition of AgOTf in the present reaction resulted in the sole production of **3 a** (entry 10).^[29]^ Finally, after screening other additives, we were delighted to find that addition of Et_3_N is favorable for switching the selectivity of the reaction towards **2 a**. Thus, the use of 0.1 equiv of Et_3_N affored **2 a** in 84 % yield (entry 11). Notably, the solid palladium catalyst loading can be reduced to 1 mol %, producing **2 a** in 83 % yield (entry 12). A similar solid palladium catalyst immobilized on renewable amino‐functionalized crystalline nanocllulose foam^[28]^ (Pd‐AmP‐CNC) also showed high activity and selectivity for the formation of **2 a** (entry 13).

**Table 1:** Optimization of the reaction conditions.^[a]^ 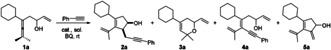

Entry	Cat. (5 mol %)	Sol.	Yield [%]^[b]^			
			**2 a**	**3 a**	**4 a**	**5 a**
1	Pd(OAc)_2_	DCE	10	–	12	69
2	Pd(TFA)_2_	DCE	–	22	10	60
3	Pd(PPh_3_)_2_Cl_2_	DCE	–	89	–	–
4	Pd(OAc)_2_	THF	5	–	20	71
5	Pd(OAc)_2_	Toluene	–	–	20	74
6	Pd(OAc)_2_	CHCl_3_	–	–	–	90
7	Pd(OAc)_2_	Acetone	–	10	25	54
8	Pd(OAc)_2_	CH_3_CN	–	–	85	3
9	Pd‐AmP‐MCF	DCE	44	–	5	35
10^[c]^	Pd‐AmP‐MCF	DCE	–	94	–	–
11^[d]^	Pd‐AmP‐MCF	DCE	84	–	–	4
12^[d,e]^	Pd‐AmP‐MCF	DCE	83	–	–	4
13^[d,e]^	Pd‐AmP‐CNC	DCE	80	–	–	6

[a] Unless otherwise noted, the reaction was carried out by using 0.2 mmol of **1 a**, 0.25 mmol of phenylacetylene, 5 mol % of catalyst, 1.1 equiv of BQ and 1.0 mL of solvent at room temperature for 12 h. [b] Determined by NMR spectroscopy using anisole as the internal standard. [c] 5 mol % of AgOTf was added. [d] 0.1 equiv of Et_3_N was added. [e] 1 mol % of catalyst was used, 8 h.

For in‐depth understanding of the origin of the high activity and chemoselectivity in the heterogeneous palladium‐catalyzed domino process for the formation of **2**, control experiments were conducted by using 1 mol % of Pd‐AmP‐MCF or Pd(OAc)_2_ with different amine additives (Table 2).^[30]^ Some interesting results were observed and the following conclusions were made: 1) Under both heterogeneous and homogeneous reaction conditions, tertiary, secondary, and primary amines improved the selectivity for **2 a**,^[31]^ and tertiary amines dramatically increased the yield of **2 a** in the reaction. Diamine completely shut down the reaction, probably due to the strong coordination of diamine to Pd^II^, which inhibits the catalytic activity of the solid palladium catalyst. These results indicate that amine coordinates to Pd^II^ and affects its catalytic activity and selectivity,[^32^, ^33^, ^34^] and tertiary, secondary and primary amines are positive for improving the selectivity for **2 a** by suppressing the β‐H elimination or promoting the alkyne ligand exchange of ***Int*** **‐2**.[^35^, ^36^] 2) Under homogeneous reaction conditions, the starting material was always partially recovered and considerable amounts of Pd black was observed after the completion of the reaction (Figure 1 a and b, inset). XPS Pd3d analysis of the reaction mixture showed that the proportion of Pd^II^ was much lower than that of Pd^0^ (Figure 1 a and b). In contrast, under the heterogeneous reaction conditions, Pd^II^ was still dominant after the reaction (Figure 1 c and d).^[37]^ These results demonstrate that the porous amino support (AmP‐MCF) protects Pd species from aggregating to Pd black, and in this way the activity of Pd‐AmP‐MCF was maintained during the catalytic cycle. The partial recovery of starting material under the homogeneous reaction conditions can be explained by the deactivation of active Pd^II^ to Pd black. As a result, Pd‐AmP‐MCF exhibited much higher activity and chemoselectivity than the homogeneous Pd catalyst, which is credited to the interaction between its porous amino support and Pd^II^ (Figure 1 e).

**Figure 1:**
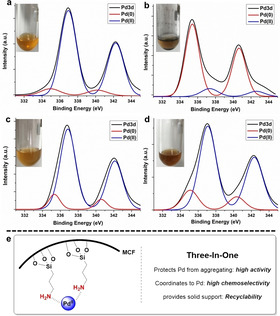
XPS Pd3d spectra of the reaction mixture by using Pd(OAc)_2_ (a: before reaction; b: after reaction) or Pd‐AmP‐MCF (c: before reaction; d: after reaction) as the catalyst. Inset: Photos of the reaction mixture. e) An illustration of the heterogeneous catalyst and its three‐in‐one role.

**Table 2:** Amine effect under homogeneous and heterogeneous conditions.

	Catalyst and Yield [%]			
Amine (0.1 equiv)	Pd‐AmP‐MCF		Pd(OAc)_2_	
	**2 a**	**5 a**	**2 a**	**5 a**
none	44	35	5^[b]^	35^[b]^
Pr_3_N	82	4	46^[b]^	0^[b]^
Pr_2_NH	47	38	28^[b]^	14^[b]^
PrNH_2_	45	40	25^[b]^	15^[b]^
TMEDA	0^[a]^	0^[a]^	0^[a]^	0^[a]^

Reaction conditions: **1 a** (0.2 mmol), phenylacetylene (0.25 mmol), Pd catalyst (1 mol %), amine additive (0.1 equiv), BQ (1.1 equiv), DCE (1.0 mL), 8 h. [a] **1 a** was recovered in >90 % yield. [b] **1 a** was partially recovered.

With the optimized reaction conditions in hand, we investigated the substrate scope (Scheme 2). Aromatic and heteroaromatic terminal alkynes all worked well with enallene **1** to give **2** in high yields (**2 a**–**l**). Arylalkynes with electron‐withdrawing groups or electron‐donating groups at *para*‐, *ortho*‐, or *meta*‐positions worked equally well with enallene **1**, affording **2** in good yields (**2 b**–**j**). Functional groups such as MeO, O_2_N, F, Cl, Br, F_3_C and MeO_2_C were well tolerated under the standard reaction conditions. Aliphatic terminal alkynes reacted with enallene **1**, giving **5** as the major products, probably due to the unfavorable alkynylation between aliphatic alkynes and ***Int*** **‐2** (Scheme 1 b). Different substituents including cyclohexyl (**2 a**), cyclopentyl (**2 p**), cyclopropyl (**2 o**), propyl (**2 m**), butyl (**2 n**) and phenylethyl (**2 q**) at the R position of enallenes **1** worked well to give products **2** in >80 % yields.^[38]^ Interestingly, the addition of Et_3_N was not necessary when a tertiary amine was introduced in the substrate (**2 r**). Notably, Pd‐AmP‐MCF catalyzed the oxidative domino process with high efficiency and diastereoselectivity. For all of the substrates, only 1 mol % of Pd‐AmP‐MCF was used and products **2** were obtained in >20:1 d.r.

**Scheme 2:**
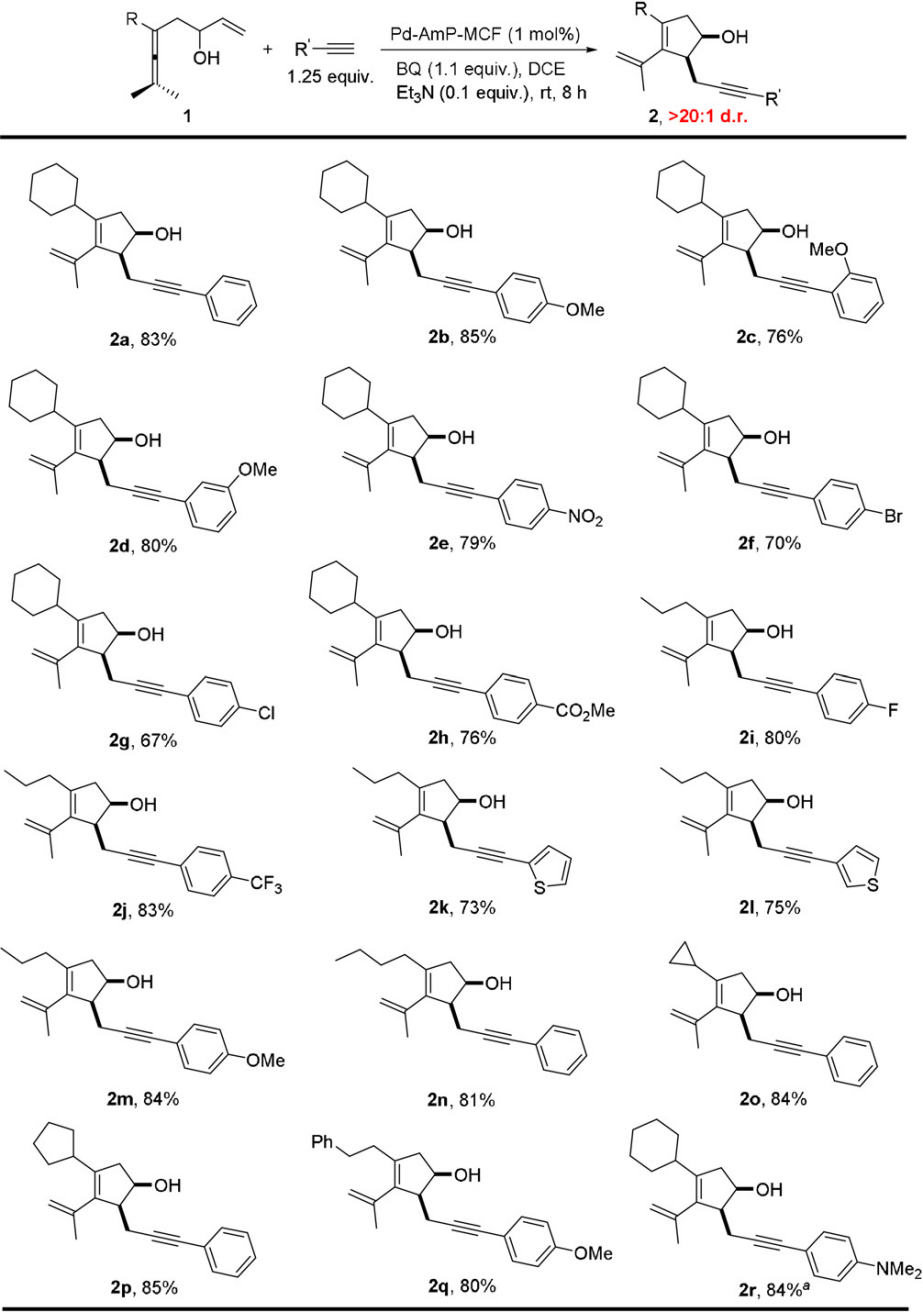
Substrate scope for the synthesis of **2**. [a] Without Et_3_N.

Inductively coupled plasma optical emisson spectroscopy (ICP‐OES) analysis after the reaction indicates that there was no detectable amount of Pd in the reaction solution (<0.1 ppm). A hot filtration test^[39]^ showed that no active Pd species were leached out from the solid catalyst during the reaction. These results rule out a Pd leaching during or after reaction, suggesting a heterogeneous pathway. Recycling experiments and kinetic studies (Figure 2) reveal that the solid Pd catalyst is recoverable, and its activity is essentially maintained between the first and the seventh cycles, which demonstrates that Pd‐AmP‐MCF is robust and highly active for the Pd‐catalyzed oxidative domino process. However, we cannot exclude the possibility that the solid catalyst might lose its activity slowly after the long run of the reaction.^[40]^

**Figure 2:**
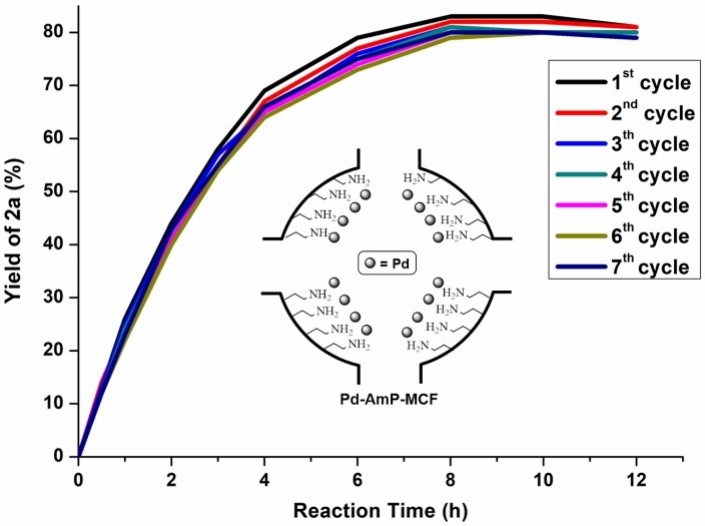
Rrecycling experiments and kinetic studies.

By readily switching the reaction conditions, using AgOTf or Pd(OAc)_2_ (5 mol %) as the catalyst, DCE, CH_3_CN or CHCl_3_ as the solvent, the chemoselectivity of the reaction is simply controlled to give **3**, **4** or **5** as the final products in excellent yields (Scheme 3). Notably, Pd‐AmP‐MCF showed much higher efficiency than Pd(OAc)_2_, and 1 mol % of Pd(OAc)_2_ resulted in partially recovery of the starting materials. The solvent effect was also extended to the solid catalyst in CH_3_CN, however, in CHCl_3_, Pd‐AmP‐MCF catalyzed the reaction, giving a mixture of **2** and **5** as the products.^[41]^ This catalyst/solvent‐controlled selective domino process provides an efficient pathway towards the chemodivergent synthesis of **2, 3**, **4** and **5**.

**Scheme 3:**
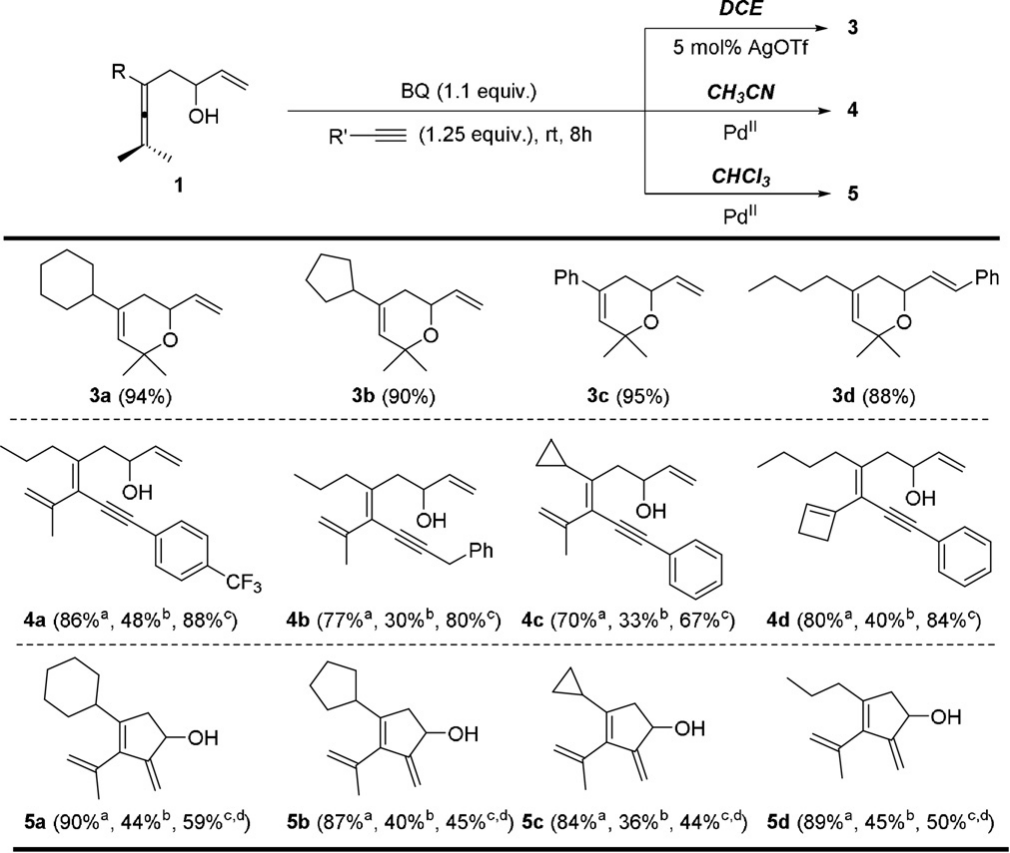
Chemodivergent syntheses of **3**, **4** and **5**. [a] 5 mol % of Pd(OAc)_2_. [b] 1 mol % of Pd(OAc)_2_, starting materials were partially recovered. [c] 1 mol % of Pd‐AmP‐MCF. [d]>90 % conversion, **2** was obtained as the byproducts.

Enantiomerically pure enallene (*R*)‐**1**, readily obtained from kinetic resolution of **1** with *Candida Antarctica* lipase B (CalB),^[42]^ was selectively transformed to optically pure (>99 % *ee*) products **2**, **4** or **5** in high yields by simply adjusting the solvent or catalytic system of the reaction (Scheme 4 a). During the domino process, no loss of optical purity was observed, despite the possible racemization pathway of the allylic alcohol moiety of allene (*R*)‐**1** in the presence of palladium. Under the standard heterogeneous reaction conditions, by replacing the alkyne reaction partner with bis(pinacolato)diboron (B_2_pin_2_), an oxidative carbocyclization‐borylation domino process was developed to give cyclopentenol boron compound **6** in high yield and high diastereoselectivity (Scheme 4 b).

**Scheme 4:**
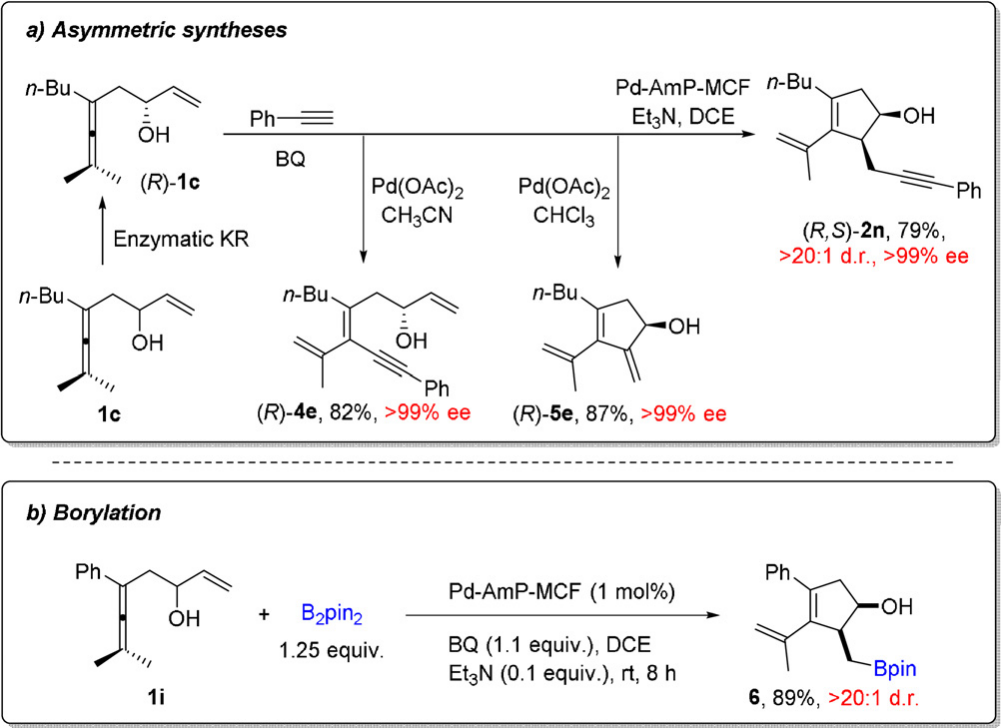
Applications of the solid palladium catalyzed domino process.

Based on the experimental results and our previous work on Pd‐catalyzed oxidative transformations of allene derivatives,[^7^, ^8^, ^9^, ^10^, ^11^, ^12^, ^25^, ^26^, ^27^, ^28^] we propose the mechanism shown in Scheme 5 for the Pd‐catalyzed domino reactions. Initially, simultaneous coordination of the olefin group and the allene unit of enallene **1** to Pd^II^ would trigger the allene attack to generate ***Int*** **‐1**, which would react with terminal alkyne to give product **4** in CH_3_CN, or undergo a face‐selective olefin insertion to generate ***Int*** **‐2** with hydroxy group and Pd on the same side.^[11]^ Under homogeneous reaction conditions (Pd(OAc)_2_, CHCl_3_), a subsequent β‐H elimination of ***Int*** **‐2** would produce **5**, while under heterogeneous reaction conditions (Pd‐AmP‐MCF, Et_3_N, DCE), ***Int*** **‐2** prefers to react with alkyne producing **2** via ***Int*** **‐3**. The Pd^0^ would be reoxidized by BQ to generate active Pd^II^ to initiate the next catalytic cycle.

**Scheme 5:**
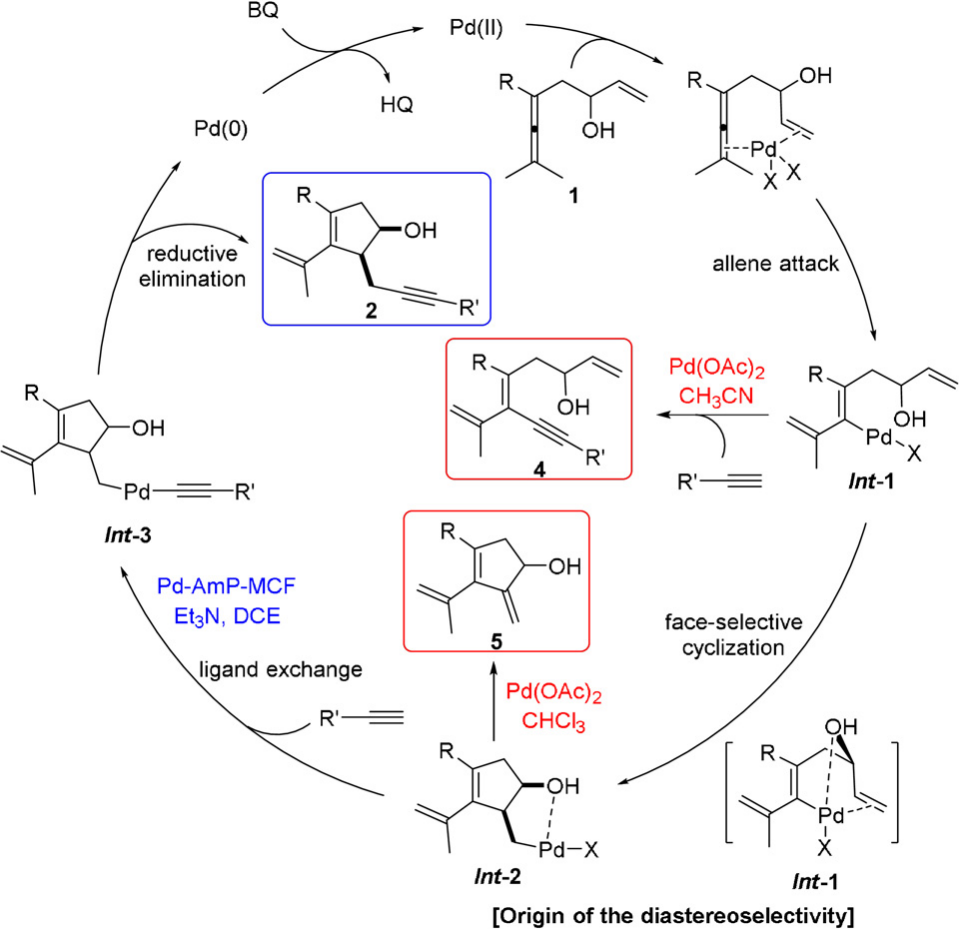
Proposed mechanism.

In conclusion, we have developed an solid amino‐supported Pd catalyst for the diastereoselective construction of cyclopentenols with an alkynyl group, which are key substructures and synthons of many bioactive compounds. This heterogeneous catalyst exhibited high activity and selectivity, as well as good recyclability. It was demonstrated that the amino support interacts with Pd, on one hand improving the chemoselectivity of the heterogeneous palladium catalyst, and on the other hand contributing to the high palladium activity in the domino reaction by protecting the Pd species from aggregating into Pd black. The chemoselectivity of the domino reactions was readily controlled by switching the catalyst or solvent, which allows chemodivergent syntheses of **2**, **4** and **5** in excellent yields. The domino strategy developed was also extended to the asymmetric syntheses as well as oxidative carbocyclization‐borylation reactions. It is expected that our work will stimulate more research on the development of heterogeneous catalytic system for highly active and selective transformations.

## Conflict of interest

The authors declare no conflict of interest.

## Supporting information

As a service to our authors and readers, this journal provides supporting information supplied by the authors. Such materials are peer reviewed and may be re‐organized for online delivery, but are not copy‐edited or typeset. Technical support issues arising from supporting information (other than missing files) should be addressed to the authors.

Supplementary
