# Supplementary material for: Amino‐Supported Palladium Catalyst for Chemo‐ and Stereoselective Domino Reactions
Source: Angew Chem Int Ed Engl. 2020 Nov 10;60(2):670–4. doi: 10.1002/anie.202011708 (PMC7839730; doi:10.1002/anie.202011708)

Angewandte Chemie
Eine Zeitschrift der Gesellschaft Deutscher Chemiker

Supporting Information

Amino-Supported Palladium Catalyst for Chemo- and Stereoselective Domino Reactions

Man-Bo Li[+], Jie Yang, Ying Yang, Guo-Yong Xu, Gen Luo, Jianping Yang[+], and Jan-E. Bäckvall**

anie_202011708_sm_miscellaneous_information.pdf

Supporting Information

Table of Contents

General information	S1
Control experiments on amine additive effect	S2
Typical procedure for the formation of **2**	S3-S12
Typical procedure for the formation of **3**	S13-S14
Typical procedure for the formation of **4**	S15-S17
Typical procedure for the formation of **5**	S18-S20
Recycling experiments and kinetic studies	S21
Hot filtration test	S22
Asymmetric syntheses of **2**, **4** and **5**	S23-S25
Typical procedure for the formation of **6**	S26
References	S27
^{1}H NMR and ^{13}C NMR spectra of compounds	S28-S62

General information

Unless otherwise noted, all reagents were used as received from commercial suppliers. Reactions were monitored using thin-layer chromatography (SiO_2). Pd-AmP-MCF[1] and Pd-AmP-CNC[2] were prepared as described in our previous report. Enallenes **1** were synthesized using our previously reported procedure.[3] TLC plates were visualized with UV light (254 nm) or $KMnO_4$ stain. Flash chromatography was carried out with 60Å (particle size 35-70 μm) normal flash silica gel. NMR spectra were recorded at 400 MHz (^1H) and at 100 MHz (^{13}C), respectively. Chemical shifts (δ) are reported in ppm, using the residual solvent peak in $CDCl_3$ (H = 7.26 and C = 77.0 ppm) as internal standard, and coupling constants (*J*) are given in Hz. HRMS were recorded using ESI-TOF techniques. The enantiomeric excess of compounds was determined by chiral HPLC using racemic compounds as references. The relative stereochemistry of compounds **2** was determined by NOE effect of NOESY specta of **2d** and **2m**. XPS measurements were performed on a Thermo ESCALAB 250 configured with a monochromated Al_{Ka} (1486.8 eV) 150 W X-ray source, 0.5 mm circular spot size and a flood gun to counter charging effects.

Control experiments on amine additive effect

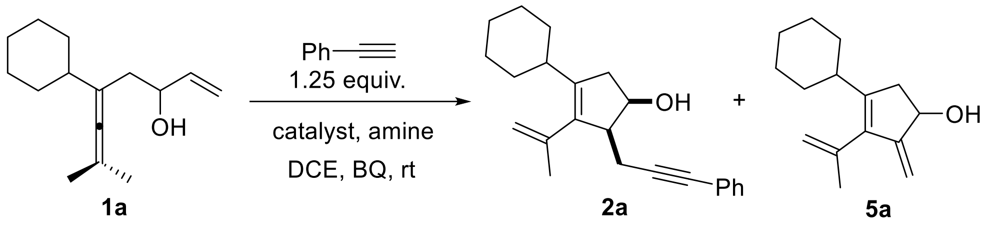

Amine (0.1 equiv.)		Catalyst (1 mol%) and Yield (%)			
		Pd-AmP-MCF		$Pd(OAc)_2$	
		**2a**	**5a**	**2a**	**5a**
	none	44	35	5[b]	35[b]
3° amine	Et_3N	84	4	50[b]	4[b]
	Pr_3N	82	4	46[b]	0[b]
	Bu_3N	80	5	42[b]	0[b]
	$^iPr_2EtN$	79	6	44[b]	0[b]
1° and 2° amine	Et_2NH	50	36	30[b]	12[b]
	Pr_2NH	47	38	28[b]	14[b]
	Bu_2NH	45	38	29[b]	15[b]
	$^iPr_2NH$	46	35	25[b]	10[b]
	$PrNH_2$	45	40	25[b]	15[b]
	$BuNH_2$	48	40	27[b]	16[b]
diamine	TMEDA	0[a]	0[a]	0[a]	0[a]
	1,10-Phen	0[a]	0[a]	0[a]	0[a]

Reaction conditions: **1a** (0.2 mmol), phenylacetylene (0.25 mmol), Pd catalyst (1 mol%), amine additive (0.1 equiv.), BQ (1.1 equiv.), DCE (1.0 mL), 8 h. [a] **1a** was recovered in >90% yield. [b] **1a** was partially recovered.

Typical procedure for the formation of 2

Preparation of

4-cyclohexyl-2-(3-phenylprop-2-yn-1-yl)-3-(prop-1-en-2-yl)cyclopent-3-en-1-ol (**2a**):

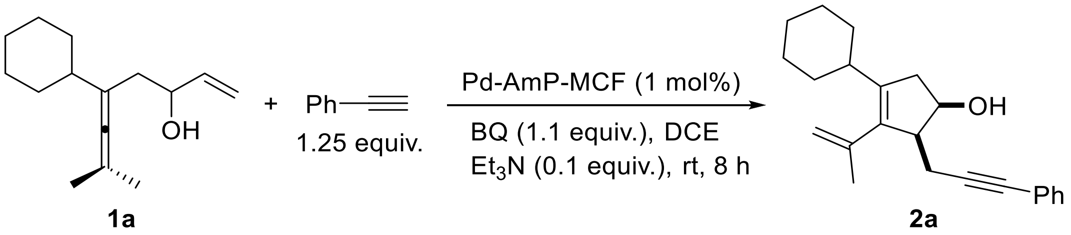

To a solution of Pd-AmP-MCF (4.1 mg, 0.002 mmol), Et_3N (2.8 μL mg, 0.02 mmol) and BQ (23.8 mg, 0.22 mmol) in DCE (1.0 mL) was added enallene **1a** (44.0 mg, 0.2 mmol) and phenylacetylene (25.5 mg, 0.25 mmol). The reaction mixture was stirred at room temperature for 8 h and then the solvent was evaporated under vacuum. The residue was purified via column chromatography on silica gel (eluent: petroleum ether/ethyl acetate = 15/1) to afford **2a** (52.9 mg, 83%, >20:1 d.r.), colorless oil. ^1H NMR (400 MHz, $CDCl_3$) δ 7.38 – 7.35 (m, 2H), 7.28 – 7.25 (m, 3H), 5.03 – 5.01 (m, 1H), 4.76 – 4.70 (m, 1H), 4.33 – 4.31 (m, 1H), 2.91 – 2.84 (m, 1H), 2.77 (dd, J = 16.8, 6.8 Hz, 1H), 2.64 (dd, J = 16.8, 4.2 Hz, 1H), 2.50 – 2.42 (m, 1H), 2.32 – 2.23 (m, 2H), 1.93 (s, 1H), 1.85 – 1.81 (m, 3H), 1.72 – 1.62 (m, 3H), 1.54 – 1.48 (m, 2H), 1.31 – 1.16 (m, 5H); ^{13}C NMR (100 MHz, $CDCl_3$) δ 141.1, 140.5, 136.4, 131.5, 128.2, 127.7, 123.7, 114.6, 88.4, 81.7, 76.1, 55.3, 40.3, 38.0, 32.0, 31.6, 26.3, 26.2, 26.1, 22.6, 22.1; HRMS (ESI): calc. for $C_{23}H_{28}NaO$ $[M+Na]^+$: 343.2032; found: 343.2036.

The general method above was used for the preparation of the following compounds:

4-Cyclohexyl-2-(3-(4-methoxyphenyl)prop-2-yn-1-yl)-3-(prop-1-en-2-yl)cyclopent-3-en-1-ol (**2b**)

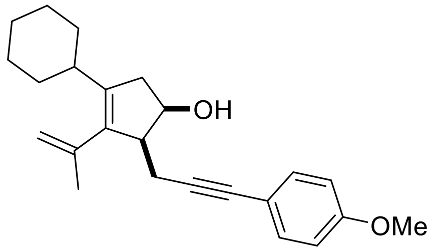

2b

85% isolated yield (>20:1 d.r.), colorless oil. ^1H NMR (400 MHz, $CDCl_3$) δ 7.32 – 7.28 (m, 2H), 6.82 – 6.79 (m, 2H), 5.02 – 5.00 (m, 1H), 4.74 – 4.71 (m, 1H), 4.32 –

4.26 (m, 1H), 3.80 (s, 3H), 2.90 – 2.82 (m, 1H), 2.76 (dd, *J* = 16.8, 7.0 Hz, 1H), 2.62 (dd, *J* = 16.8, 4.2 Hz, 1H), 2.49 – 2.42 (m, 1H), 2.31 – 2.19 (m, 2H), 1.98 (s, 1H), 1.82 (s, 3H), 1.72 – 1.63 (m, 3H), 1.54 – 1.46 (m, 2H), 1.31 – 1.18 (m, 5H); ^{13}C NMR (100 MHz, $CDCl_3$) δ 159.1, 140.9, 140.5, 136.4, 132.8, 115.8, 114.6, 113.9, 86.7, 81.4, 76.2, 55.3, 55.3, 40.1, 38.0, 32.0, 31.6, 26.3, 26.2, 26.1, 22.6, 22.2; HRMS (ESI): calc. for $C_{24}H_{30}NaO_2$ [M+Na]$^+$: 373.2138; found: 373.2140.

4-Cyclohexyl-2-(3-(2-methoxyphenyl)prop-2-yn-1-yl)-3-(prop-1-en-2-yl)cyclopent-3-en-1-ol (**2c**)

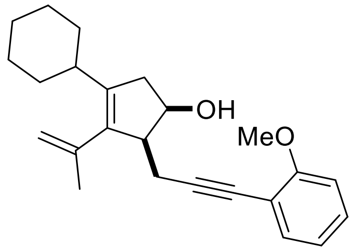

2c

76% isolated yield (>20:1 d.r.), colorless oil. ^{1}H NMR (400 MHz, $CDCl_3$) δ 7.36 – 7.33 (m, 1H), 7.26 – 7.21 (m, 1H), 6.91 – 6.84 (m, 2H), 4.99 – 4.97 (m, 1H), 4.69 – 4.66 (m, 1H), 4.36 – 4.29 (m, 1H), 3.88 (s, 3H), 3.38 (d, *J* = 2.9 Hz, 1H), 2.96 – 2.88 (m, 1H), 2.77 – 2.67 (m, 2H), 2.43 – 2.36 (m, 1H), 2.33 – 2.31 (m, 1H), 2.16 (dd, *J* = 16.8, 11.0 Hz, 1H), 1.79 – 1.75 (m, 3H), 1.73 – 1.63 (m, 3H), 1.50 – 1.47 (m, 2H), 1.28 – 1.09 (m, 5H); ^{13}C NMR (100 MHz, $CDCl_3$) δ 159.8, 140.5, 140.5, 136.6, 132.9, 129.1, 120.6, 114.4, 112.6, 110.3, 93.1, 78.1, 55.6, 54.6, 39.2, 38.0, 32.0, 31.5, 26.4, 26.2, 26.1, 23.3, 22.4; HRMS (ESI): calc. for $C_{24}H_{30}NaO_2$ [M+Na]$^+$: 373.2138; found: 373.2131.

4-Cyclohexyl-2-(3-(3-methoxyphenyl)prop-2-yn-1-yl)-3-(prop-1-en-2-yl)cyclopent-3-en-1-ol (**2d**)

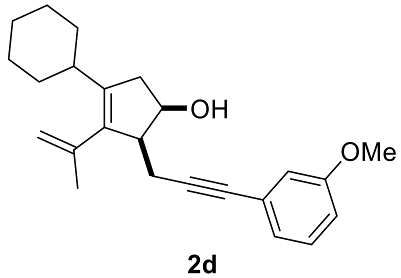

2d

80% isolated yield (>20:1 d.r.), colorless oil. ^{1}H NMR (400 MHz, $CDCl_3$) δ 7.18 (t,

J = 7.6 Hz, 1H), 6.96 (d, *J* = 7.6 Hz, 1H), 6.92 – 6.87 (m, 1H), 6.86 – 6.80 (m, 1H), 5.04 – 5.03 (m, 1H), 4.75 – 4.73 (m, 1H), 4.35 – 4.31 (m, 1H), 3.78 (s, 3H), 2.89 – 2.87 (m, 1H), 2.77 (dd, *J* = 16.8, 6.9 Hz, 1H), 2.64 (dd, *J* = 16.8, 4.2 Hz, 1H), 2.30 – 2.26 (m, 2H), 1.91 (d, *J* = 4.8 Hz, 1H), 1.85 – 1.83 (m, 3H), 1.74 – 1.63 (m, 3H), 1.55 – 1.51 (m, 2H), 1.33 – 1.23 (m, 5H); ^{13}C NMR (100 MHz, $CDCl_3$) δ 159.3, 141.1, 140.5, 136.4 129.3, 124.7, 124.1, 116.4, 114.6, 114.3, 88.3, 81.6, 76.0, 55.3, 55.2, 40.3, 38.0, 32.0, 31.6, 26.3, 26.2, 26.1, 22.7, 22.1; HRMS (ESI): calc. for $C_{24}H_{30}NaO_2$ $[M+Na]^+$: 373.2138; found: 373.2130.

4-Cyclohexyl-2-(3-(4-nitrophenyl)prop-2-yn-1-yl)-3-(prop-1-en-2-yl)cyclopent-3-en-1-ol (**2e**)

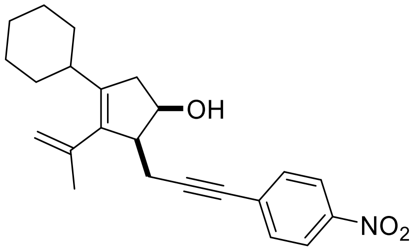

2e

79% isolated yield (>20:1 d.r.), colorless oil. ^{1}H NMR (400 MHz, $CDCl_3$) δ 8.18 – 8.11 (m, 2H), 7.51 – 7.45 (m, 2H), 5.05 – 5.03 (m, 1H), 4.76 – 4.70 (m, 1H), 4.34 – 4.27 (m, 1H), 2.95 – 2.87 (m, 1H), 2.78 (dd, *J* = 16.8, 6.8 Hz, 1H), 2.67 (dd, *J* = 16.8, 4.3 Hz, 1H), 2.38 – 2.27 (m, 2H), 1.86 – 1.81 (m, 3H), 1.76 – 1.64 (m, 4H), 1.54 – 1.45 (m, 2H), 1.29 – 1.13 (m, 5H); ^{13}C NMR (100 MHz, $CDCl_3$) δ 141.5, 140.3, 136.2, 133.4, 132.2, 130.8, 123.5, 114.8, 94.7, 80.2, 75.6, 55.2, 40.7, 38.0, 32.0, 31.6, 26.3, 26.2, 26.1, 22.7, 22.2; HRMS (ESI): calc. for $C_{23}H_{27}NNaO_3$ $[M+Na]^+$: 388.1883; found: 388.1880 (Note: Because the polarity of terminal alkyne homocoupling product is very similar with **2e**, it is hard to isolate pure **2e**. The yield is based on impure material).

2-(3-(4-Bromophenyl)prop-2-yn-1-yl)-4-cyclohexyl-3-(prop-1-en-2-yl)cyclopent-3-en-1-ol (**2f**)

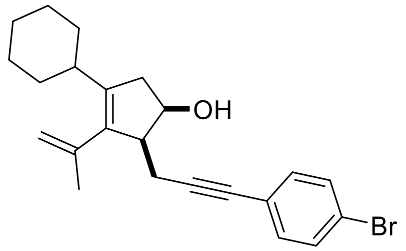

2f

70% isolated yield (>20:1 d.r.), colorless oil. ^{1}H NMR (400 MHz, $CDCl_3$) δ 7.42 – 7.39 (m, 2H), 7.23 – 7.19 (m, 2H), 5.04 – 5.01 (m, 1H), 4.75 – 4.72 (m, 1H), 4.34 – 4.26 (m, 1H), 2.89 – 2.85 (m, 1H), 2.76 (dd, J = 17.2, 6.4 Hz, 1H), 2.61 (dd, J = 17.2, 4.3 Hz, 1H), 2.49 – 2.43 (m, 1H), 2.31 – 2.21 (m, 2H), 1.85 – 1.83 (m, 3H), 1.73 – 1.65 (m, 3H), 1.53 – 1.48 (m, 2H), 1.30 – 1.21 (m, 5H); ^{13}C NMR (100 MHz, $CDCl_3$) δ 141.2, 140.4, 136.3, 133.8, 133.0, 131.8, 131.5, 114.7, 89.7, 80.6, 75.9, 55.3, 40.4, 38.0, 32.0, 31.6, 26.3, 26.2, 26.1, 22.7, 22.1; HRMS (ESI): calc. for $C_{23}H_{27}BrNaO$ $[M+Na]^+$: 421.1137; found: 421.1129.

2-(3-(4-Chlorophenyl)prop-2-yn-1-yl)-4-cyclohexyl-3-(prop-1-en-2-yl)cyclopent-3-en-1-ol (**2g**)

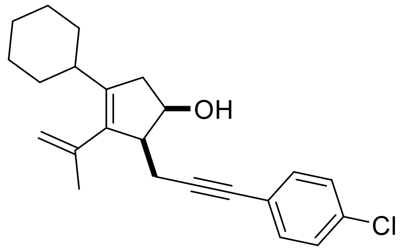

2g

67% isolated yield (>20:1 d.r.), colorless oil. ^{1}H NMR (400 MHz, $CDCl_3$) δ 7.49 – 7.43 (m, 1H), 7.33 – 7.27 (m, 3H), 5.04 – 5.01 (m, 1H), 4.77 – 4.70 (m, 1H), 4.33 – 4.27 (m, 1H), 2.89 – 2.84 (m, 1H), 2.76 (dd, J = 16.8, 6.8 Hz, 1H), 2.62 (dd, J = 16.8, 4.3 Hz, 1H), 2.49 – 2.40 (m, 1H), 2.32 – 2.23 (m, 2H), 1.85 – 1.82 (m, 3H), 1.75 – 1.68 (m, 3H), 1.55 – 1.48 (m, 4H), 1.32 – 1.22 (m, 5H); ^{13}C NMR (100 MHz, $CDCl_3$) δ 141.2, 140.4, 136.3, 133.7, 132.7, 128.5, 122.2, 114.7, 89.5, 80.6, 75.9, 55.3, 40.4, 38.0, 32.0, 31.6, 26.3, 26.2, 26.1, 22.7, 22.1; HRMS (ESI): calc. for $C_{23}H_{27}ClNaO$ $[M+Na]^+$: 377.1643; found: 377.1645.

Methyl

4-(3-(-3-cyclohexyl-5-hydroxy-2-(prop-1-en-2-yl)cyclopent-2-en-1-yl)prop-1-yn-1-yl)b

enzoate (**2h**)

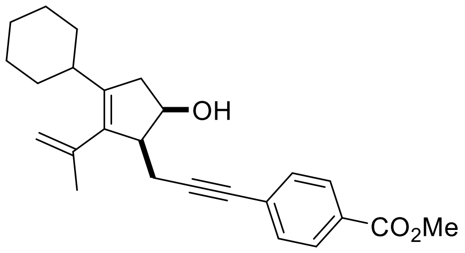

2h

76% isolated yield (>20:1 d.r.), colorless oil. ^1H NMR (400 MHz, $CDCl_3$) δ 7.98 – 7.93 (m, 2H), 7.43 – 7.36 (m, 2H), 5.05 – 5.01 (m, 1H), 4.76 – 4.71 (m, 1H), 4.34 – 4.27 (m, 1H), 3.91 (s, 3H), 2.93 – 2.86 (m, 1H), 2.78 (dd, J = 16.8, 6.8 Hz, 1H), 2.66 (dd, J = 16.8, 4.3 Hz, 1H), 2.49 – 2.43 (m, 1H), 2.34 – 2.26 (m, 2H), 1.84 – 1.82 (m, 3H), 1.72 – 1.61 (m, 3H), 1.55 – 1.48 (m, 2H), 1.29 – 1.23 (m, 5H); ^{13}C NMR (100 MHz, $CDCl_3$) δ 166.6, 141.3, 140.4, 136.3, 131.4, 129.4, 129.0, 128.5, 114.7, 91.9, 81.1, 75.8, 55.3, 52.2, 40.5, 38.0, 32.0, 31.6, 26.3, 26.2, 26.1, 22.7, 22.2; HRMS (ESI): calc. for $C_{25}H_{30}NaO_3$ $[M+Na]^+$: 401.2087; found: 401.2079.

2-(3-(4-Fluorophenyl)prop-2-yn-1-yl)-3-(prop-1-en-2-yl)-4-propylcyclopent-3-en-1-ol (**2i**)

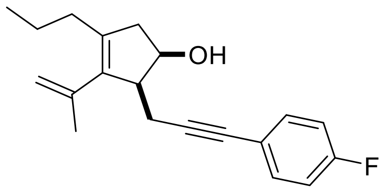

2i

80% isolated yield (>20:1 d.r.), colorless oil. ^1H NMR (400 MHz, $CDCl_3$) δ 7.36 – 7.30 (m, 2H), 6.99 – 6.94 (m, 2H), 5.05 – 5.01 (m, 1H), 4.78 – 4.74 (m, 1H), 4.37 – 4.30 (m, 1H), 2.94 – 2.87 (m, 1H), 2.81 (dd, J = 17.6, 6.7 Hz, 1H), 2.62 (dd, J = 17.6, 4.2 Hz, 1H), 2.32 – 2.20 (m, 2H), 2.15 (t, J = 7.6 Hz, 2H), 1.86 – 1.83 (m, 3H), 1.45 – 1.36 (m, 2H), 0.88 (t, J = 7.6 Hz, 3H); ^{13}C NMR (100 MHz, $CDCl_3$) δ 162.14 (d, J_{C-F} = 247 Hz), 140.20, 137.93, 136.31, 133.29 (d, J_{C-F} = 8 Hz), 119.73 (d, J_{C-F} = 4 Hz), 115.45 (d, J_{C-F} = 21 Hz), 114.9, 88.1, 80.5, 75.9, 55.7, 43.9, 31.2, 22.5, 22.0, 21.4, 14.0; HRMS (ESI): calc. for $C_{20}H_{23}FNaO$ $[M+Na]^+$: 321.1625; found: 321.1620.

3-(Prop-1-en-2-yl)-4-propyl-2-(3-(4-(trifluoromethyl)phenyl)prop-2-yn-1-yl)cyclopent -3-en-1-ol (**2j**)

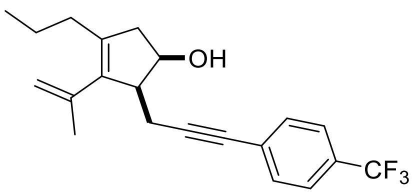

2j

83% isolated yield (>20:1 d.r.), colorless oil. ^{1}H NMR (400 MHz, $CDCl_3$) δ 7.53 (d, *J* = 8.2 Hz, 2H), 7.45 (d, *J* = 8.2 Hz, 2H), 5.05 – 5.02 (m, 1H), 4.81 – 4.74 (m, 1H), 4.35 – 4.32 (m, 1H), 2.95 – 2.91 (m, 1H), 2.82 (dd, *J* = 17.4, 6.4 Hz, 1H), 2.66 (dd, *J* = 17.4, 4.3 Hz, 1H), 2.35 – 2.25 (m, 2H), 2.16 (t, *J* = 7.4 Hz, 2H), 1.86 – 1.83 (m, 3H), 1.80 (d, *J* = 4.9 Hz, 1H), 1.44 – 1.40 (m, 2H), 0.88 (t, *J* = 7.4 Hz, 3H); ^{13}C NMR (100 MHz, $CDCl_3$) δ 140.1, 137.8, 136.5, 131.7, 129.6, 129.3, 127.6, 125.2(q, J_{C-F} = 4 Hz), 115.0, 91.3, 80.5, 75.7, 55.6, 44.1, 31.2, 22.5, 22.1, 21.4, 14.0; HRMS (ESI): calc. for $C_{21}H_{23}F_3NaO$ [M+Na]$^+$: 371.1593; found: 371.1589.

3-(Prop-1-en-2-yl)-4-propyl-2-(3-(thiophen-2-yl)prop-2-yn-1-yl)cyclopent-3-en-1-ol (**2k**)

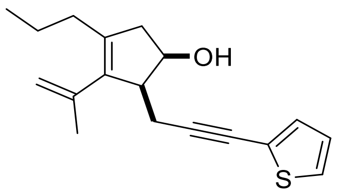

2k

73% isolated yield (>20:1 d.r.), colorless oil. ^{1}H NMR (400 MHz, $CDCl_3$) δ 7.17 (dd, *J* = 5.2, 1.1 Hz, 1H), 7.10 (dd, *J* = 3.6, 1.1 Hz, 1H), 6.93 (dd, *J* = 5.2, 3.6 Hz, 1H), 5.05 – 5.01 (m, 1H), 4.82 – 4.73 (m, 1H), 4.36 – 4.28 (m, 1H), 2.93 – 2.89 (m, 1H), 2.82 (dd, *J* = 17.2, 6.4 Hz, 1H), 2.65 (dd, *J* = 17.2, 4.2 Hz, 1H), 2.34 – 2.24 (m, 2H), 2.15 (t, *J* = 7.6 Hz, 2H), 1.86 – 1.82 (m, 3H), 1.45 – 1.40 (m, 2H), 0.88 (t, *J* = 7.6 Hz, 3H); ^{13}C NMR (100 MHz, $CDCl_3$) δ 140.2, 137.8, 136.5, 131.1, 126.8, 126.1, 123.8, 114.9, 92.5, 75.8, 74.7, 55.7, 44.0, 31.2, 22.5, 22.4, 21.4, 14.0; HRMS (ESI): calc. for $C_{18}H_{22}NaOS$ [M+Na]$^+$: 309.1284; found: 309.1281.

3-(Prop-1-en-2-yl)-4-propyl-2-(3-(thiophen-3-yl)prop-2-yn-1-yl)cyclopent-3-en-1-ol (**2l**)

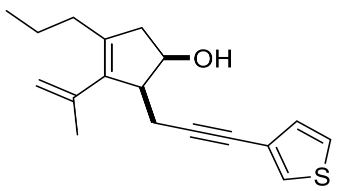

2l

75% isolated yield (>20:1 d.r.), colorless oil. ^{1}H NMR (400 MHz, $CDCl_3$) δ 7.33 (dd, *J* = 3.0, 1.1 Hz, 1H), 7.23 (dd, *J* = 5.0, 3.0 Hz, 1H), 7.04 (dd, *J* = 5.0, 1.1 Hz, 1H), 5.04 – 5.00 (m, 1H), 4.78 – 4.72 (m, 1H), 4.38 – 4.31 (m, 1H), 2.94 – 2.87 (m, 1H), 2.81 (dd, *J* = 17.2, 6.4 Hz, 1H), 2.62 (dd, *J* = 17.2, 4.2 Hz, 1H), 2.30 – 2.12 (m, 4H), 1.89 (d, *J* = 4.5 Hz, 1H), 1.86 – 1.83 (m, 3H), 1.45 – 1.40 (m, 2H), 0.88 (t, *J* = 7.4 Hz, 3H); ^{13}C NMR (100 MHz, $CDCl_3$) δ 140.2, 137.9, 136.3, 129.9, 127.8, 125.1, 122.6, 114.8, 87.9, 75.9, 55.7, 43.8, 31.2, 22.5, 22.1, 21.4, 14.0; HRMS (ESI): calc. for $C_{18}H_{22}NaOS$ $[M+Na]^+$: 309.1284; found: 309.1277.

2-(3-(4-Methoxyphenyl)prop-2-yn-1-yl)-3-(prop-1-en-2-yl)-4-propylcyclopent-3-en-1-ol (**2m**)

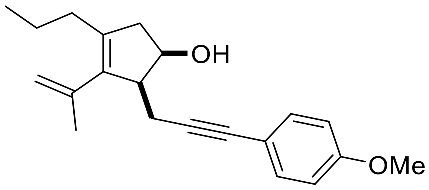

2m

84% isolated yield (>20:1 d.r.), colorless oil. ^{1}H NMR (400 MHz, $CDCl_3$) δ 7.34 – 7.28 (m, 2H), 6.84 – 6.78 (m, 2H), 5.03 – 4.99 (m, 1H), 4.79 – 4.73 (m, 1H), 4.40 – 4.29 (m, 1H), 3.80 (s, 3H), 2.93 – 2.86 (m, 1H), 2.80 (dd, *J* = 16.8, 7.1 Hz, 1H), 2.63 (dd, *J* = 16.8, 4.2 Hz, 1H), 2.30 – 2.11 (m, 4H), 1.45 – 1.37 (m, 2H), 0.88 (t, *J* = 7.4 Hz, 3H); ^{13}C NMR (100 MHz, $CDCl_3$) δ 159.1, 140.2, 138.0, 136.2, 132.8, 115.8, 114.8, 113.9, 86.8, 81.4, 76.0, 55.7, 55.3, 43.7, 31.2, 22.5, 22.1, 21.4, 14.0; HRMS (ESI): calc. for $C_{21}H_{26}NaO_2$ $[M+Na]^+$: 333.1825; found: 333.1820.

4-Butyl-2-(3-phenylprop-2-yn-1-yl)-3-(prop-1-en-2-yl)cyclopent-3-en-1-ol (**2n**)

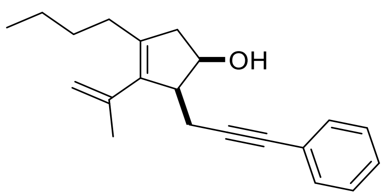

2n

81% isolated yield (>20:1 d.r.), colorless oil. ^{1}H NMR (400 MHz, $CDCl_3$) δ 7.38 – 7.35 (m, 2H), 7.29 – 7.26 (m, 3H), 5.04 – 5.00 (m, 1H), 4.79 – 4.75 (m, 1H), 4.38 – 4.32 (m, 1H), 2.94 – 2.88 (m, 1H), 2.83 (dd, J = 17.0, 6.1 Hz, 1H), 2.64 (dd, J = 17.0, 4.2 Hz, 1H), 2.31 – 2.15 (m, 4H), 1.89 (d, J = 3.5 Hz, 1H), 1.86 – 1.81 (m, 3H), 1.35 – 1.24 (m, 4H), 0.86 (d, J = 7.2 Hz, 3H); ^{13}C NMR (100 MHz, $CDCl_3$) δ 140.2, 137.7, 136.5, 131.5, 128.2, 127.7, 123.7, 114.8, 88.4, 81.6, 75.9, 55.7, 44.0, 30.5, 28.9, 22.6, 22.5, 22.1, 14.0; HRMS (ESI): calc. for $C_{21}H_{26}NaO$ $[M+Na]^+$: 317.1876; found: 317.1870.

4-Cyclopropyl-2-(3-phenylprop-2-yn-1-yl)-3-(prop-1-en-2-yl)cyclopent-3-en-1-ol (**2o**)

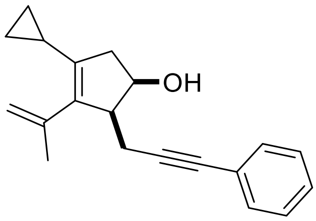

2o

84% isolated yield (>20:1 d.r.), colorless oil. ^{1}H NMR (400 MHz, $CDCl_3$) δ 7.41 – 7.36 (m, 2H), 7.32 – 7.26 (m, 3H), 5.12 – 5.06 (m, 1H), 4.96 – 4.92 (m, 1H), 4.33 – 4.28 (m, 1H), 2.98 – 2.91 (m, 1H), 2.65 (dd, J = 16.8, 4.1 Hz, 1H), 2.50 (dd, J = 16.8, 6.8 Hz, 1H), 2.26 (dd, J = 16.8, 6.8 Hz, 1H), 2.02 (dd, J = 16.8, 4.1 Hz, 1H), 1.92 – 1.82 (m, 5H), 0.70 – 0.61 (m, 3H), 0.54 – 0.48 (m, 1H); ^{13}C NMR (100 MHz, $CDCl_3$) δ 140.2, 137.4, 136.8, 131.5, 128.2, 127.7, 123.6, 115.1, 88.4, 81.7, 75.5, 56.2, 39.9, 22.5, 22.1 11.2, 5.2, 5.1; HRMS (ESI): calc. for $C_{20}H_{22}NaO$ $[M+Na]^+$: 301.1563; found: 301.1554.

4-(3-Phenylprop-2-yn-1-yl)-5-(prop-1-en-2-yl)-[1,1'-bi(cyclopentan)]-5-en-3-ol (**2p**)

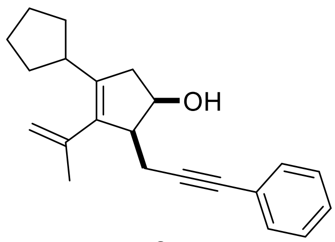

2p

85% isolated yield (>20:1 d.r.), colorless oil. ^{1}H NMR (400 MHz, $CDCl_3$) δ 7.38 – 7.35 (m, 2H), 7.29 – 7.26 (m, 3H), 5.05 – 5.00 (m, 1H), 4.79 – 4.72 (m, 1H), 4.38 – 4.32 (m, 1H), 2.94 – 2.87 (m, 2H), 2.78 (dd, *J* = 16.8, 7.0 Hz, 1H), 2.65 (dd, *J* = 16.8, 4.4 Hz, 1H), 2.34 – 2.24 (m, 2H), 1.94 (d, *J* = 4.7 Hz, 1H), 1.85 – 1.81 (m, 3H), 1.68 – 1.58 (m, 6H), 1.44 – 1.36 (m, 2H); ^{13}C NMR (100 MHz, $CDCl_3$) δ 140.5, 139.1, 137.5, 131.5, 128.2, 127.7, 123.6, 114.7, 88.4, 81.7, 76.1, 55.4, 39.8, 39.0, 32.1, 31.2, 26.0, 25.8, 22.5, 22.1; HRMS (ESI): calc. for $C_{22}H_{26}NaO$ $[M+Na]^+$: 329.1876; found: 329.1869.

2-(3-(4-Methoxyphenyl)prop-2-yn-1-yl)-4-phenethyl-3-(prop-1-en-2-yl)cyclopent-3-en-1-ol (**2q**)

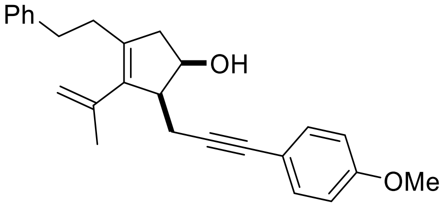

2q

80% isolated yield (>20:1 d.r.), colorless oil. ^{1}H NMR (400 MHz, $CDCl_3$) δ 7.33 – 7.29 (m, 2H), 7.26 – 7.13 (m, 5H), 6.83 – 6.79 (m, 2H), 5.02 – 4.96 (m, 1H), 4.68 – 4.62 (m, 1H), 4.37 – 4.30 (m, 1H), 3.80 (s, 3H), 2.86 (d, *J* = 7.2 Hz, 1H), 2.70 (d, *J* = 7.9 Hz, 2H), 2.62 – 2.57 (m, 1H), 2.53 – 2.45 (m, 2H), 2.33 (dd, *J* = 16.8, 3.6 Hz, 1H), 2.20 (dd, *J* = 16.8, 9.2 Hz, 1H), 1.89 (d, *J* = 4.8 Hz, 1H), 1.76 – 1.70 (m, 3H); ^{13}C NMR (100 MHz, $CDCl_3$) δ 159.2, 141.9, 140.1, 139.0, 135.1, 132.9, 128.3, 128.3 125.9, 115.8, 114.9, 113.9, 86.7, 81.4, 76.0, 55.6, 55.3, 43.8, 34.6, 31.0, 22.2, 22.0; HRMS (ESI): calc. for $C_{26}H_{28}NaO_2$ $[M+Na]^+$: 395.1982; found: 395.1977.

4-Cyclohexyl-2-(3-(4-(dimethylamino)phenyl)prop-2-yn-1-yl)-3-(prop-1-en-2-yl)cyclopent-3-en-1-ol (**2r**)

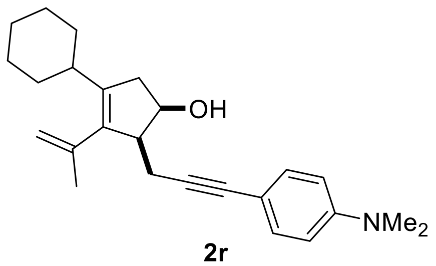

2r

84% isolated yield (>20:1 d.r., Et_3N is not necessary in this reaction), colorless oil. ^{1}H NMR (400 MHz, $CDCl_3$) δ 7.25 – 7.21 (m, 2H), 6.64 – 6.57 (m, 2H), 5.02 – 4.97 (m, 1H), 4.73 – 4.70 (m, 1H), 4.32 – 4.29 (m, 1H), 2.94 (s, 6H), 2.86 – 2.82 (m, 1H), 2.78 – 2.70 (m, 1H), 2.62 (dd, *J* = 16.8, 4.2 Hz, 1H), 2.48 – 2.39 (m, 1H), 2.29 – 2.16 (m, 2H), 1.83 – 1.80 (m, 3H), 1.71 – 1.37 (m, 7H), 1.26 – 1.20 (m, 5H); ^{13}C NMR (100 MHz, $CDCl_3$) δ 140.9, 140.7, 136.6, 133.7, 132.6, 114.6, 112.0, 111.8, 85.8, 82.4, 76.5, 55.4, 40.4, 40.2, 38.1, 32.1, 31.7, 26.4, 26.3, 26.2, 22.7, 22.4; HRMS (ESI): calc. for $C_{25}H_{33}NNaO$ $[M+Na]^+$: 386.2454; found: 386.2462. (Note: Because the polarity of terminal alkyne homocoupling product is very similar with **2r**, it is hard to isolate pure **2r**. The isolated yield is based on the ^{1}H NMR ratio of **2r** and alkyne homocoupling product)

Typical procedure for the formation of 3

Preparation of 4-cyclohexyl-6,6-dimethyl-2-vinyl-3,6-dihydro-2H-pyran (**3a**):

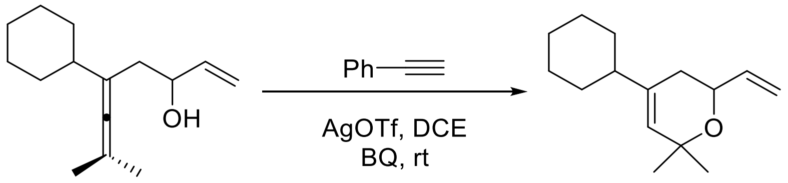

1a **3a**

To a solution of AgOTf (2.57 mg, 0.01 mmol) and BQ (23.8 mg, 0.22 mmol) in DCE (1.0 mL) was added enallene **1a** (44.0 mg, 0.2 mmol) and phenylacetylene (25.5 mg, 0.25 mmol). The reaction mixture was stirred at room temperature for 12 h and then the solvent was evaporated under vacuum. The residue was purified via column chromatography on silica gel (eluent: petroleum ether/ethyl acetate = 50/1) to afford **3a** (39.3 mg, 89%), colorless oil. ^{1}H NMR (400 MHz, $CDCl_3$) δ 5.92 (ddd, *J* = 17.2, 10.4, 6.0 Hz, 1H), 5.30 – 5.22 (m, 2H), 5.16 – 5.08 (m, 1H), 4.15 – 4.06 (m, 1H), 2.00 (ddd, *J* = 16.5, 10.5, 2.3 Hz, 1H), 1.87 – 1.65 (m, 7H), 1.29 – 1.08 (m, 11H); ^{13}C NMR (100 MHz, $CDCl_3$) δ 139.6, 138.5, 126.5, 115.1, 72.9, 70.0, 44.7, 32.3, 31.8, 31.2, 30.0, 26.6, 26.5, 26.4, 26.3; HRMS (ESI): calc. for $C_{15}H_{24}NaO$ $[M+Na]^+$: 243.1719; found: 243.1711.

The general method above was used for the preparation of the following compounds:

4-Cyclopentyl-6,6-dimethyl-2-vinyl-3,6-dihydro-2H-pyran (**3b**)

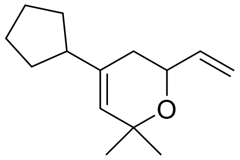

3b

90% isolated yield, colorless oil. ^{1}H NMR (400 MHz, $CDCl_3$) δ 5.87 (ddd, *J* = 17.2, 10.5, 5.7 Hz, 1H), 5.55 (s, 1H), 5.23 (dt, *J* = 17.2, 1.6 Hz, 1H), 5.07 (dt, *J* = 10.5, 1.5 Hz, 1H), 4.14 – 3.98 (m, 1H), 2.41 (dd, *J* = 13.6, 8.4 Hz, 1H), 2.28 – 2.18 (m, 3H), 2.11 – 2.04 (m, 2H), 1.79 – 1.75 (m, 3H), 1.56 – 1.47 (m, 8H); ^{13}C NMR (100 MHz, $CDCl_3$) δ 142.9, 142.5, 138.8, 114.2, 83.2, 76.1, 46.7, 30.1, 26.9, 24.9, 24.8, 22.9, 21.2, 14.0; HRMS (ESI): calc. for $C_{14}H_{22}NaO$ $[M+Na]^+$: 229.1563; found: 229.1559.

6,6-Dimethyl-4-phenyl-2-vinyl-3,6-dihydro-2H-pyran (**3c**)

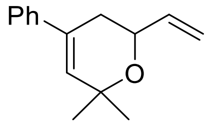

3c

95% isolated yield, colorless oil. ^{1}H NMR (400 MHz, $CDCl_3$) δ 7.41 – 7.25 (m, 5H), 6.11 – 5.92 (m, 2H), 5.36 (dt, *J* = 17.3, 1.5 Hz, 1H), 5.21 (dt, *J* = 10.5, 1.4 Hz, 1H), 4.33 – 4.29 (m, 1H), 2.49 – 2.32 (m, 2H), 1.37 (d, *J* = 1.2 Hz, 6H); ^{13}C NMR (100 MHz, $CDCl_3$) δ 140.5, 139.2, 132.4, 131.1, 128.5, 127.3, 125.0, 115.7, 73.5, 70.1, 32.7, 29.8, 26.1; HRMS (ESI): calc. for $C_{15}H_{18}NaO$ $[M+Na]^+$: 237.1250; found: 237.1244.

(E)-4-butyl-6,6-dimethyl-2-styryl-3,6-dihydro-2H-pyran (**3d**)

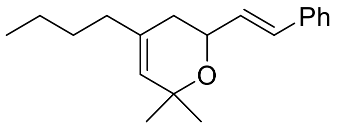

3d

88% isolated yield, colorless oil. ^{1}H NMR (400 MHz, $CDCl_3$) δ 7.42 – 7.36 (m, 2H), 7.33 – 7.27 (m, 2H), 7.24 – 7.19 (m, 1H), 6.62 (dd, *J* = 16.0, 1.2 Hz, 1H), 6.30 (dd, *J* = 16.0, 6.3 Hz, 1H), 5.36 – 5.30 (m, 1H), 4.36 – 4.39 (m, 1H), 2.17 – 2.05 (m, 1H), 2.03 – 1.95 (m, 2H), 1.89 (dd, *J* = 16.6, 3.2 Hz, 1H), 1.45 – 1.24 (m, 10H), 0.91 (t, *J* = 7.2 Hz, 3H); ^{13}C NMR (100 MHz, $CDCl_3$) δ 149.9, 144.2, 141.3, 139.8, 137.2, 128.3, 121.3, 116.2, 89.9, 44.3, 30.3, 29.4, 23.1, 22.7, 22.6, 14.0, 13.9; HRMS (ESI): calc. for $C_{19}H_{26}NaO$ $[M+Na]^+$: 293.1876; found: 293.1887.

Typical procedure for the formation of 4

Preparation of (Z)-7-methyl-5-propyl-6-((4-(trifluoromethyl)phenyl)ethynyl)octa-1,5,7-trien-3-ol (**4a**):

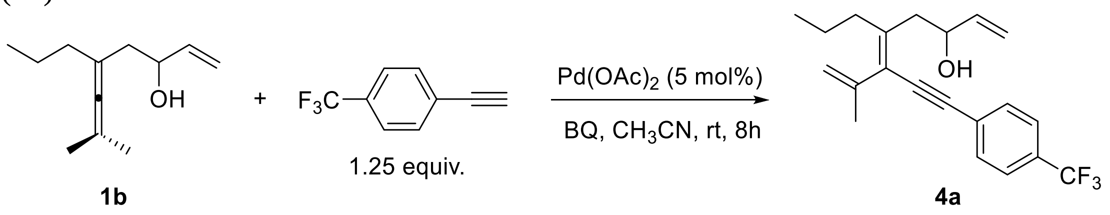

To a solution of $Pd(OAc)_2$ (2.25 mg, 0.01 mmol) and BQ (23.8 mg, 0.22 mmol) in CH_3CN (1.0 mL) was added enallene **1b** (36.0 mg, 0.2 mmol) and 4-fluorophenylacetylene (30.0 mg, 0.25 mmol). The reaction mixture was stirred at room temperature for 8 h and then the solvent was evaporated under vacuum. The residue was purified via column chromatography on silica gel (eluent: petroleum ether/ethyl acetate = 15/1) to afford **4a** (60.0 mg, 86%), colorless oil. ^{1}H NMR (400 MHz, $CDCl_3$) δ 7.57 – 7.49 (m, 4H), 5.97 (ddd, *J* = 17.2, 10.4, 6.0 Hz, 1H), 5.28 (dt, *J* = 17.2, 1.2 Hz, 1H), 5.13 (dt, *J* = 10.4, 1.2 Hz, 1H), 5.11 – 5.04 (m, 1H), 4.89 – 4.81 (m, 1H), 4.47 – 4.37 (m, 1H), 2.80 – 2.61 (m, 2H), 2.38 – 2.22 (m, 2H), 1.98 – 1.91 (m, 3H), 1.73 (d, *J* = 4.3 Hz, 1H), 1.49 – 1.41 (m, 2H), 0.92 (t, *J* = 7.3 Hz, 3H); ^{13}C NMR (100 MHz, $CDCl_3$) δ 147.3, 142.2, 140.8, 131.4, 129.7, 129.4, 127.5, 125.2 (q, $J_{C\text{-}F}$ = 4 Hz), 123.8, 115.2, 114.7, 91.9, 91.2, 72.4, 41.5, 34.8, 22.9, 22.0, 14.2; HRMS (ESI): calc. for $C_{21}H_{23}F_3NaO$ $[M+Na]^+$: 371.1593; found: 371.1599.

The general method above was used for the preparation of the following compounds:

(Z)-9-phenyl-6-(prop-1-en-2-yl)-5-propylnona-1,5-dien-7-yn-3-ol (**4b**)

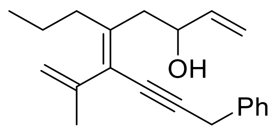

4b

77% isolated yield, colorless oil. ^{1}H NMR (400 MHz, $CDCl_3$) δ 7.38 – 7.28 (m, 5H), 5.88 (ddd, *J* = 17.2, 10.4, 6.0 Hz, 1H), 5.22 (dt, *J* = 17.2, 1.2 Hz, 1H), 5.08 (dt, *J* = 10.4, 1.2 Hz, 1H), 5.03 – 5.00 (m, 1H), 4.85 – 4.80 (m, 1H), 4.39 – 4.30 (m, 1H), 3.77 (s, 2H), 2.69 (dd, *J* = 13.2, 8.4 Hz, 1H), 2.52 (dd, *J* = 13.2, 5.4 Hz, 1H), 2.26 –

2.11 (m, 2H), 1.93 – 1.89 (m, 3H), 1.71 (d, J = 4.5 Hz, 1H), 1.44 – 1.37 (m, 2H), 0.89 (t, J = 7.4 Hz, 3H); ^{13}C NMR (100 MHz, $CDCl_3$) δ 144.1, 143.0, 140.9 137.1, 128.5, 127.9, 126.5, 124.3, 114.3, 114.3, 91.4, 81.7, 72.2, 41.2, 34.4, 26.0, 22.8, 22.0, 14.2; HRMS (ESI): calc. for $C_{21}H_{26}NaO$ $[M+Na]^+$: 317.1876; found: 317.1869.

(E)-5-cyclopropyl-7-methyl-6-(phenylethynyl)octa-1,5,7-trien-3-ol (**4c**)

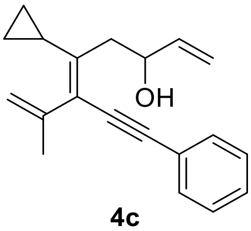

4c

70% isolated yield, colorless oil. ^{1}H NMR (400 MHz, $CDCl_3$) δ 7.45 – 7.35 (m, 2H), 7.36 – 7.24 (m, 3H), 5.98 (ddd, J = 16.8, 10.4, 6.0 Hz, 1H), 5.28 (d, J = 16.8 Hz, 1H), 5.18 – 5.03 (m, 3H), 4.50 – 4.42 (m, 1H), 2.40 (dd, J = 13.6, 8.4 Hz, 1H), 2.28 (dd, J = 13.6, 5.2 Hz, 1H), 2.23 – 2.18 (m, 3H), 1.96 – 1.86 (m, 2H), 0.80 – 0.58 (m, 4H); ^{13}C NMR (100 MHz, $CDCl_3$) δ 145.5, 142.2, 140.8, 131.2, 128.3, 127.9, 124.1, 123.7, 115.8, 114.3, 93.8, 89.1, 73.0, 38.0, 22.7, 14.7, 6.8, 6.7; HRMS (ESI): calc. for $C_{20}H_{22}NaO$ $[M+Na]^+$: 301.1563; found: 301.1566.

(Z)-5-(1-(cyclobut-1-en-1-yl)-3-phenylprop-2-yn-1-ylidene)non-1-en-3-ol (**4d**)

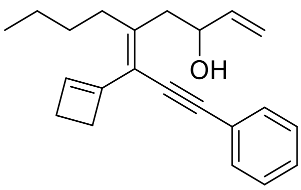

4d

80% isolated yield, colorless oil. ^{1}H NMR (400 MHz, $CDCl_3$) δ 7.45 – 7.28 (m, 5H), 6.21 – 6.15 (m, 1H), 5.99 (ddd, J = 17.2, 10.4, 5.6 Hz, 1H), 5.29 (dt, J = 17.2, 1.4 Hz, 1H), 5.13 (dt, J = 10.4, 1.4 Hz, 1H), 4.49 – 4.38 (m, 1H), 2.92 – 2.85 (m, 2H), 2.78 (dd, J = 13.2, 8.6 Hz, 1H), 2.67 (dd, J = 13.2, 5.2 Hz, 1H), 2.50 – 2.34 (m, 4H), 1.79 (d, J = 4.4 Hz, 1H), 1.48 – 1.34 (m, 4H), 0.94 (t, J = 7.2 Hz, 3H); ^{13}C NMR (100 MHz, $CDCl_3$) δ 147.5, 143.8, 140.9, 131.9, 131.3, 128.3, 127.9, 123.6, 117.0, 114.5, 93.1, 87.1, 72.5, 43.6, 32.6, 32.5, 31.2, 27.3, 23.0, 14.0; HRMS (ESI): calc. for $C_{22}H_{26}NaO$ $[M+Na]^+$: 329.1876; found: 329.1884.

(Z)-5-(2-methyl-5-phenylpent-1-en-4-yn-3-ylidene)non-1-en-3-ol (**4e**)

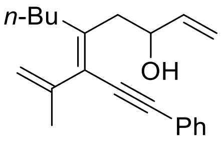

4e

82% isolated yield, colorless oil. ^{1}H NMR (400 MHz, $CDCl_3$) δ 7.44 – 7.40 (m, 2H), 7.31 – 7.27 (m, 3H), 5.98 (ddd, *J* = 16.8, 10.4, 6.0 Hz, 1H), 5.32 – 5.23 (m, 1H), 5.13 (d, *J* = 10.4 Hz, 1H), 5.08 – 5.03 (m, 1H), 4.89 – 4.84 (m, 1H), 4.48 – 4.42 (m, 1H), 2.77 (dd, *J* = 13.2, 8.4 Hz, 1H), 2.65 (dd, *J* = 13.2, 5.6 Hz, 1H), 2.39 – 2.23 (m, 2H), 1.99 – 1.93 (m, 3H), 1.78 (d, *J* = 4.5 Hz, 1H), 1.42 – 1.29 (m, 4H), 0.91 (t, *J* = 7.2 Hz, 3H); ^{13}C NMR (100 MHz, $CDCl_3$) δ 145.8, 142.5, 140.9, 131.3, 128.3, 127.9, 124.0, 123.7, 114.8, 114.5, 93.3, 88.7, 72.5, 41.5, 32.5, 31.0, 22.9, 22.8, 14.0; HRMS (ESI): calc. for $C_{21}H_{26}NaO$ $[M+Na]^+$: 317.1876; found: 317.1877.

Typical procedure for the formation of 5

Preparation of 4-cyclohexyl-2-methylene-3-(prop-1-en-2-yl)cyclopent-3-en-1-ol (**5a**):

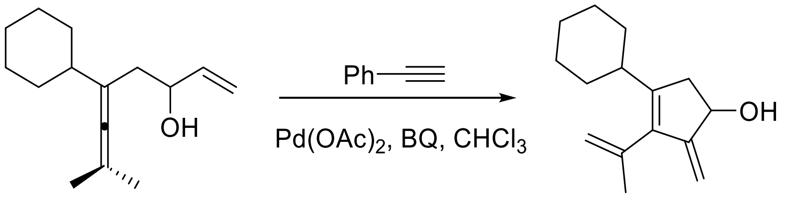

1a **5a**

To a solution of $Pd(OAc)_2$ (2.25 mg, 0.01 mmol) and BQ (23.8 mg, 0.22 mmol) in $CHCl_3$ (1.0 mL) was added enallene **1a** (44.0 mg, 0.2 mmol) and phenylacetylene (25.5 mg, 0.25 mmol). The reaction mixture was stirred at room temperature for 8 h and then the solvent was evaporated under vacuum. The residue was purified via column chromatography on silica gel (eluent: petroleum ether/ethyl acetate = 15/1) to afford **5a** (39.3 mg, 90%), colorless oil. ^{1}H NMR (400 MHz, $CDCl_3$) δ 5.18 – 5.10 (m, 1H), 4.99 – 4.92 (m, 1H), 4.89 – 4.81 (m, 1H), 4.77 – 4.58 (m, 2H), 2.75 (dd, *J* = 17.2, 7.2 Hz, 1H), 2.49 – 2.40 (m, 1H), 2.28 (dd, *J* = 17.2, 2.6 Hz, 1H), 1.89 – 1.83 (m, 3H), 1.76 – 1.51 (m, 6H), 1.39 – 1.28 (m, 4H); ^{13}C NMR (100 MHz, $CDCl_3$) δ 157.9, 149.2, 139.8, 138.3, 115.3, 102.8, 72.2, 40.0, 38.6, 31.5, 31.4, 26.2, 26.2, 26.0, 23.0; HRMS (ESI): calc. for $C_{15}H_{22}NaO$ $[M+Na]^+$: 241.1563; found: 241.1566.

The general method above was used for the preparation of the following compounds:

4-Methylene-5-(prop-1-en-2-yl)-[1,1'-bi(cyclopentan)]-5-en-3-ol (**5b**)

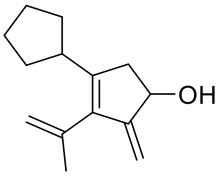

5b

87% isolated yield, colorless oil. ^{1}H NMR (400 MHz, $CDCl_3$) δ 5.18 – 5.10 (m, 1H), 4.99 – 4.91 (m, 1H), 4.85 – 4.82 (m, 1H), 4.75 – 4.64 (m, 2H), 2.93 – 2.83 (m, 1H), 2.76 (dd, *J* = 17.2, 7.2 Hz, 1H), 2.30 (dd, *J* = 17.2, 2.8 Hz, 1H), 1.87 – 1.82 (m, 3H), 1.71 – 1.63 (m, 5H), 1.59 – 1.55 (m, 1H), 1.49 – 1.38 (m, 2H); ^{13}C NMR (100 MHz, $CDCl_3$) δ 157.8, 147.6, 139.9, 139.3, 115.2, 102.7, 72.2, 39.6, 39.4, 31.5, 31.5, 25.9, 25.9, 22.9; HRMS (ESI): calc. for $C_{14}H_{20}NaO$ $[M+Na]^+$: 227.1406; found:

227.1400.

4-Cyclopropyl-2-methylene-3-(prop-1-en-2-yl)cyclopent-3-en-1-ol (**5c**)

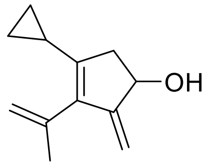

5c

84% isolated yield, colorless oil. ^{1}H NMR (400 MHz, $CDCl_3$) δ 5.23 – 5.19 (m, 1H), 4.94 – 4.81 (m, 3H), 4.69 – 4.60 (m, 1H), 2.44 (dd, *J* = 17.2, 7.2 Hz, 1H), 2.00 (dd, *J* = 17.2, 2.6 Hz, 1H), 1.93 – 1.88 (m, 3H), 1.84 – 1.77 (m, 1H), 1.64 (d, *J* = 5.8 Hz, 1H), 0.75 – 0.61 (m, 4H); ^{13}C NMR (100 MHz, $CDCl_3$) δ 157.7, 145.3, 139.7, 139.4, 115.7, 101.8, 71.9, 39.1, 22.5, 11.7, 5.6, 5.5; HRMS (ESI): calc. for $C_{12}H_{16}NaO$ $[M+Na]^+$: 199.1093; found: 199.1088.

2-Methylene-3-(prop-1-en-2-yl)-4-propylcyclopent-3-en-1-ol (**5d**)

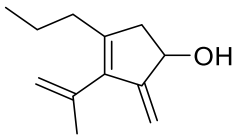

5d

89% isolated yield, colorless oil. ^{1}H NMR (400 MHz, $CDCl_3$) δ 5.18 – 5.10 (m, 1H), 5.00 – 4.95 (m, 1H), 4.89 – 4.81 (m, 1H), 4.75 – 4.65 (m, 2H), 2.74 (dd, *J* = 17.4, 7.0 Hz, 1H), 2.35 – 2.28 (m, 1H), 2.20 – 2.14 (m, 2H), 1.86 – 1.83 (m, 3H), 1.65 (d, *J* = 6.0 Hz, 1H), 1.48 – 1.41 (m, 2H), 0.89 (t, *J* = 7.4 Hz, 3H); ^{13}C NMR (100 MHz, $CDCl_3$) δ 157.8, 144.7, 139.9, 139.6, 115.5, 102.9, 72.3, 43.2, 31.6, 22.7, 21.1, 14.0; HRMS (ESI): calc. for $C_{12}H_{18}NaO$ $[M+Na]^+$: 201.1250; found: 201.1244.

4-Butyl-2-methylene-3-(prop-1-en-2-yl)cyclopent-3-en-1-ol (**5e**)

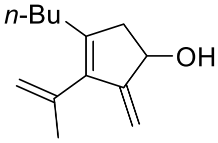

5e

87% isolated yield, colorless oil. ^{1}H NMR (400 MHz, $CDCl_3$) δ 5.18 – 5.12 (m, 1H), 5.00 – 4.95 (m, 1H), 4.90 – 4.85 (m, 1H), 4.78 – 4.62 (m, 2H), 2.75 (dd, *J* = 17.3, 7.1 Hz, 1H), 2.36 – 2.26 (m, 1H), 2.24 – 2.14 (m, 2H), 1.87 – 1.82 (m, 3H), 1.65 (d, *J* =

5.6 Hz, 1H), 1.42 – 1.26 (m, 4H), 0.89 (t, *J* = 7.2 Hz, 3H); ^{13}C NMR (100 MHz, $CDCl_3$) δ 157.8, 144.9, 139.7, 139.6, 115.4, 102.9, 72.3, 43.2, 30.2, 29.3, 22.7, 22.7, 13.9; HRMS (ESI): calc. for $C_{13}H_{20}NaO$ $[M+Na]^+$: 215.1406; found: 215.1411.

Recycling experiments and kinetic studies

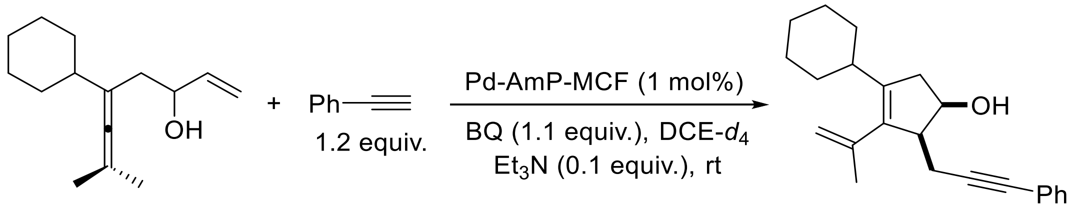

To a mixture of Pd-AmP-MCF (4.1 mg, 0.002 mmol), Et_3N (2.8 μL mg, 0.02 mmol) and BQ (23.8 mg, 0.22 mmol) in DCE-d_4 (1.0 mL) was added enallene **1a** (44.0 mg, 0.2 mmol), phenylacetylene (25.5 mg, 0.25 mmol) and anisole (22 μL, 0.2 mmol). The reaction was conducted at room temperature in an NMR tube and the yields at different time were determined by ^{1}H NMR measurements. After completed, the reaction mixture was decanted to a centrifuge tube and centrifuged for 2 min at 7000 rpm. The supernatant was removed, and the remaining catalyst (Pd-AmP-MCF) was washed by DCE (2 × 5 mL) and then dried under vacuum before being used in the next run.

XPS spectra of Pd-AmP-MCF before reaction, after run 1 and after run 7:

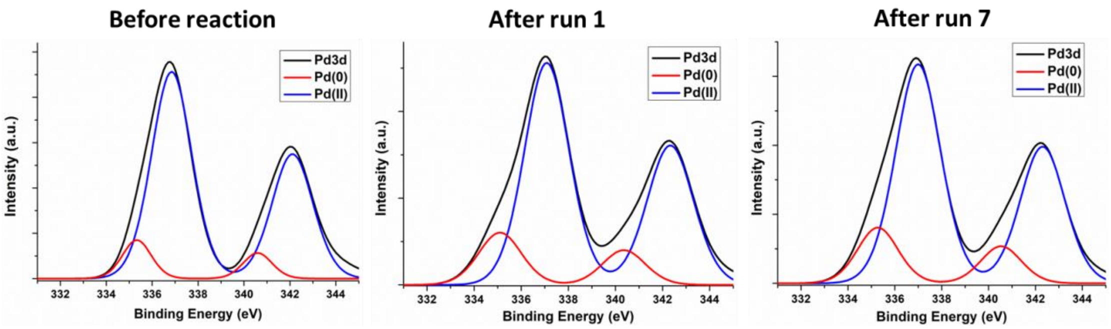

Hot filtration test

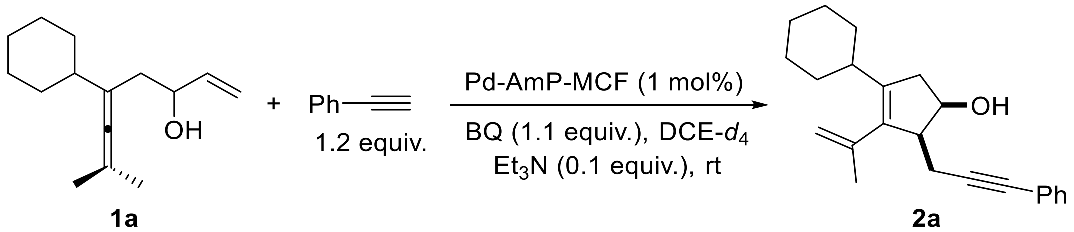

To a mixture of Pd-AmP-MCF (4.1 mg, 0.002 mmol), Et_3N (2.8 μL mg, 0.02 mmol) and BQ (23.8 mg, 0.22 mmol) in DCE-d_4 (1.0 mL) was added enallene **1a** (44.0 mg, 0.2 mmol), phenylacetylene (25.5 mg, 0.25 mmol), and anisole (22 μL, 0.2 mmol). The reaction was conducted at room temperature for 2 h and then centrifuged for 2 min at 7000 rpm. The yield of **2a** was determined to be 37% by ^{1}H NMR measurement. The supernatant was transferred to another clean reaction tube and stirred at room temperature for 8 h. Analysis of the reaction mixture by ^{1}H NMR showed that the yield of **2a** was unchanged (still 37% yield).

Asymmetric syntheses of 2, 4 and 5

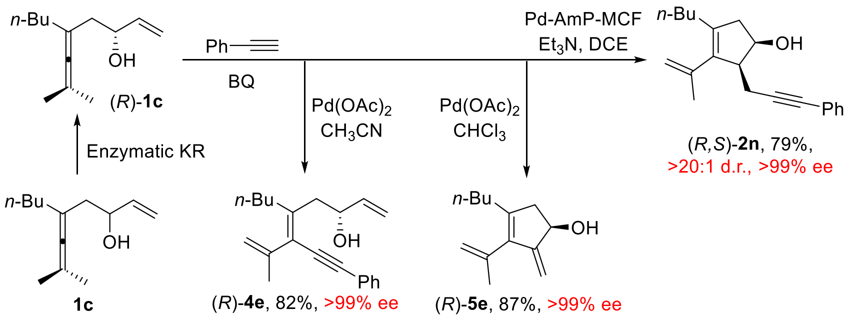

Enzymatic KR: To a solution of **1c** (388 mg, 2.0 mmol) in toluene (1.0 mL) was added isopropenyl acetate (300 mg, 3.0 mmol), Na_2CO_3 (210 mg, 2.0 mmol) and *Candida antarctica* lipase B (CalB, 10.0 mg). The mixture was stirred at room temperature for 8 h and then purified via column chromatography on silica gel (eluent: petroleum ether/ethyl acetate = 20/1) to afford (*R*)**-1c** (180 mg, 46%).

Pd-AmP-MCF catalyzed oxidative domino reaction: To a mixture of Pd-AmP-MCF (4.1 mg, 0.002 mmol), Et_3N (2.8 µL mg, 0.02 mmol) and BQ (23.8 mg, 0.22 mmol) in DCE (1.0 mL) was added (*R*)**-1c** (38.8 mg, 0.2 mmol) and phenylacetylene (25.5 mg, 0.25 mmol). The reaction mixture was stirred at room temperature for 8 h and then the solvent was evaporated under vacuum. The residue was purified via column chromatography on silica gel (eluent: petroleum ether/ethyl acetate = 15/1) to afford (*R*,*S*)**-2n** (46.3 mg, 79%, > 20:1 d.r., > 99% ee).

HPLC for **2n**: Chiralpak AD-H column, *i*-PrOH/*i*-hexane (5/95), flow rate 1.0 mL/min, t_R: 14.9 min for (*S*,*R*)-isomer and 16.3 min for (*R*,*S*)-isomer.

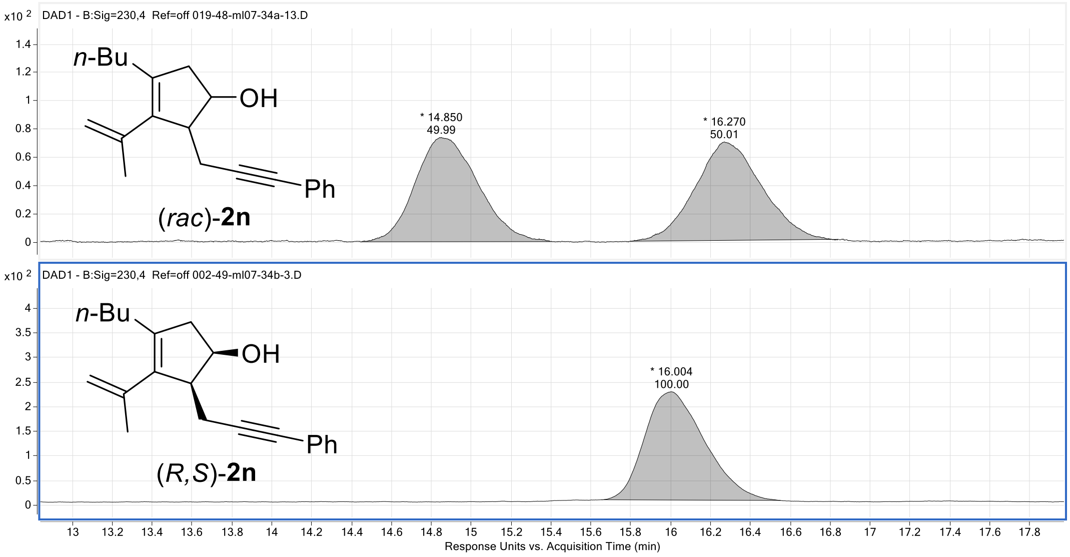

Pd(OAc)$_2$ catalyzed oxidative alkynylation reaction: To a solution of Pd(OAc)$_2$ (2.25 mg, 0.01 mmol) and BQ (23.8 mg, 0.22 mmol) in CH_3CN (1.0 mL) was added (*R*)-**1c** (38.8 mg, 0.2 mmol) and phenylacetylene (25.5 mg, 0.25 mmol). The reaction mixture was stirred at room temperature for 8 h and then the solvent was evaporated under vacuum. The residue was purified via column chromatography on silica gel (eluent: petroleum ether/ethyl acetate = 15/1) to afford (*R*)-**4e** (48.0 mg, 82%, > 99% ee).

HPLC for **4e**: Chiralpak IB-H column, *i*-PrOH/*i*-hexane (5/95), flow rate 1.0 mL/min, t_R: 6.8 min for (*S*)-isomer and 7.2 min for (*R*)-isomer.

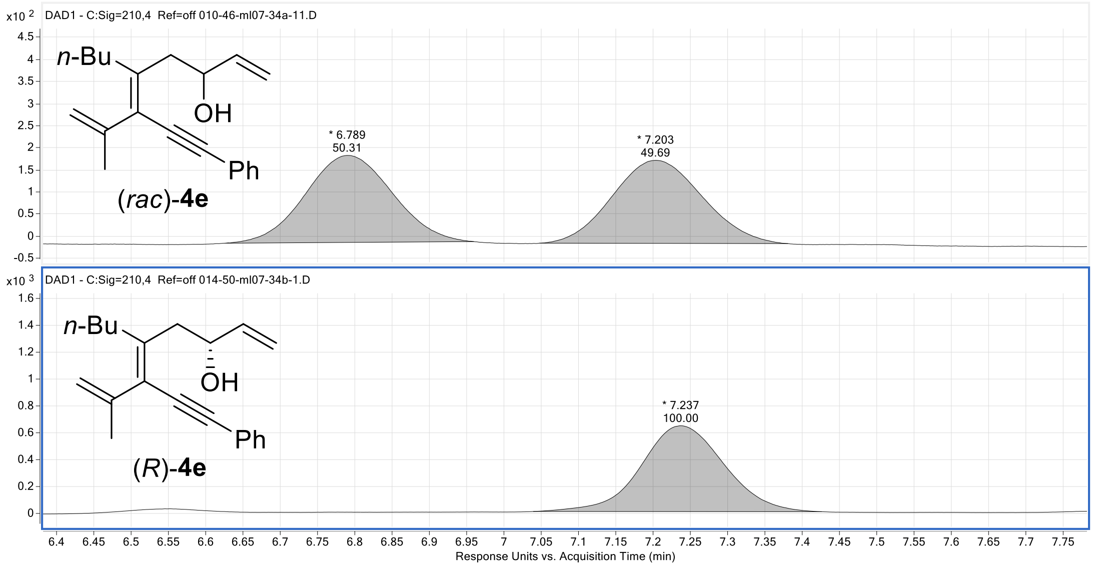

Pd(OAc)$_2$ catalyzed oxidative carbocyclization reaction: To a solution of Pd(OAc)$_2$ (2.25 mg, 0.01 mmol) and BQ (23.8 mg, 0.22 mmol) in $CHCl_3$ (1.0 mL) was added (*R*)-**1c** (38.8 mg, 0.2 mmol). The reaction mixture was stirred at room temperature for 8 h and then the solvent was evaporated under vacuum. The residue was purified via column chromatography on silica gel (eluent: petroleum ether/ethyl acetate = 15/1) to afford (*R*)-**5e** (33.2 mg, 87%, > 99% ee).

HPLC for **5e**: Chiralpak IB-H column, *i*-PrOH/*i*-hexane (5/95), flow rate 1.0 mL/min, t_R: 4.4 min for (*R*)-isomer and 4.5 min for (*S*)-isomer.

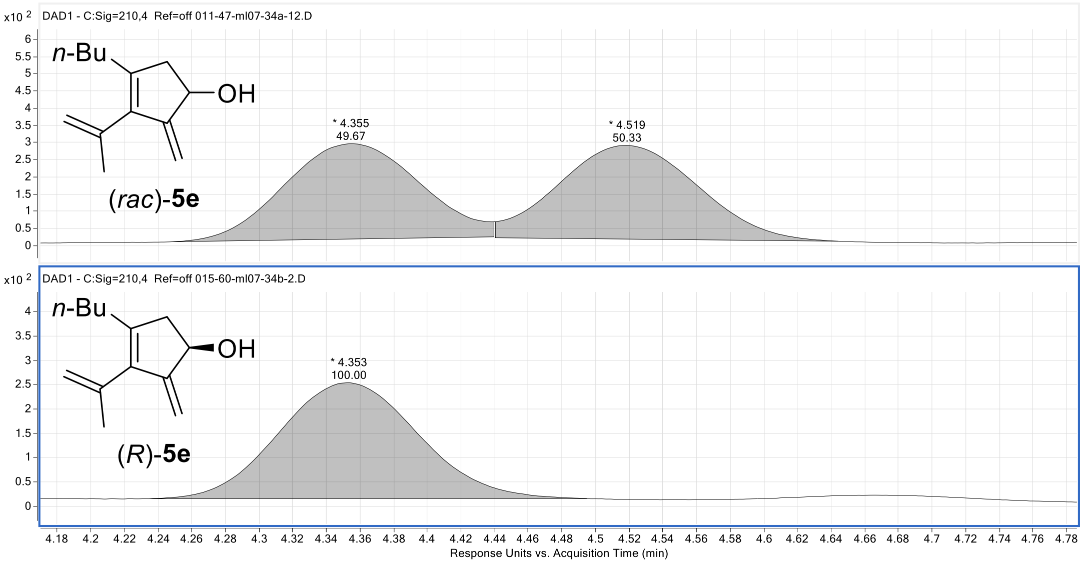
DAD1 - C:Sig=210,4 Ref=off 011-47-ml07-34a-12.D
n-Bu
OH
(rac)-5e
* 4.355
49.67
* 4.519
50.33
DAD1 - C:Sig=210,4 Ref=off 015-60-ml07-34b-2.D
(R)-5e
* 4.353
100.00
Response Units vs. Acquisition Time (min)

Typical procedure for the formation of 6

Preparation of 4-phenyl-3-(prop-1-en-2-yl)-2-((4,4,5,5-tetramethyl-1,3,2-dioxaborolan-2-yl)methyl)cyclopent-3-en-1-ol (**6**):

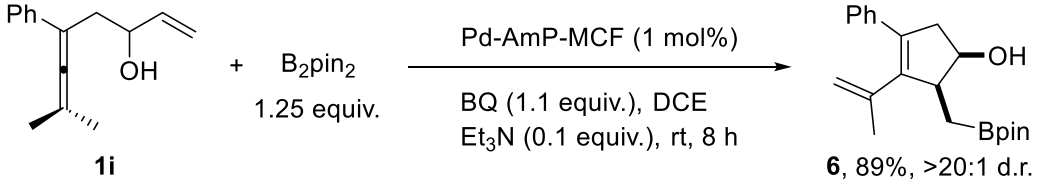

To a solution of Pd-AmP-MCF (4.1 mg, 0.002 mmol), Et_3N (2.8 μL mg, 0.02 mmol) and BQ (23.8 mg, 0.22 mmol) in DCE (1.0 mL) was added enallene **1i** (42.8 mg, 0.2 mmol) and B_2pin_2 (63.5 mg, 0.25 mmol). The reaction mixture was stirred at room temperature for 8 h and then the solvent was evaporated under vacuum. The residue was purified via column chromatography on silica gel (eluent: petroleum ether/ethyl acetate = 15/1) to afford **6** (60.5 mg, 89%), colorless oil. ^{1}H NMR (400 MHz, $CDCl_3$) δ 7.41 – 7.36 (m, 2H), 7.29 – 7.24 (m, 2H), 7.21 – 7.15 (m, 1H), 5.05 – 5.00 (m, 1H), 4.80 – 4.71 (m, 1H), 4.15 (dt, *J* = 7.6, 5.2 Hz, 1H), 3.34 (s, 1H), 3.19 (ddd, *J* = 16.4, 7.6, 1.6 Hz, 1H), 2.96 – 2.90 (m, 1H), 2.64 (ddd, *J* = 16.4, 5.2, 1.6 Hz, 1H), 1.76 – 1.70 (m, 3H), 1.30 – 1.25 (m, 13H), 0.69 (dd, *J* = 16.4, 12.0 Hz, 1H); ^{13}C NMR (100 MHz, $CDCl_3$) δ 143.0, 141.5, 137.7, 131.5, 127.9, 127.5, 126.6, 115.4, 83.6, 78.2, 53.8, 43.9, 24.9, 24.8, 22.5; HRMS (ESI): calc. for $C_{21}H_{29}BNaO_3$ [M+Na]$^+$: 363.2102; found: 363.2109.

References

[1] M.-B. Li, D. Posevins, A. Geoffroy, C. Zhu, J.-E. Bäckvall, *Angew. Chem. Int. Ed.* **2020**, *59*, 1992-1996; *Angew. Chem.* **2020**, *132*, 2008-2012.

[2] M.-B. Li, Y. Yang, A. A. Rafi, M. Oschmann, E. S. Grape, A. K. Inge, A. Córdova, J.-E. Bäckvall, *Angew. Chem. Int. Ed.* **2020**, *59*, 10391-10395; *Angew. Chem.* **2020**, *132*, 10477-10481.

[3] D. Posevins, M.-B. Li, E. S. Grape, A. K. Inge, Y. Qiu, J.-E. Bäckvall, *Org. Lett.* **2020**, *22*, 417-421.

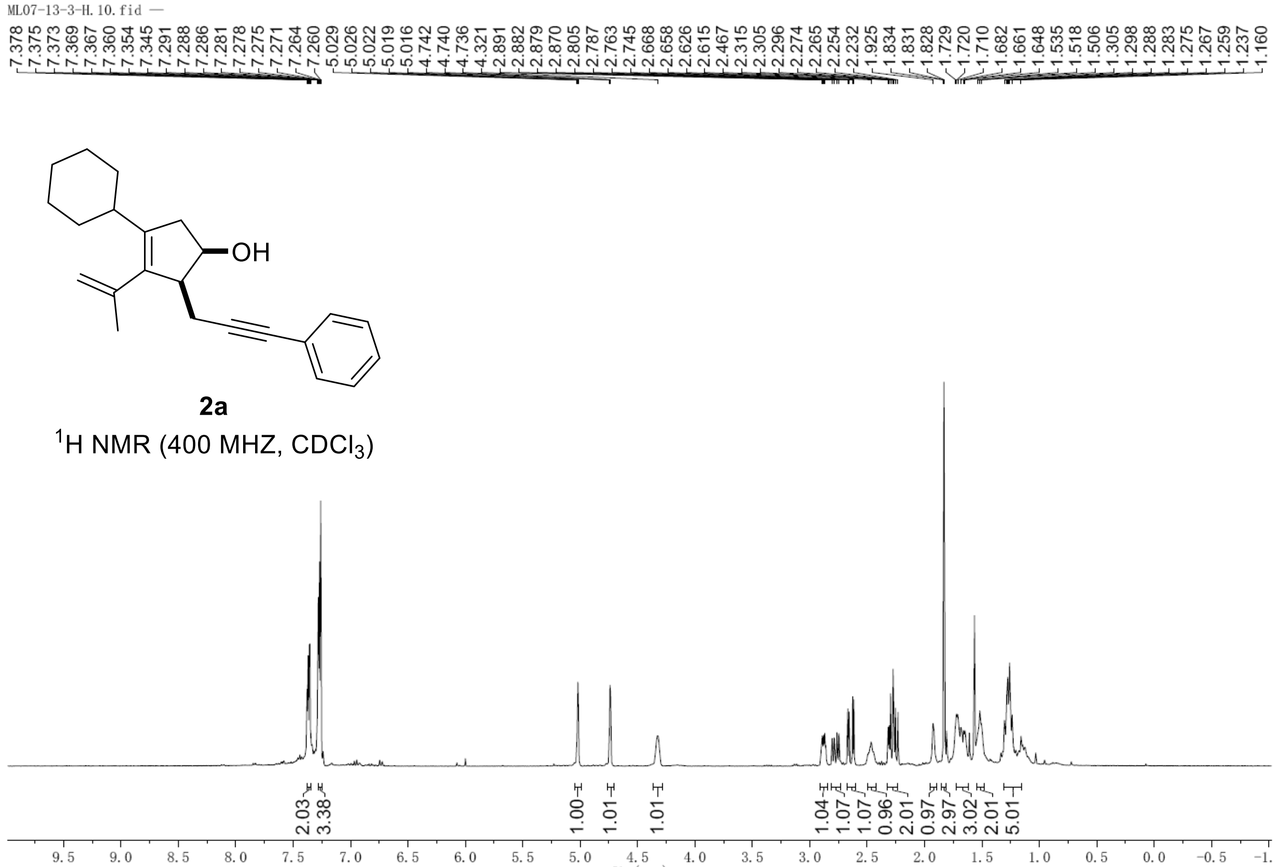
ML07-13-3-H.10.fid —
7.378 7.375 7.373 7.369 7.367 7.360 7.354 7.345 7.291 7.288 7.286 7.281 7.278 7.275 7.271 7.264 7.260 5.029 5.026 5.022 5.019 5.016 4.742 4.740 4.736 4.321 2.891 2.882 2.879 2.870 2.805 2.787 2.763 2.745 2.668 2.658 2.626 2.615 2.467 2.315 2.305 2.296 2.274 2.265 2.254 2.232 1.925 1.834 1.831 1.828 1.729 1.720 1.710 1.682 1.661 1.648 1.535 1.518 1.506 1.305 1.298 1.288 1.283 1.275 1.267 1.259 1.237 1.160
OH
2a
^{1}H NMR (400 MHZ, CDCl$_3$)
2.03 3.38 1.00 1.01 1.01 1.04 1.07 1.07 0.96 2.01 0.97 2.97 3.02 2.01 5.01
9.5 9.0 8.5 8.0 7.5 7.0 6.5 6.0 5.5 5.0 4.5 4.0 3.5 3.0 2.5 2.0 1.5 1.0 0.5 0.0 −0.5 −1.
f1 (ppm)

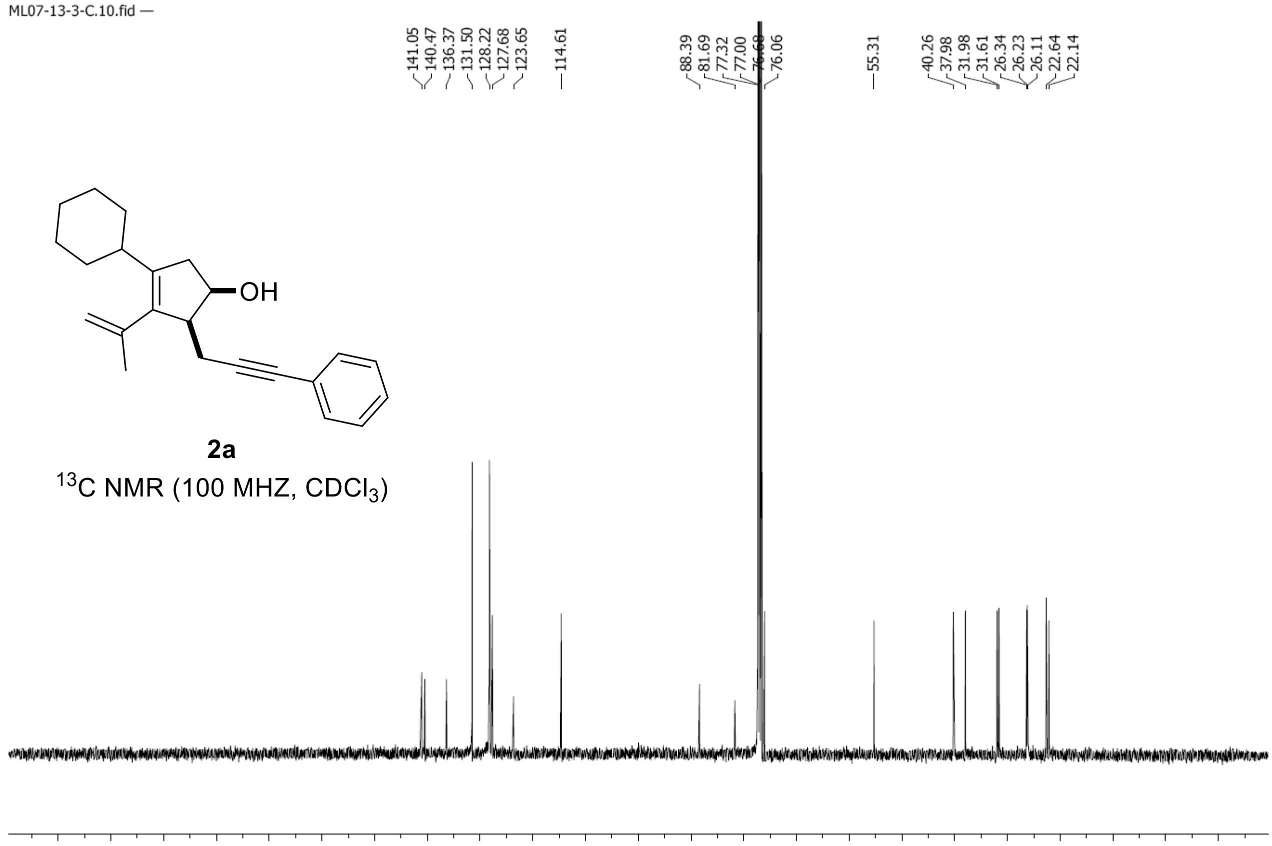
ML07-13-3-C.10.fid —
141.05 140.47 136.37 131.50 128.22 127.68 123.65 114.61 88.39 81.69 77.32 77.00 76.68 76.06 55.31 40.26 37.98 31.98 31.61 26.34 26.23 26.11 22.64 22.14
OH
2a
^{13}C NMR (100 MHZ, CDCl$_3$)
210 200 190 180 170 160 150 140 130 120 110 100 90 80 70 60 50 40 30 20 10 0 -10
f1 (ppm)

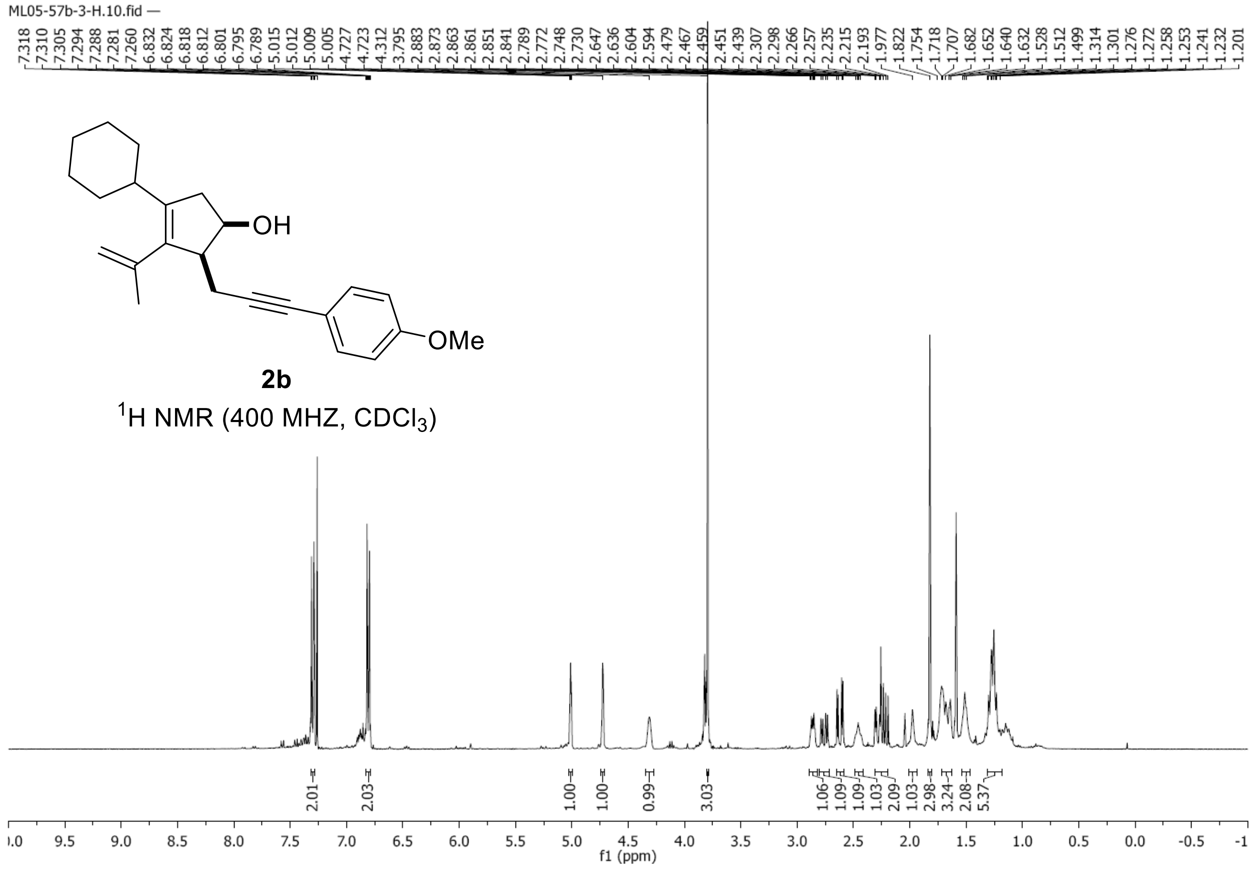
ML05-57b-3-H.10.fid
7.318 7.310 7.305 7.294 7.288 7.281 7.260 6.832 6.824 6.818 6.812 6.801 6.795 6.789 5.015 5.012 5.009 5.005 4.727 4.723 4.312 3.795 2.883 2.873 2.863 2.861 2.851 2.841 2.789 2.772 2.748 2.730 2.647 2.636 2.604 2.594 2.479 2.467 2.459 2.451 2.439 2.307 2.298 2.266 2.257 2.235 2.215 2.193 1.977 1.822 1.754 1.718 1.707 1.682 1.652 1.640 1.632 1.528 1.512 1.499 1.314 1.301 1.276 1.272 1.258 1.253 1.241 1.232 1.201
OH
OMe
2b
1H NMR (400 MHZ, CDCl3)
2.01 2.03 1.00 1.00 0.99 3.03 1.06 1.09 1.09 1.03 2.09 1.03 2.98 3.24 2.08 5.37
f1 (ppm)

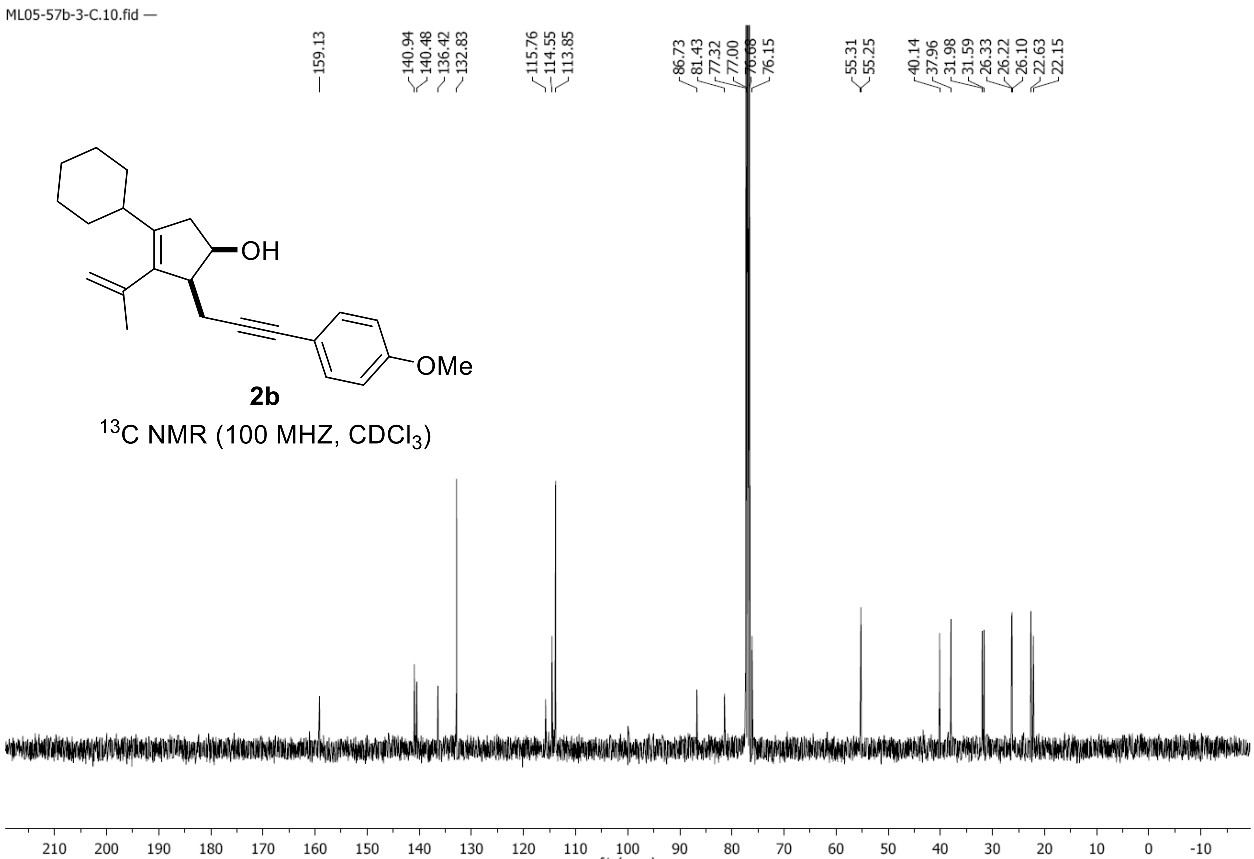
ML05-57b-3-C.10.fid
159.13
140.94 140.48 136.42 132.83
115.76 114.55 113.85
86.73 81.43 77.32 77.00 76.68 76.15
55.31 55.25
40.14 37.96 31.98 31.59 26.33 26.22 26.10 22.63 22.15
OH
OMe
2b
13C NMR (100 MHZ, CDCl3)
f1 (ppm)

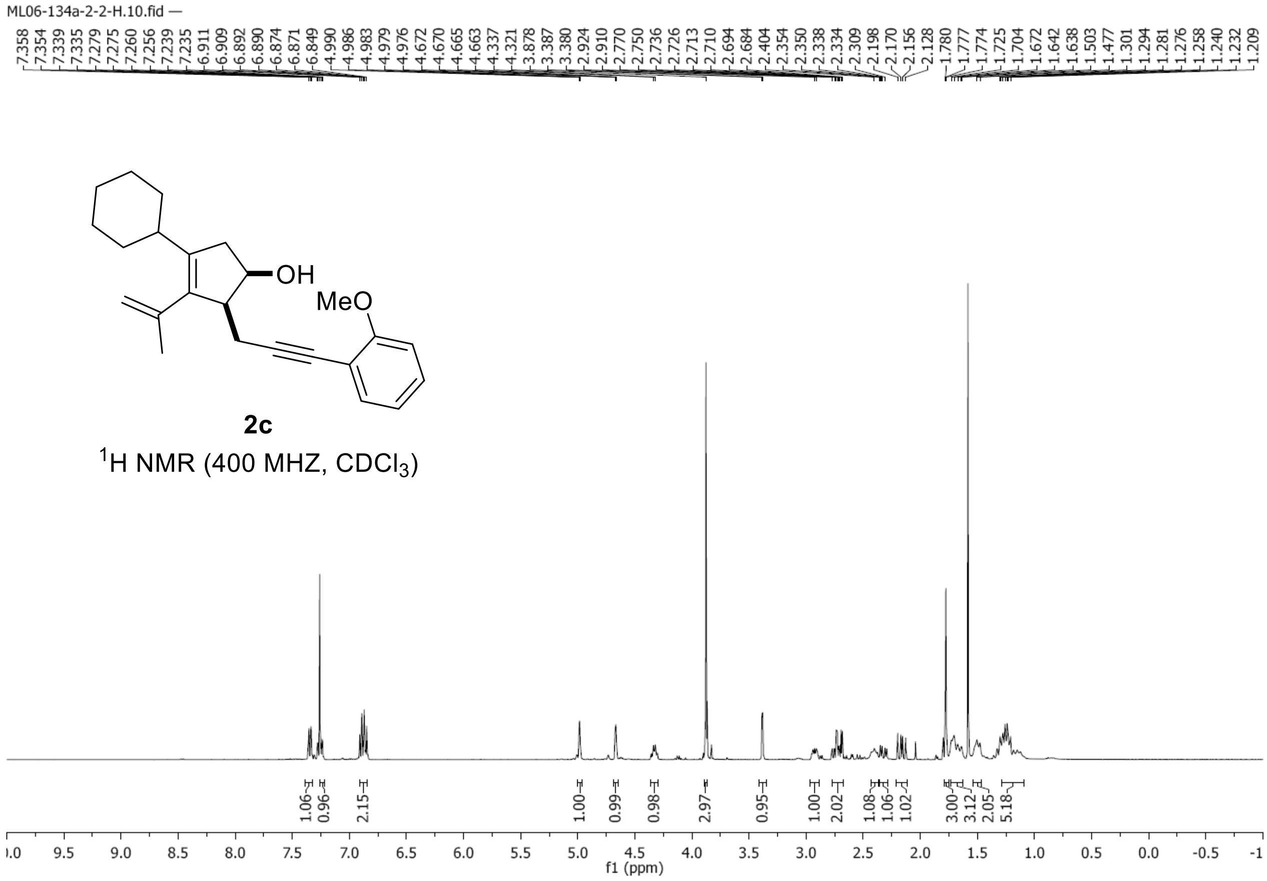

ML06-134a-2-2-H.10.fid
7.358 7.354 7.339 7.335 7.279 7.275 7.260 7.256 7.239 7.235 6.911 6.909 6.892 6.890 6.874 6.871 6.849 4.990 4.986 4.983 4.979 4.976 4.672 4.670 4.665 4.663 4.337 4.321 3.878 3.387 3.380 2.924 2.910 2.770 2.750 2.736 2.726 2.713 2.710 2.694 2.684 2.404 2.354 2.350 2.338 2.334 2.309 2.198 2.170 2.156 2.128 1.780 1.777 1.774 1.725 1.704 1.672 1.642 1.638 1.503 1.477 1.301 1.294 1.281 1.276 1.258 1.240 1.232 1.209
OH
MeO
2c
^{1}H NMR (400 MHZ, CDCl$_3$)
1.06 0.96 2.15 1.00 0.99 0.98 2.97 0.95 1.00 2.02 1.08 1.06 1.02 3.00 3.12 2.05 5.18
f1 (ppm)

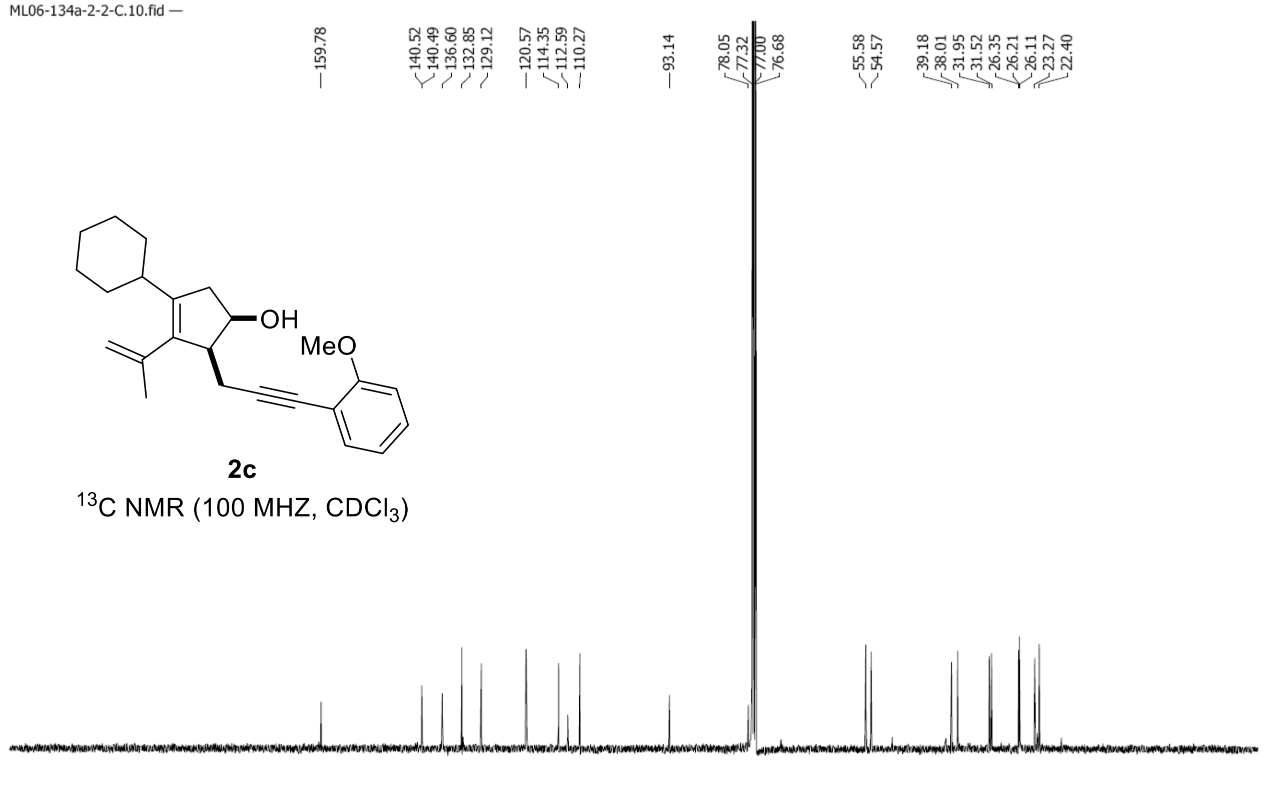

ML06-134a-2-2-C.10.fid
159.78 140.52 140.49 136.60 132.85 129.12 120.57 114.35 112.59 110.27 93.14 78.05 77.32 77.00 76.68 55.58 54.57 39.18 38.01 31.95 31.52 26.35 26.21 26.11 23.27 22.40
OH
MeO
2c
^{13}C NMR (100 MHZ, CDCl$_3$)
f1 (ppm)

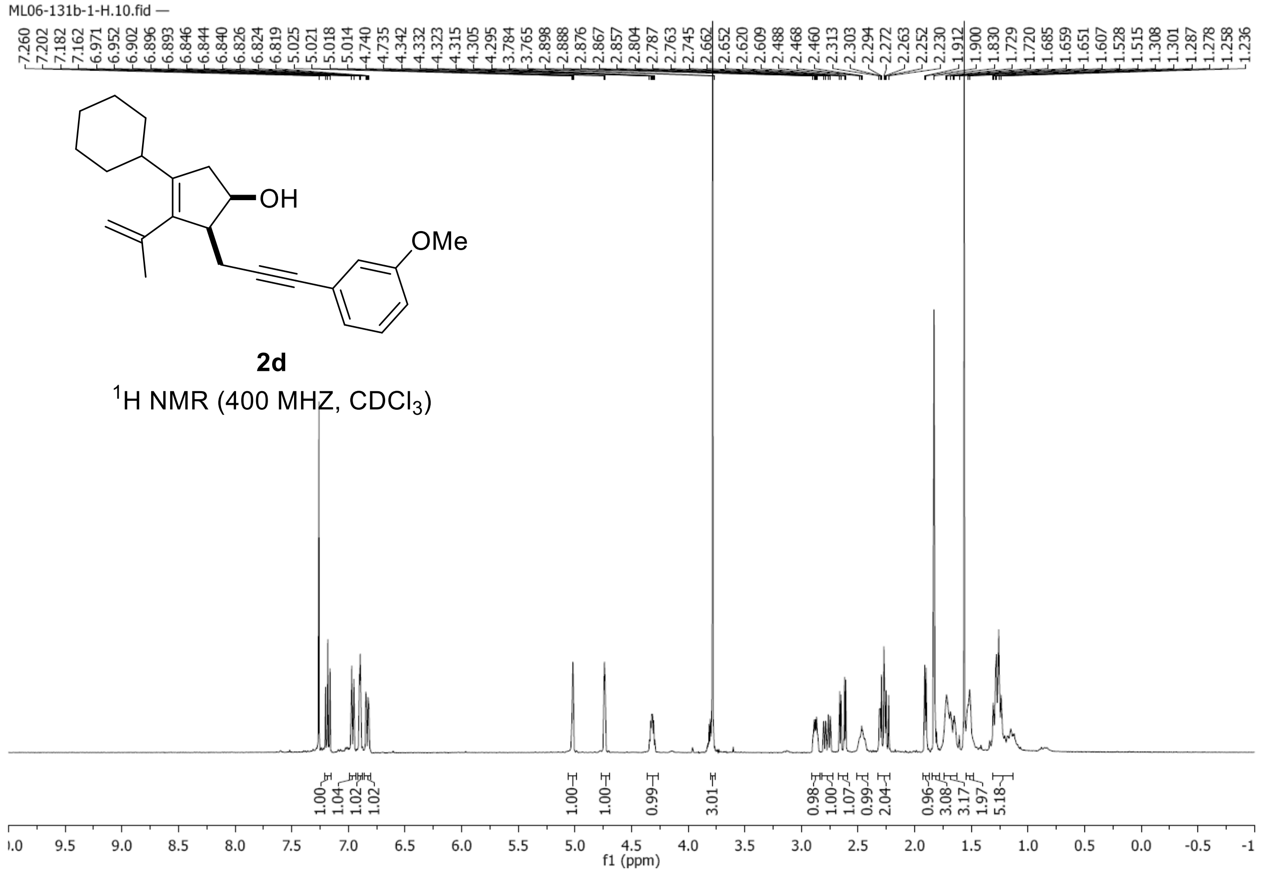
ML06-131b-1-H.10.fid
OH
OMe
2d
^{1}H NMR (400 MHZ, CDCl$_3$)
f1 (ppm)

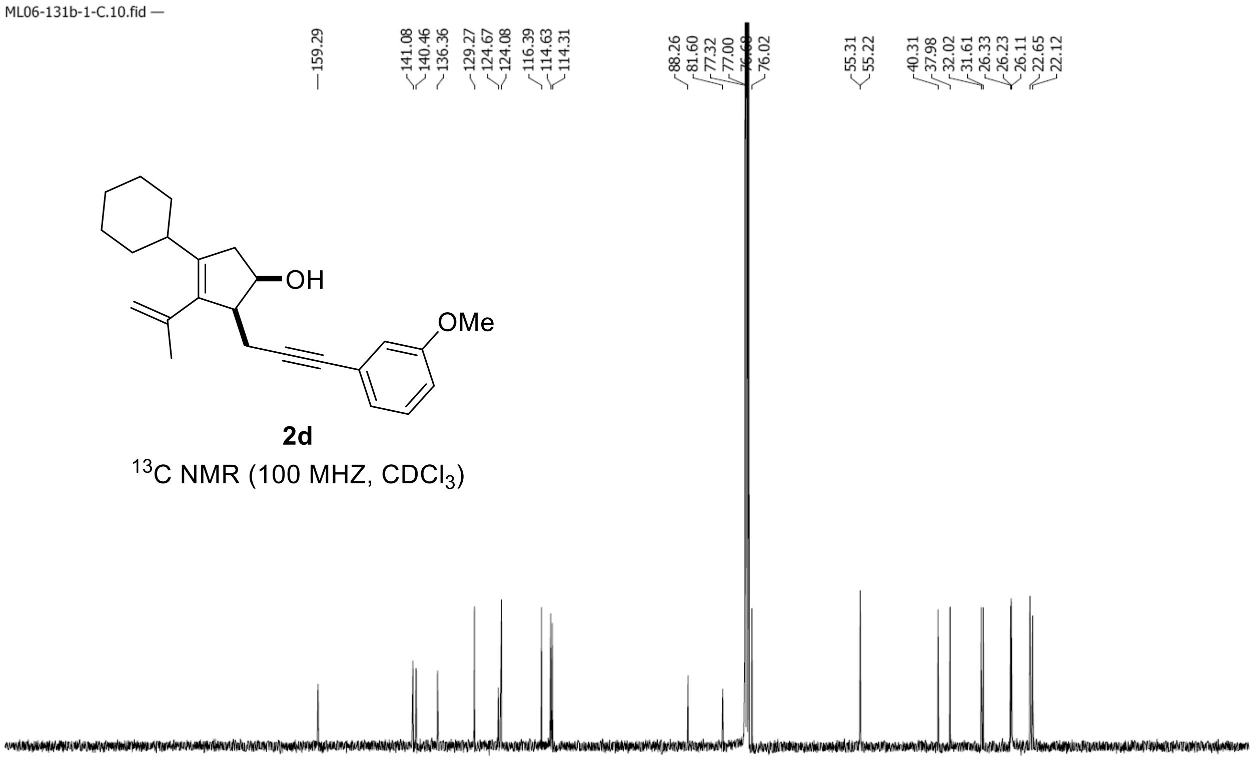
ML06-131b-1-C.10.fid
OH
OMe
2d
^{13}C NMR (100 MHZ, CDCl$_3$)
f1 (ppm)

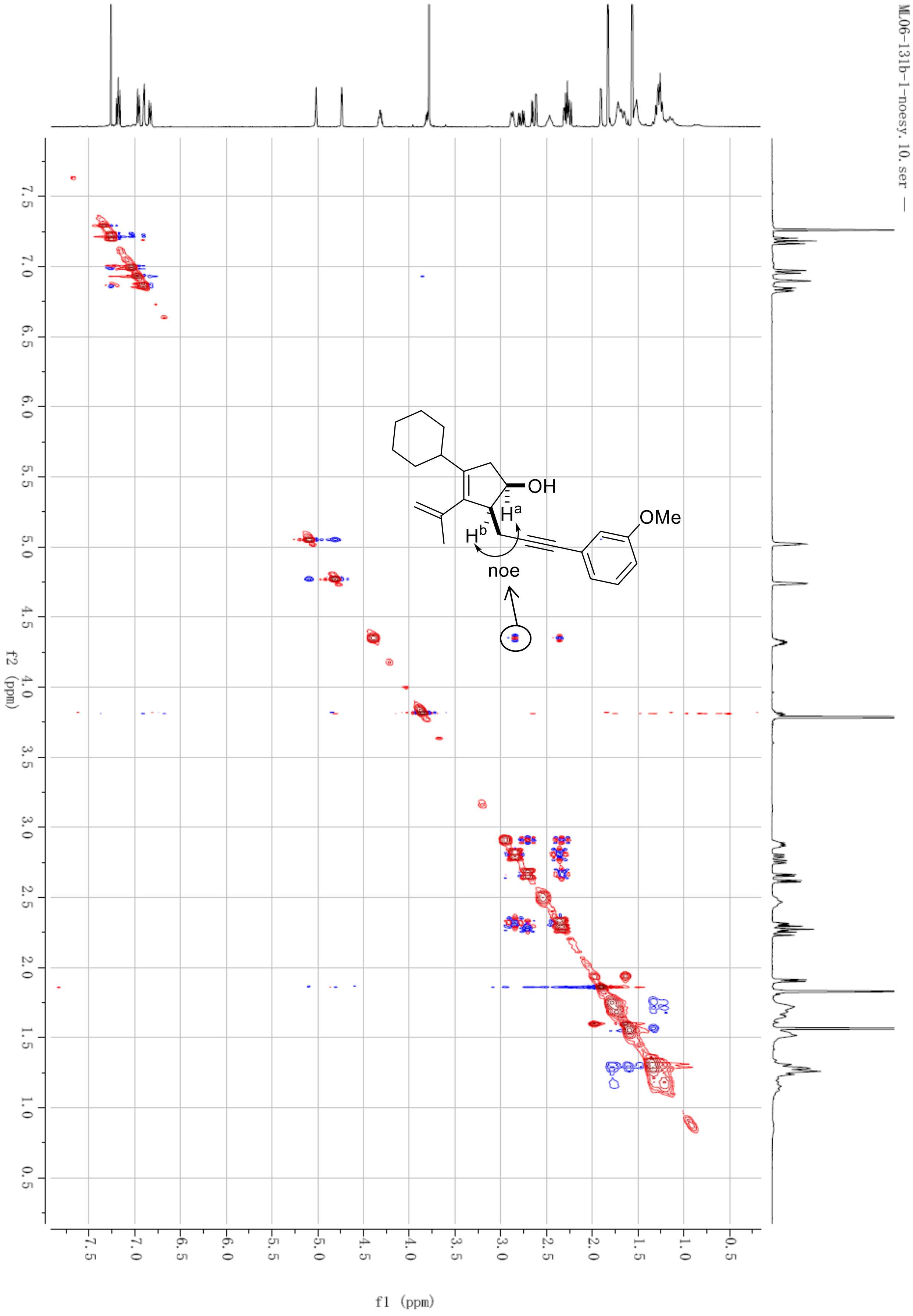

ML06-131b-1-noesy.10.ser
OH
OMe
H^a
H^b
noe
f1 (ppm)
f2 (ppm)

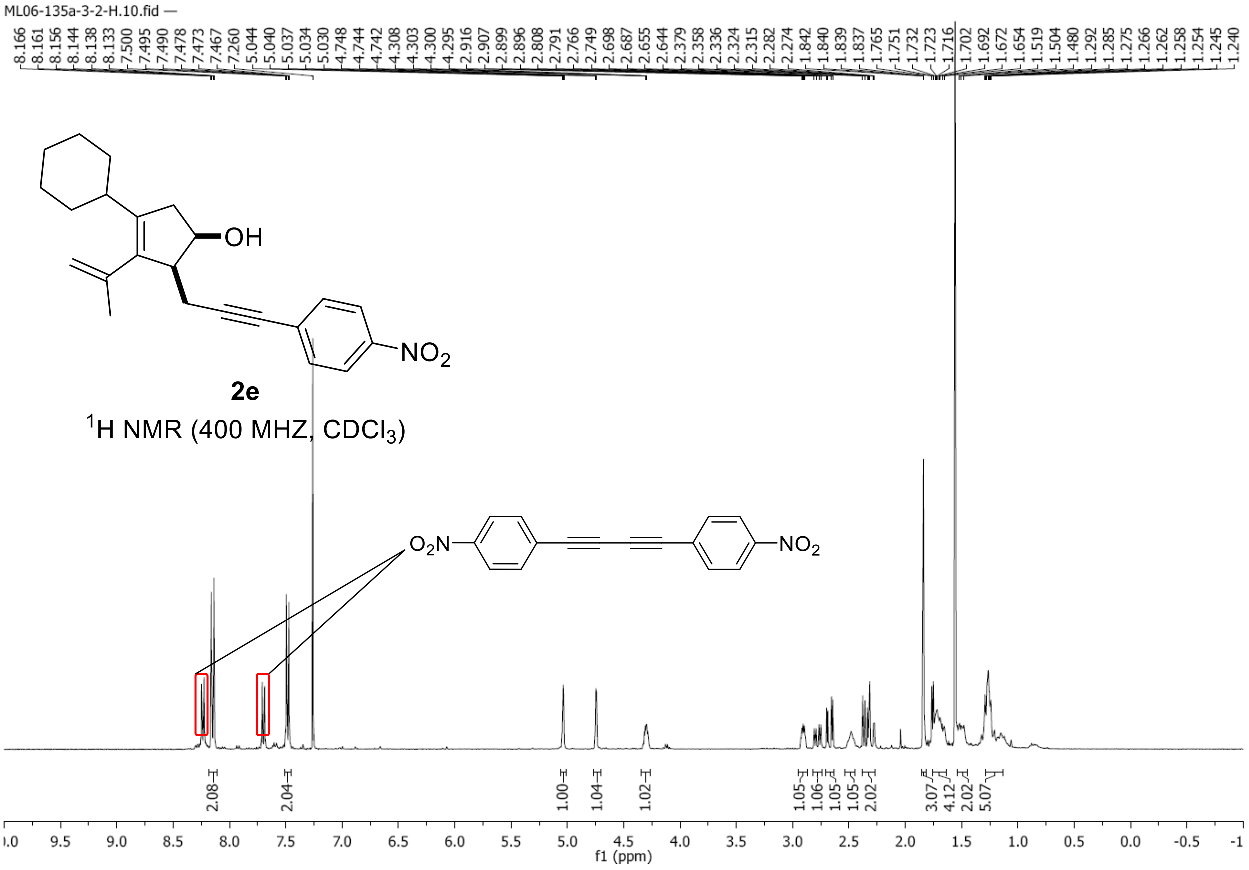
ML06-135a-3-2-H.10.fid —
8.166 8.161 8.156 8.144 8.138 8.133 7.500 7.495 7.490 7.478 7.473 7.467 7.260 5.044 5.040 5.037 5.034 5.030 4.748 4.744 4.742 4.308 4.303 4.300 4.295 2.916 2.907 2.899 2.896 2.808 2.791 2.766 2.749 2.698 2.687 2.655 2.644 2.379 2.358 2.336 2.324 2.315 2.282 2.274 1.842 1.840 1.839 1.837 1.765 1.751 1.732 1.723 1.716 1.702 1.692 1.672 1.654 1.519 1.504 1.480 1.292 1.285 1.275 1.266 1.262 1.258 1.254 1.245 1.240
OH
NO_2
2e
^1H NMR (400 MHZ, $CDCl_3$)
O_2N
NO_2
2.08
2.04
1.00
1.04
1.02
1.05
1.06
1.05
1.05
2.02
3.07
4.12
2.02
5.07
f1 (ppm)

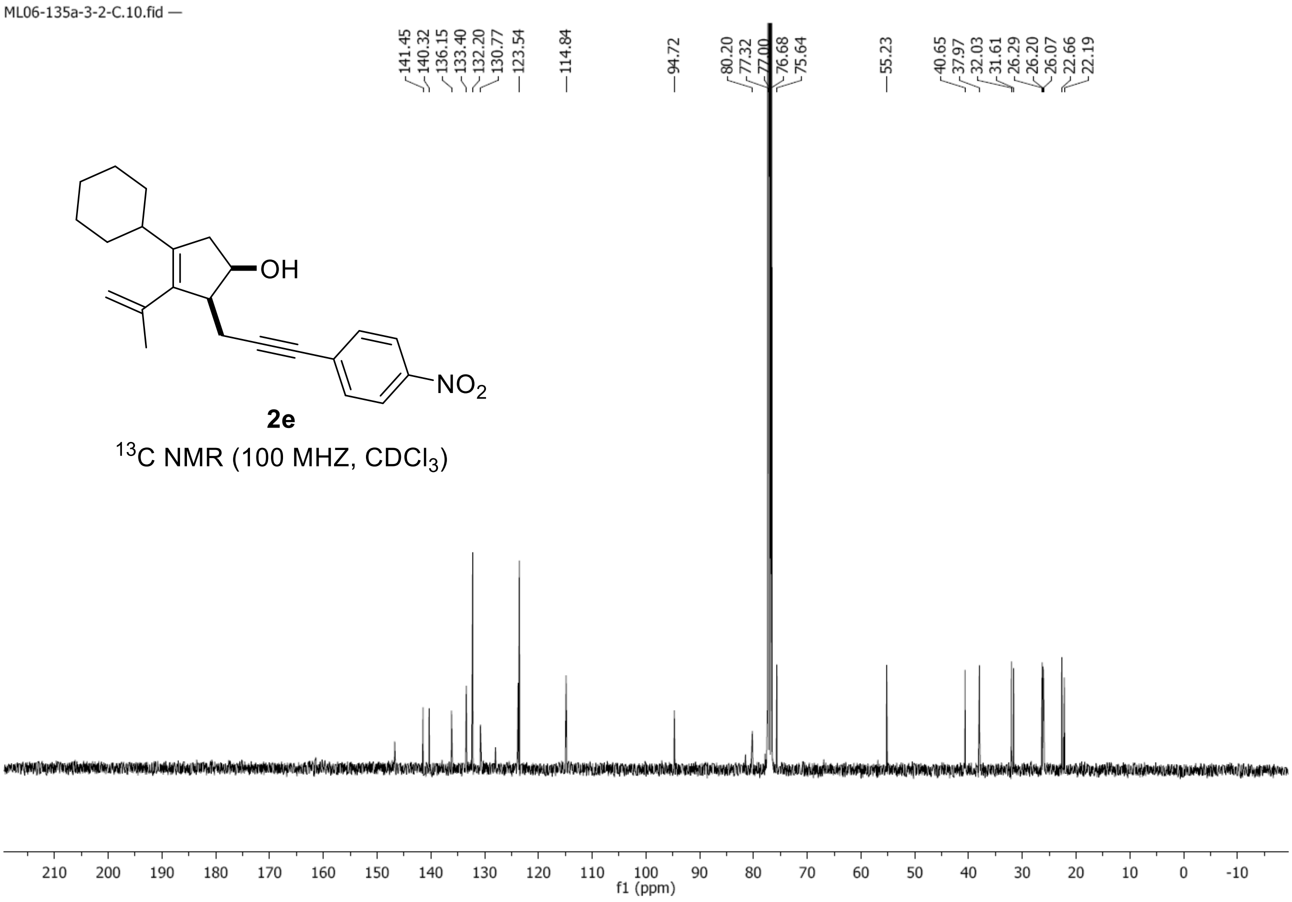
ML06-135a-3-2-C.10.fid —
141.45 140.32 136.15 133.40 132.20 130.77 123.54
114.84
94.72
80.20 77.32 77.00 76.68 75.64
55.23
40.65 37.97 32.03 31.61 26.29 26.20 26.07 22.66 22.19
OH
NO_2
2e
^{13}C NMR (100 MHZ, $CDCl_3$)
f1 (ppm)

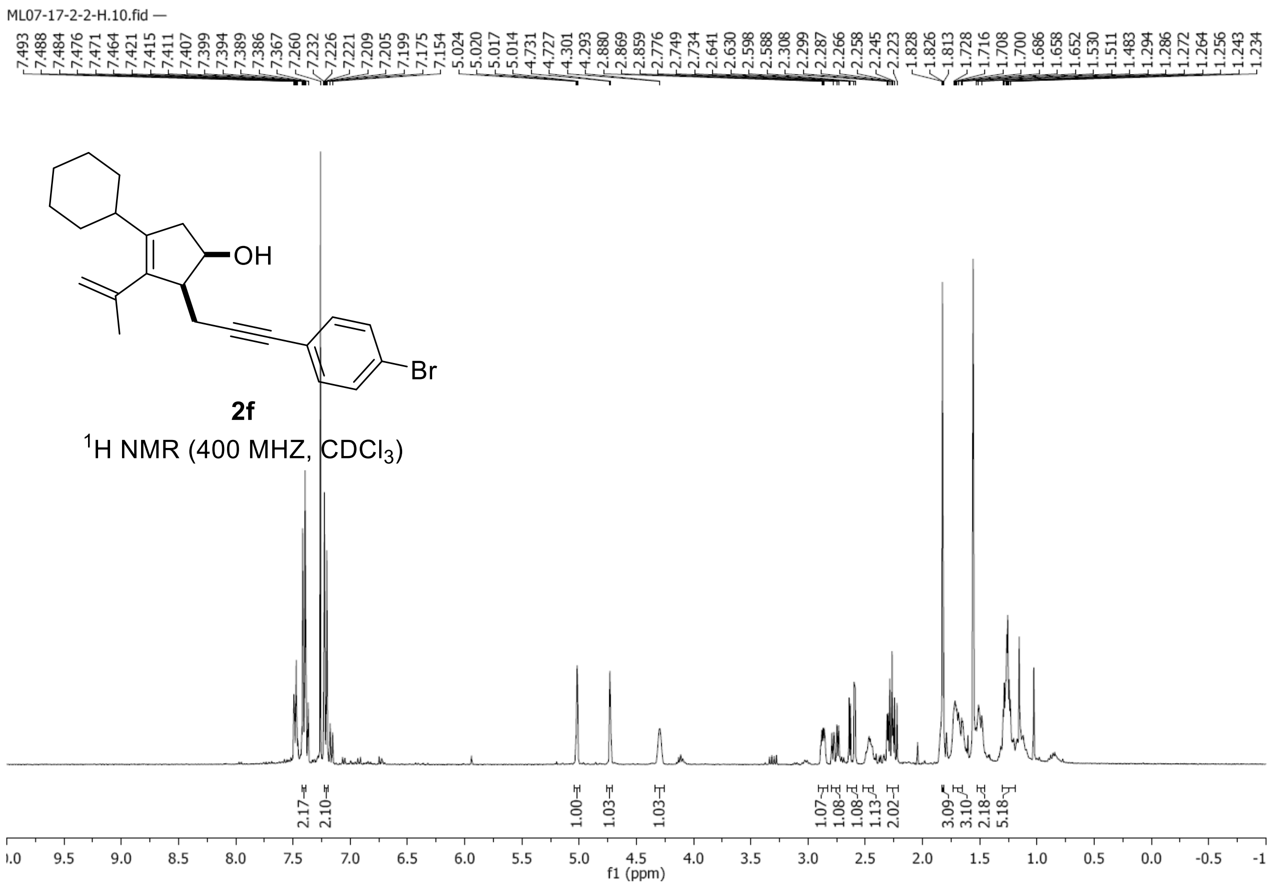
ML07-17-2-2-H.10.fid —
OH
Br
2f
1H NMR (400 MHZ, CDCl3)
f1 (ppm)

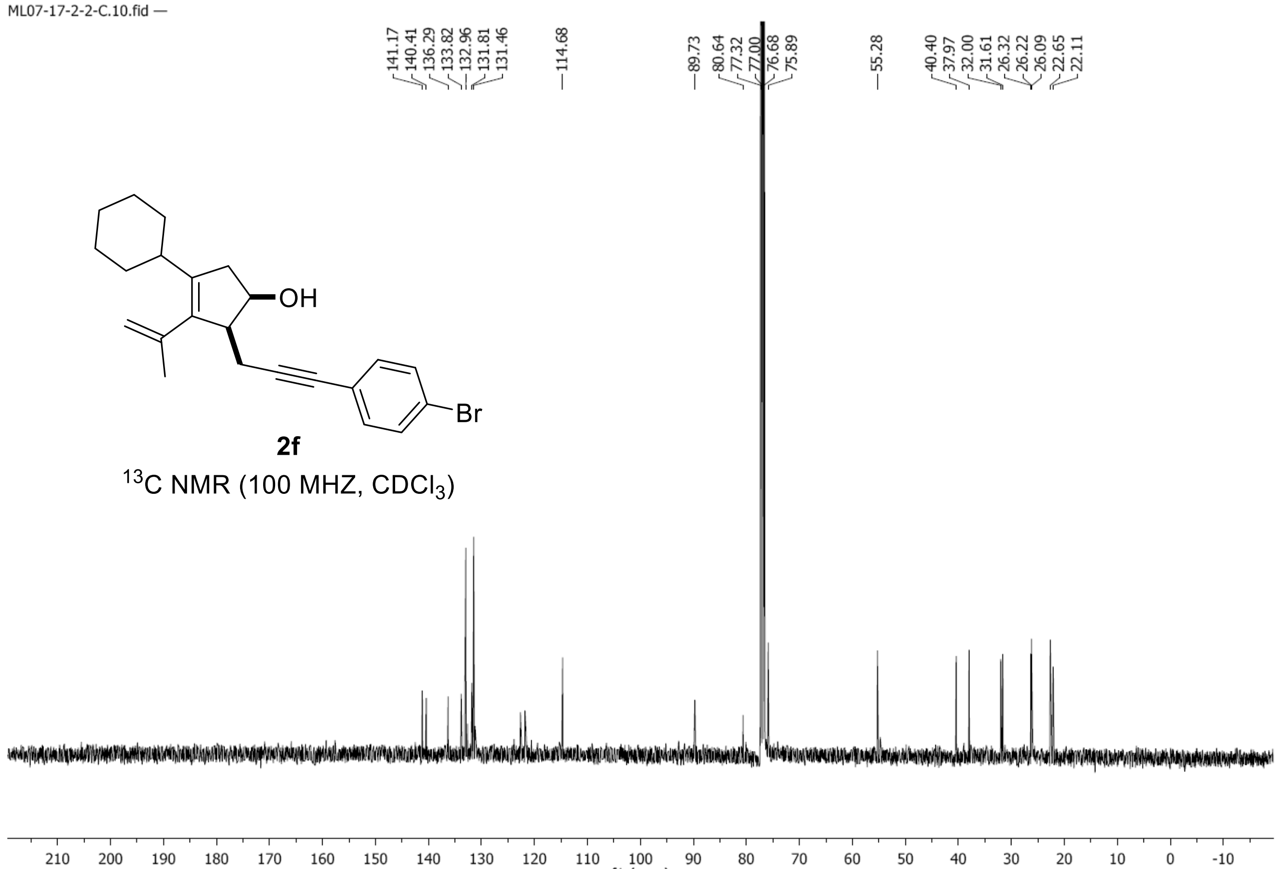
ML07-17-2-2-C.10.fid —
141.17
140.41
136.29
133.82
132.96
131.81
131.46
114.68
89.73
80.64
77.32
77.00
76.68
75.89
55.28
40.40
37.97
32.00
31.61
26.32
26.22
26.09
22.65
22.11
OH
Br
2f
13C NMR (100 MHZ, CDCl3)
f1 (ppm)

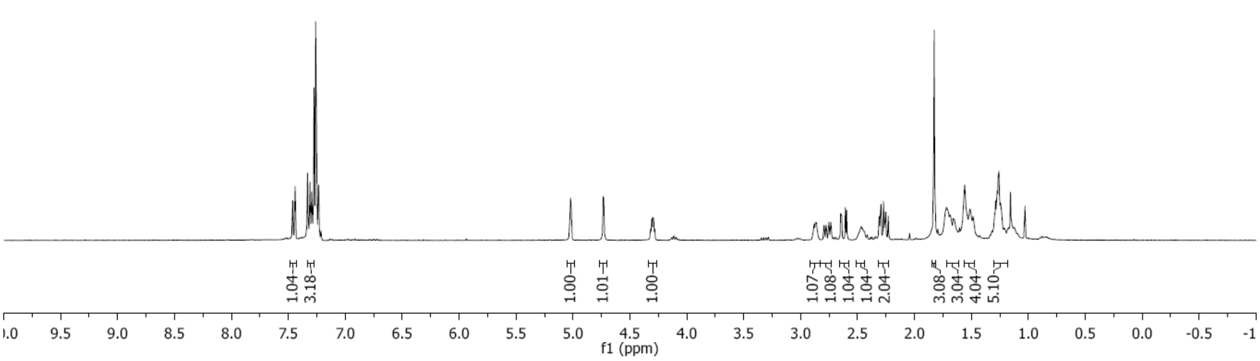

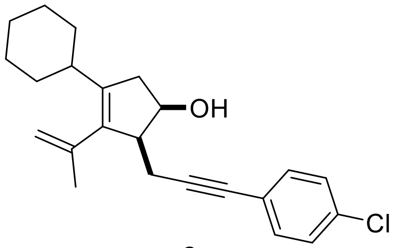

2g

^{1}H NMR (400 MHZ, $CDCl_3$)

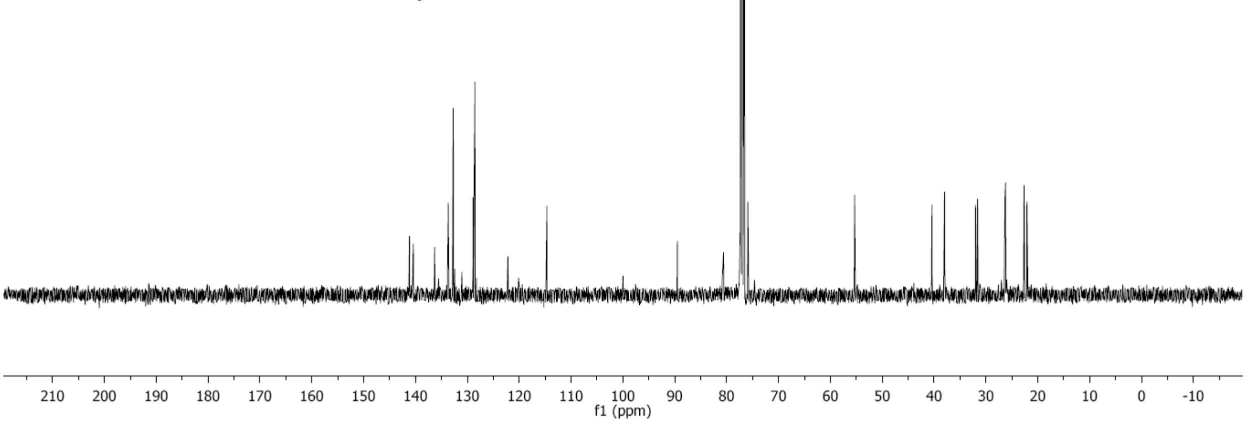

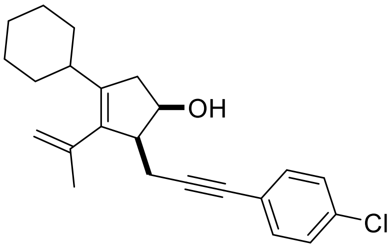

2g

^{13}C NMR (100 MHZ, $CDCl_3$)

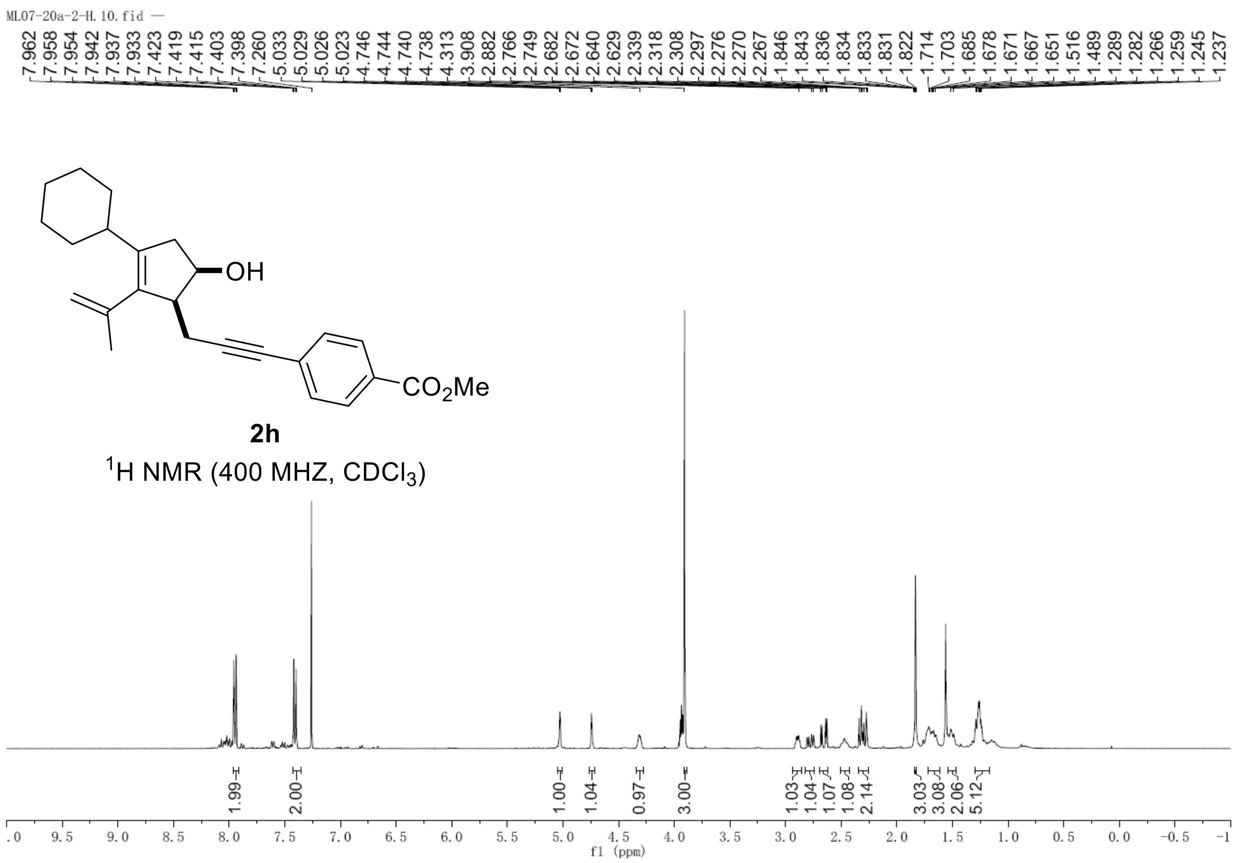
ML07-20a-2-H.10.fid
7.962
7.958
7.954
7.942
7.937
7.933
7.423
7.419
7.415
7.403
7.398
7.260
5.033
5.029
5.026
5.023
4.746
4.744
4.740
4.738
4.313
3.908
2.882
2.766
2.749
2.682
2.672
2.640
2.629
2.339
2.318
2.308
2.297
2.276
2.270
2.267
1.846
1.843
1.836
1.834
1.833
1.831
1.822
1.714
1.703
1.685
1.678
1.671
1.667
1.651
1.516
1.489
1.289
1.282
1.266
1.259
1.245
1.237
OH
CO2Me
2h
1H NMR (400 MHZ, CDCl3)
1.99
2.00
1.00
1.04
0.97
3.00
1.03
1.04
1.07
1.08
2.14
3.03
3.08
2.06
5.12
f1 (ppm)

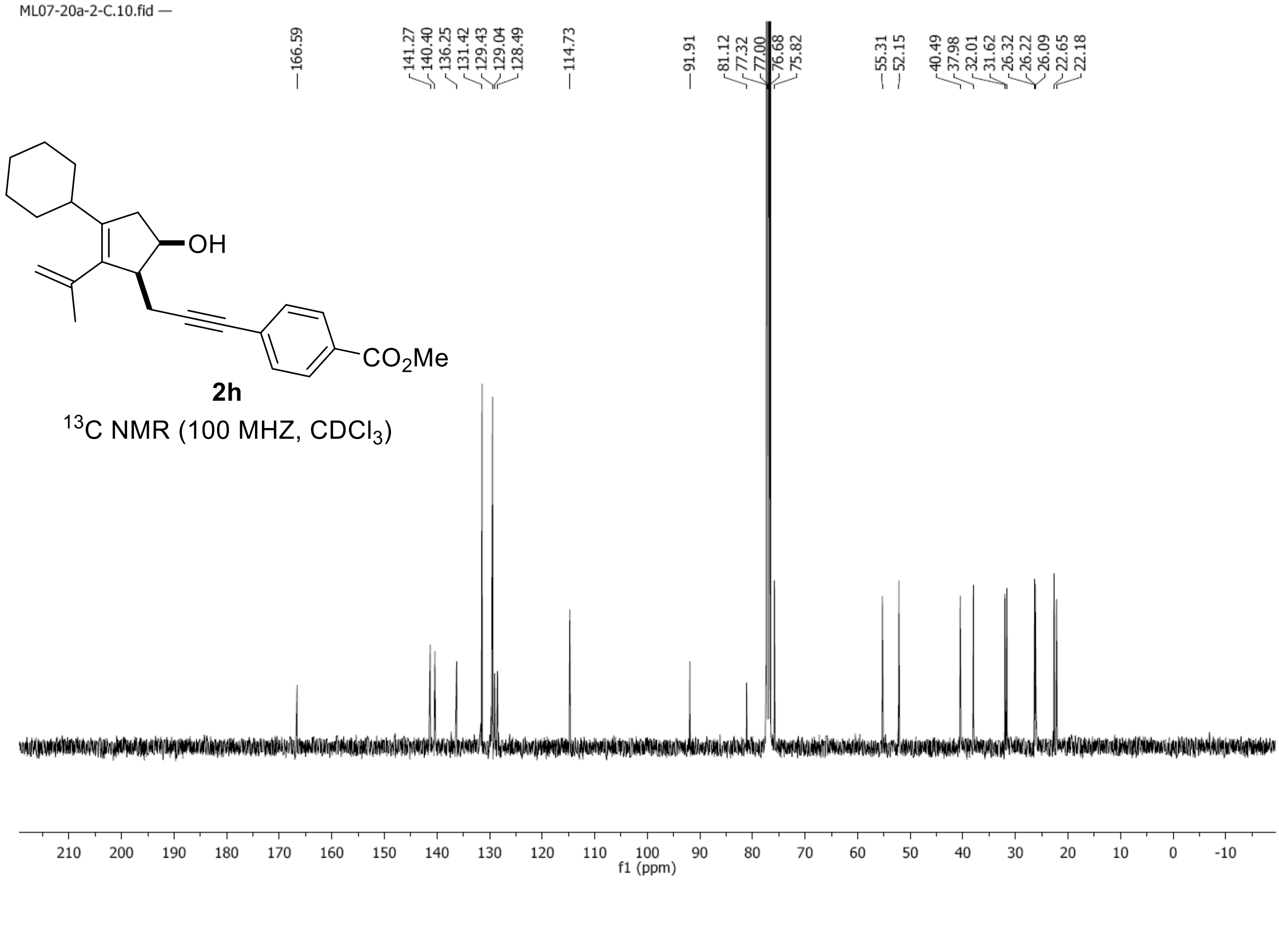
ML07-20a-2-C.10.fid
166.59
141.27
140.40
136.25
131.42
129.43
129.04
128.49
114.73
91.91
81.12
77.32
77.00
76.68
75.82
55.31
52.15
40.49
37.98
32.01
31.62
26.32
26.22
26.09
22.65
22.18
OH
CO2Me
2h
13C NMR (100 MHZ, CDCl3)
f1 (ppm)

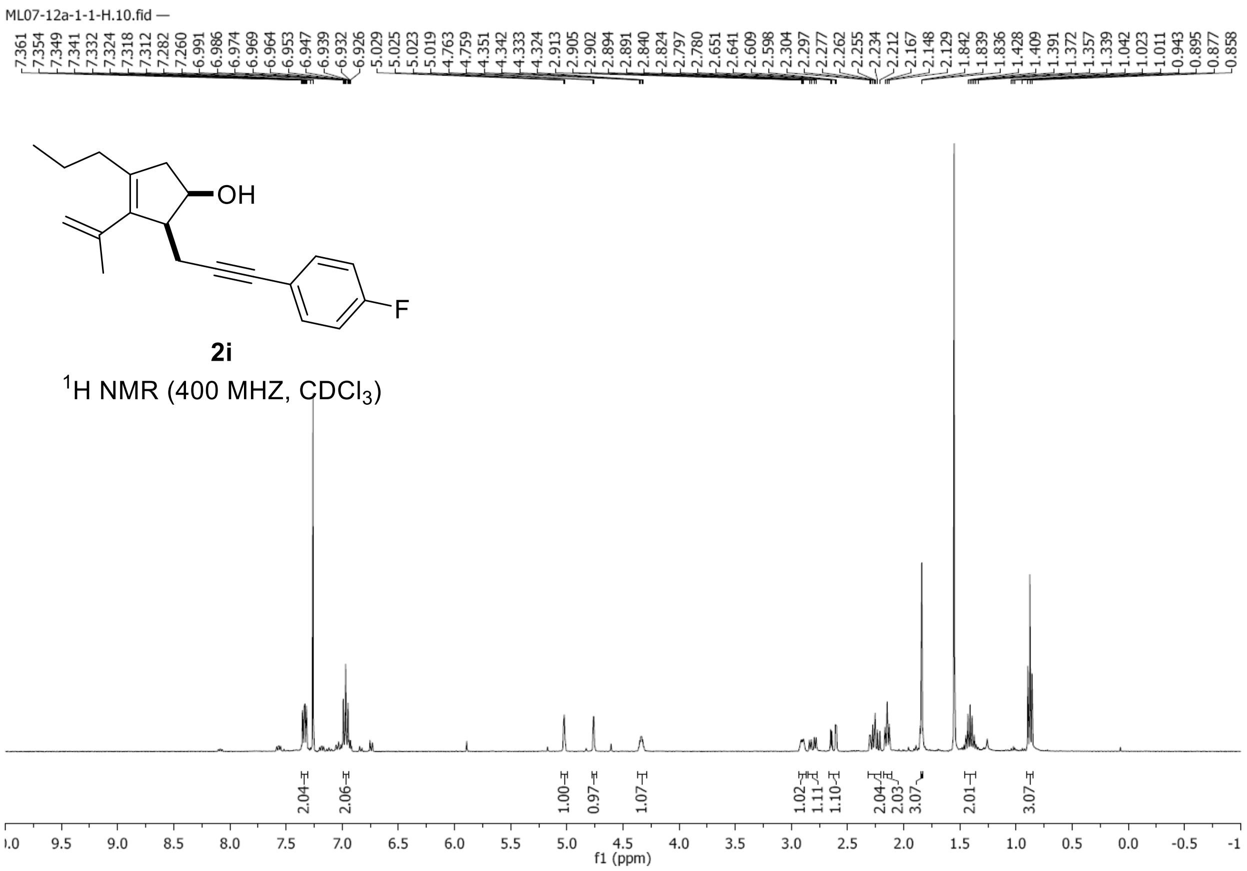
ML07-12a-1-1-H.10.fid —
7.361 7.354 7.349 7.341 7.332 7.324 7.318 7.312 7.282 7.260 6.991 6.986 6.974 6.969 6.964 6.953 6.947 6.939 6.932 6.926 5.029 5.025 5.023 5.019 4.763 4.759 4.351 4.342 4.333 4.324 2.913 2.905 2.902 2.894 2.891 2.840 2.824 2.797 2.780 2.651 2.641 2.609 2.598 2.304 2.297 2.277 2.262 2.255 2.234 2.212 2.167 2.148 2.129 1.842 1.839 1.836 1.428 1.409 1.391 1.372 1.357 1.339 1.042 1.023 1.011 0.943 0.895 0.877 0.858
OH
F
2i
^{1}H NMR (400 MHZ, CDCl$_3$)
2.04 2.06 1.00 0.97 1.07 1.02 1.11 1.10 2.04 2.03 3.07 2.01 3.07
f1 (ppm)

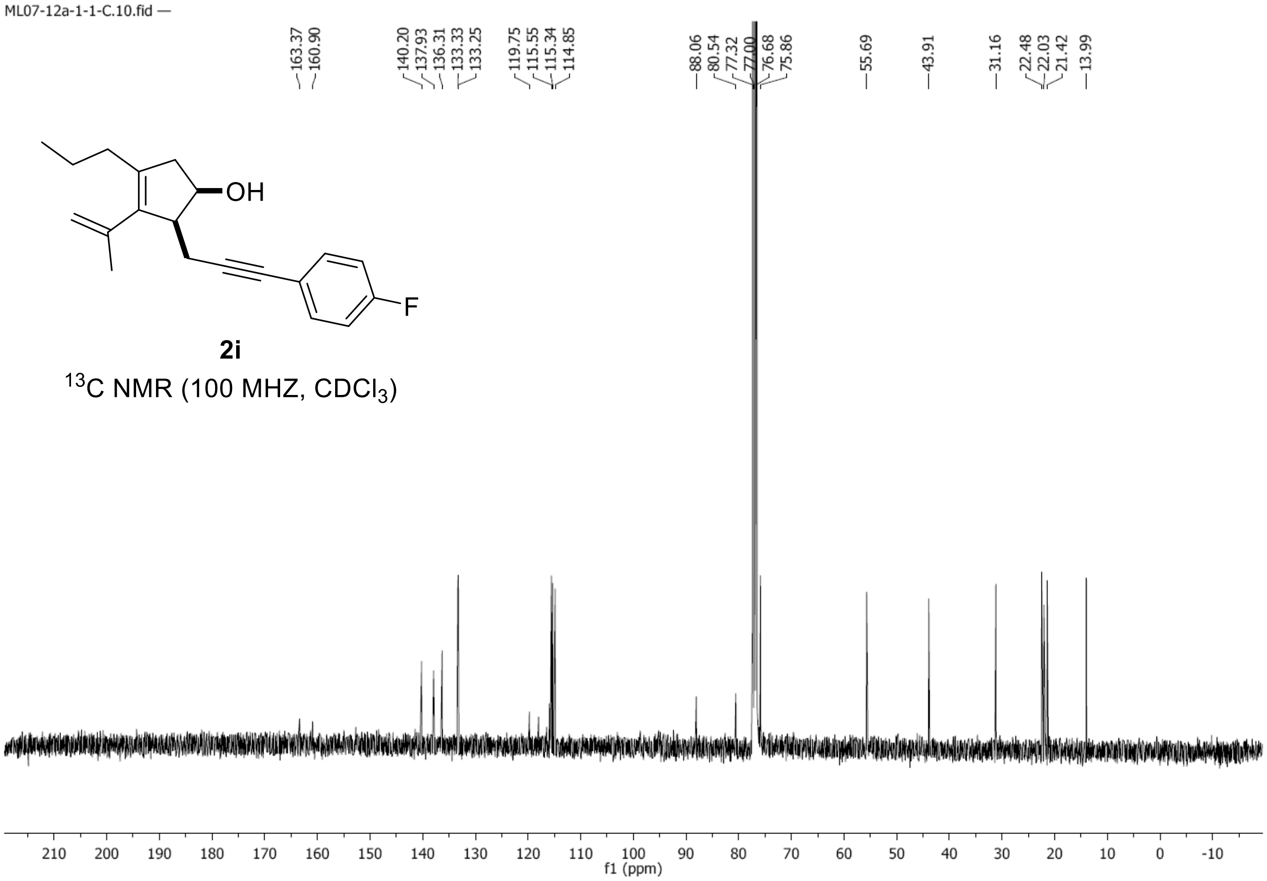
ML07-12a-1-1-C.10.fid —
163.37 160.90 140.20 137.93 136.31 133.33 133.25 119.75 115.55 115.34 114.85 88.06 80.54 77.32 77.00 76.68 75.86 55.69 43.91 31.16 22.48 22.03 21.42 13.99
OH
F
2i
^{13}C NMR (100 MHZ, CDCl$_3$)
f1 (ppm)

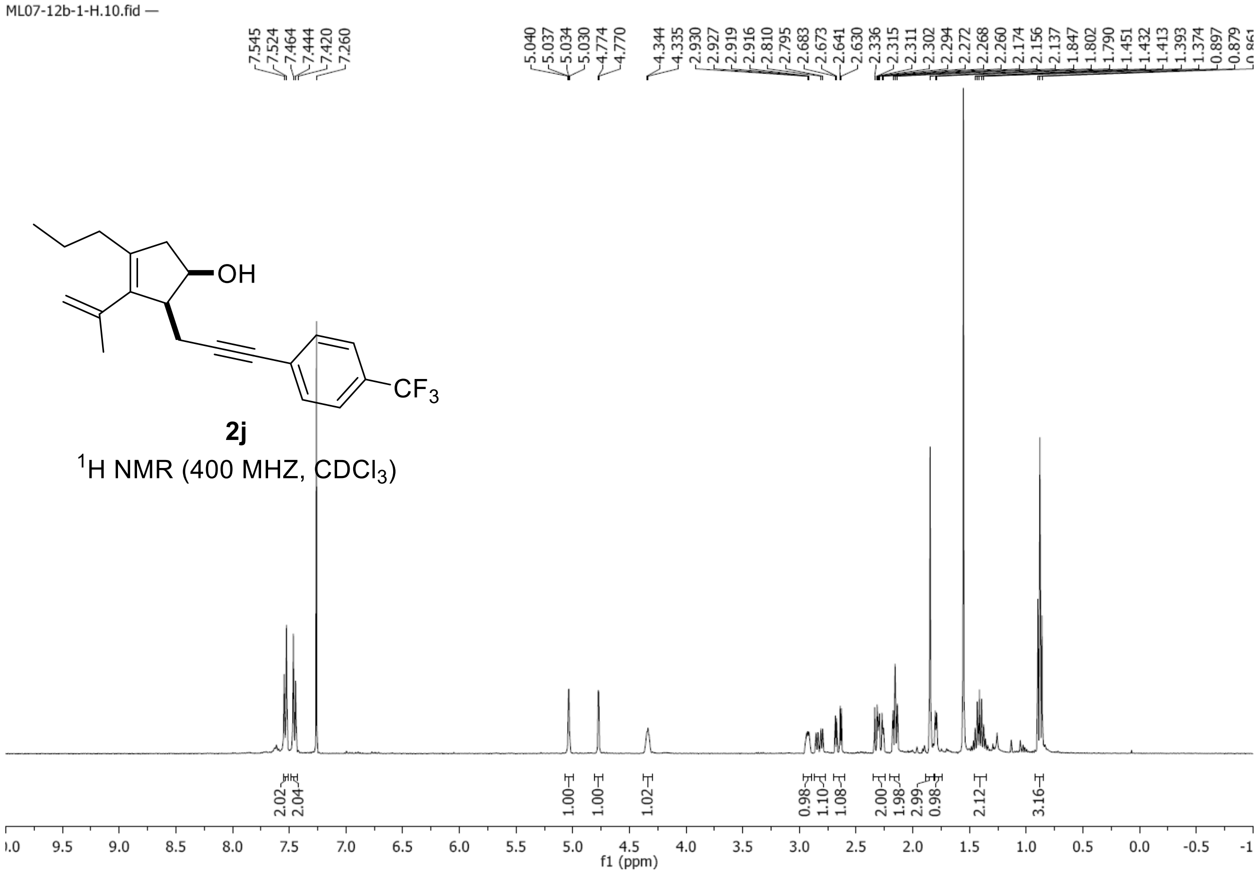
ML07-12b-1-H.10.fid —
7.545
7.524
7.464
7.444
7.420
7.260
5.040
5.037
5.034
5.030
4.774
4.770
4.344
4.335
2.930
2.927
2.919
2.916
2.810
2.795
2.683
2.673
2.641
2.630
2.336
2.315
2.311
2.302
2.294
2.272
2.268
2.260
2.174
2.156
2.137
1.847
1.802
1.790
1.451
1.432
1.413
1.393
1.374
0.897
0.879
OH
CF3
2j
1H NMR (400 MHZ, CDCl3)
2.02
2.04
1.00
1.00
1.02
0.98
1.10
1.08
2.00
1.98
2.99
0.98
2.12
3.16
f1 (ppm)

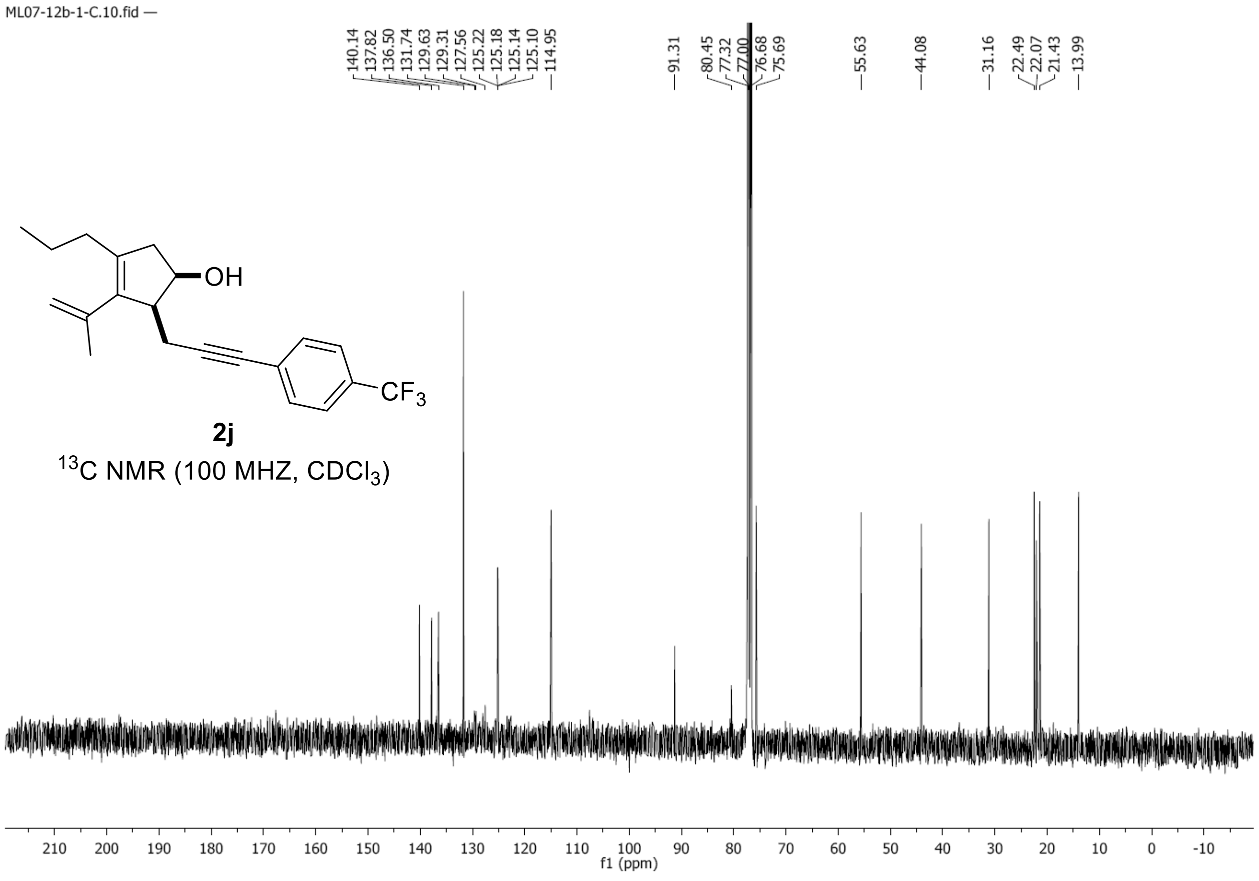
ML07-12b-1-C.10.fid —
140.14
137.82
136.50
131.74
129.63
129.31
127.56
125.22
125.18
125.14
125.10
114.95
91.31
80.45
77.32
77.00
76.68
75.69
55.63
44.08
31.16
22.49
22.07
21.43
13.99
OH
CF3
2j
13C NMR (100 MHZ, CDCl3)
f1 (ppm)

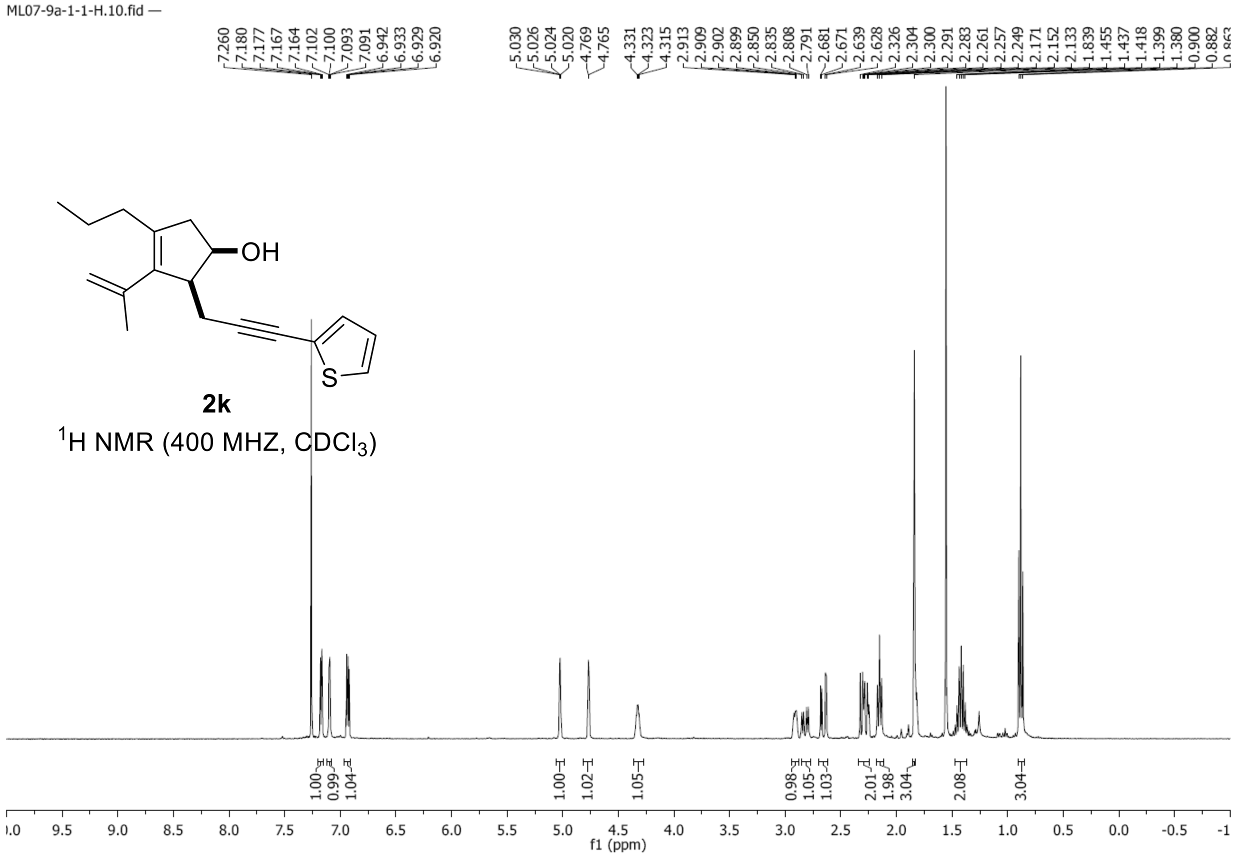
ML07-9a-1-1-H.10.fid
7.260 7.180 7.177 7.167 7.164 7.102 7.100 7.093 7.091 6.942 6.933 6.929 6.920
5.030 5.026 5.024 5.020 4.769 4.765 4.331 4.323 4.315 2.913 2.909 2.902 2.899 2.850 2.835 2.808 2.791 2.681 2.671 2.639 2.628 2.326 2.304 2.300 2.291 2.283 2.261 2.257 2.249 2.171 2.152 2.133 1.839 1.455 1.437 1.418 1.399 1.380 0.900 0.882
OH
S
2k
^{1}H NMR (400 MHZ, CDCl$_3$)
1.00 0.99 1.04 1.00 1.02 1.05 0.98 1.05 1.03 2.01 1.98 3.04 2.08 3.04
f1 (ppm)

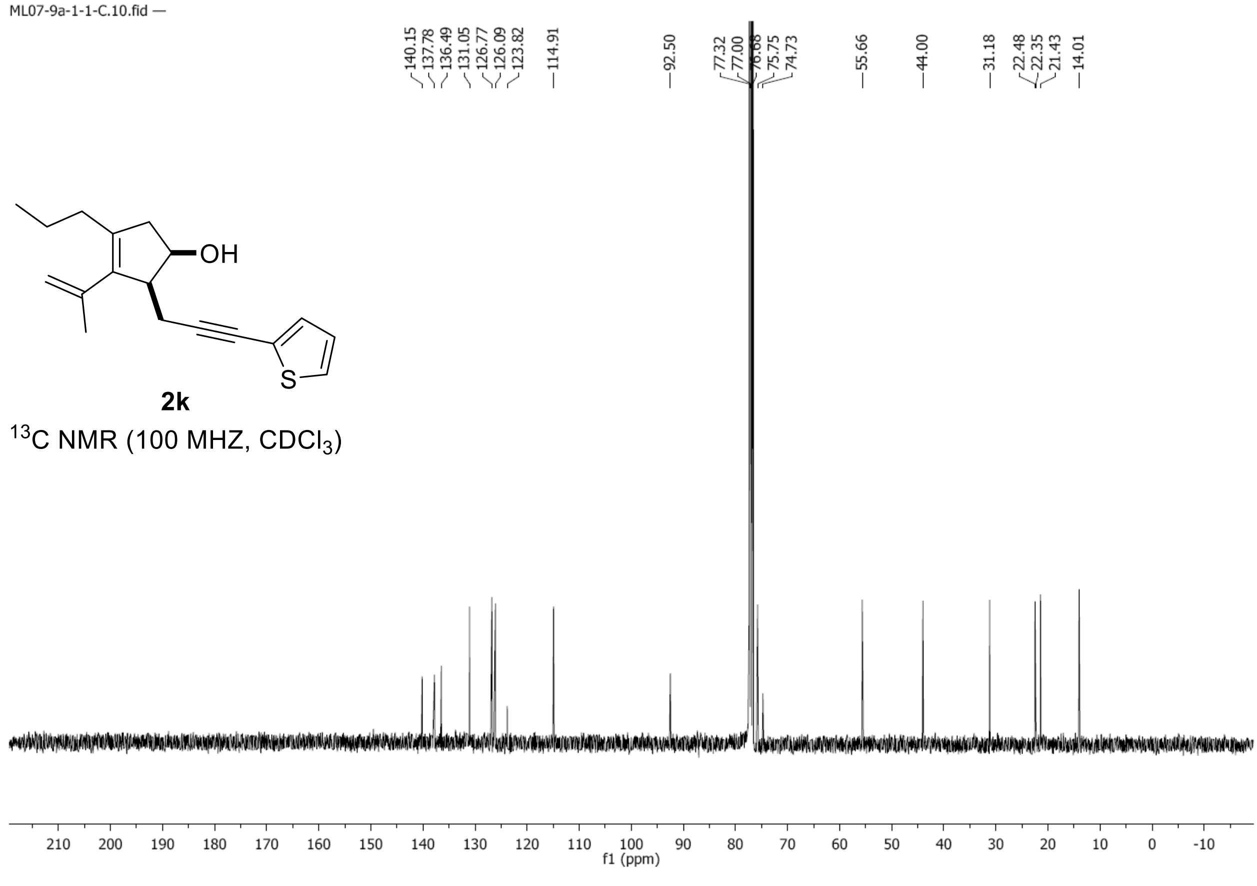
ML07-9a-1-1-C.10.fid
140.15 137.78 136.49 131.05 126.77 126.09 123.82 114.91 92.50 77.32 77.00 76.68 75.75 74.73 55.66 44.00 31.18 22.48 22.35 21.43 14.01
OH
S
2k
^{13}C NMR (100 MHZ, CDCl$_3$)
f1 (ppm)

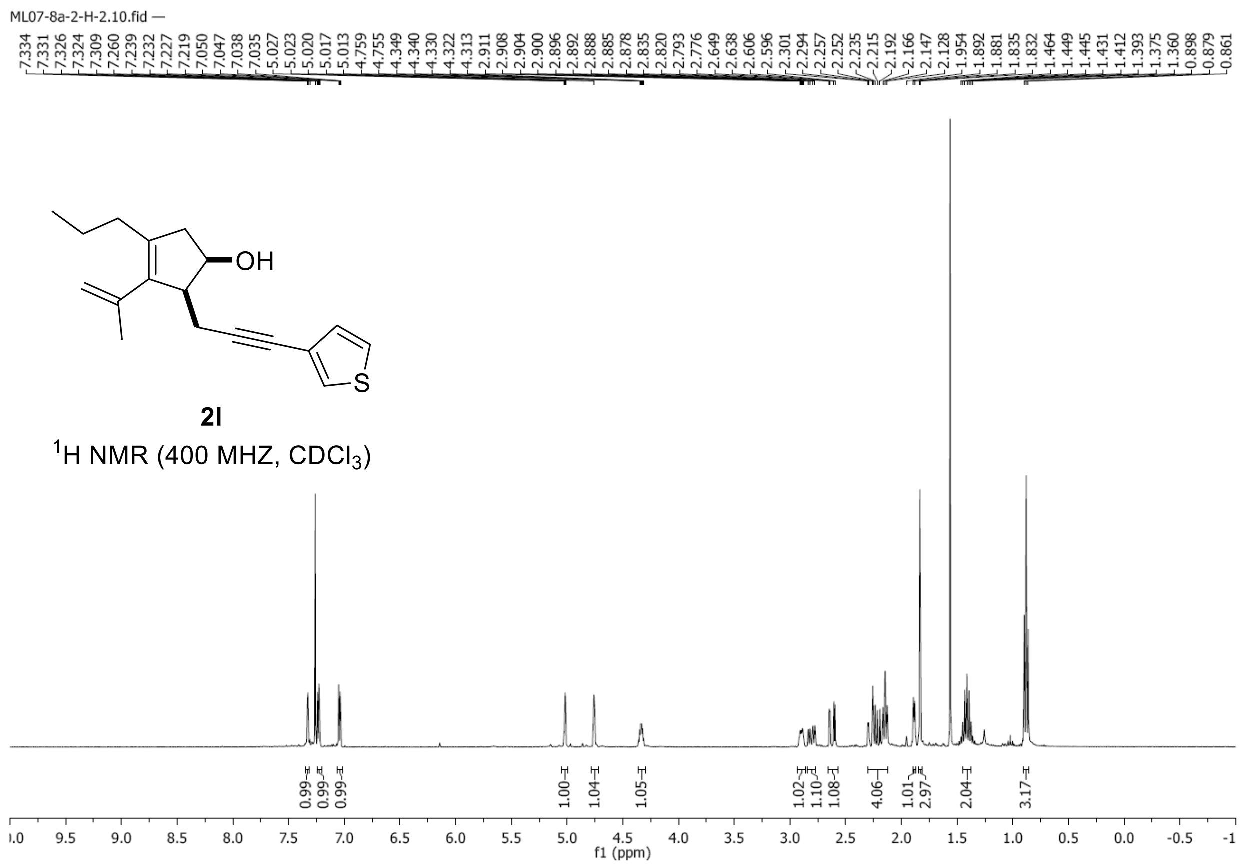

2l

^{1}H NMR (400 MHZ, $CDCl_3$)

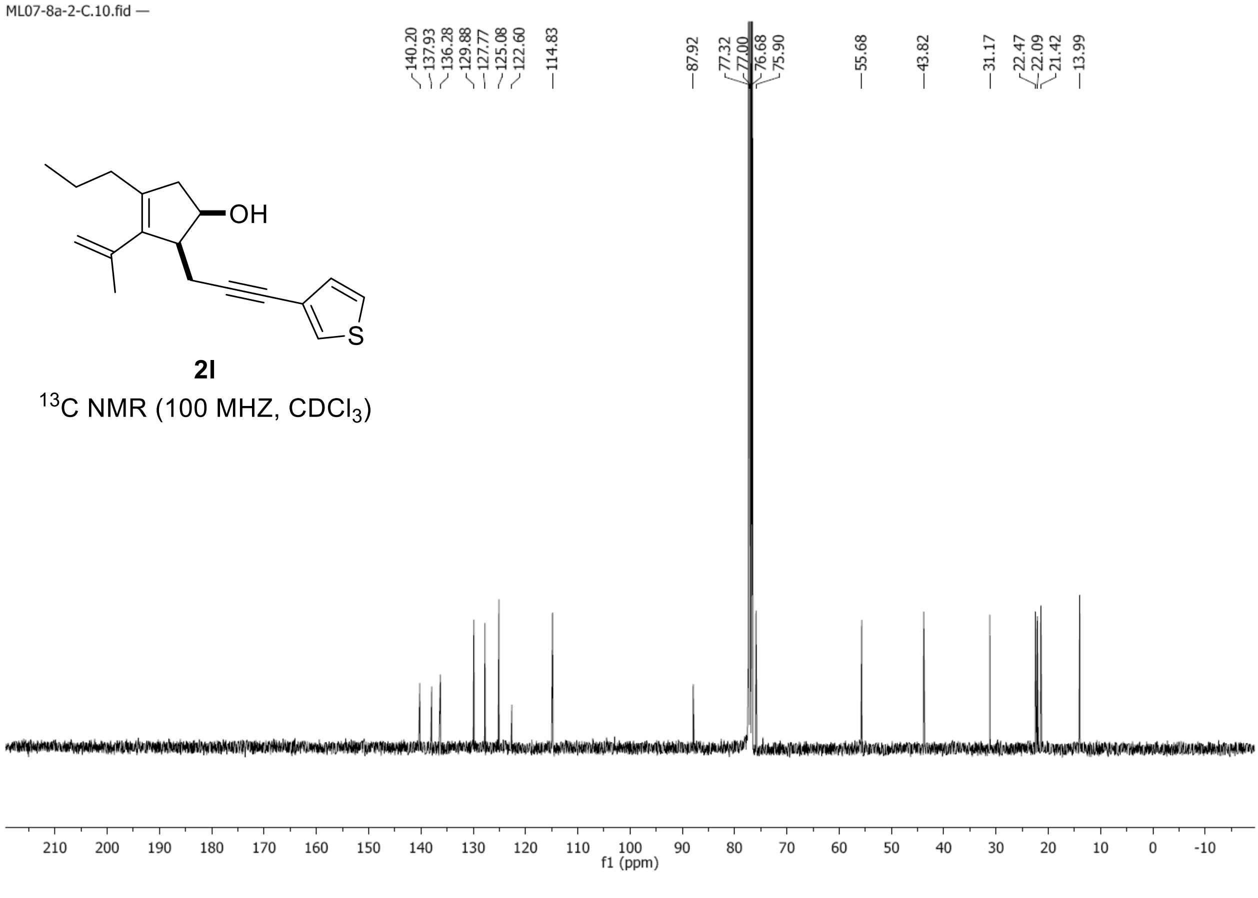

2l

^{13}C NMR (100 MHZ, $CDCl_3$)

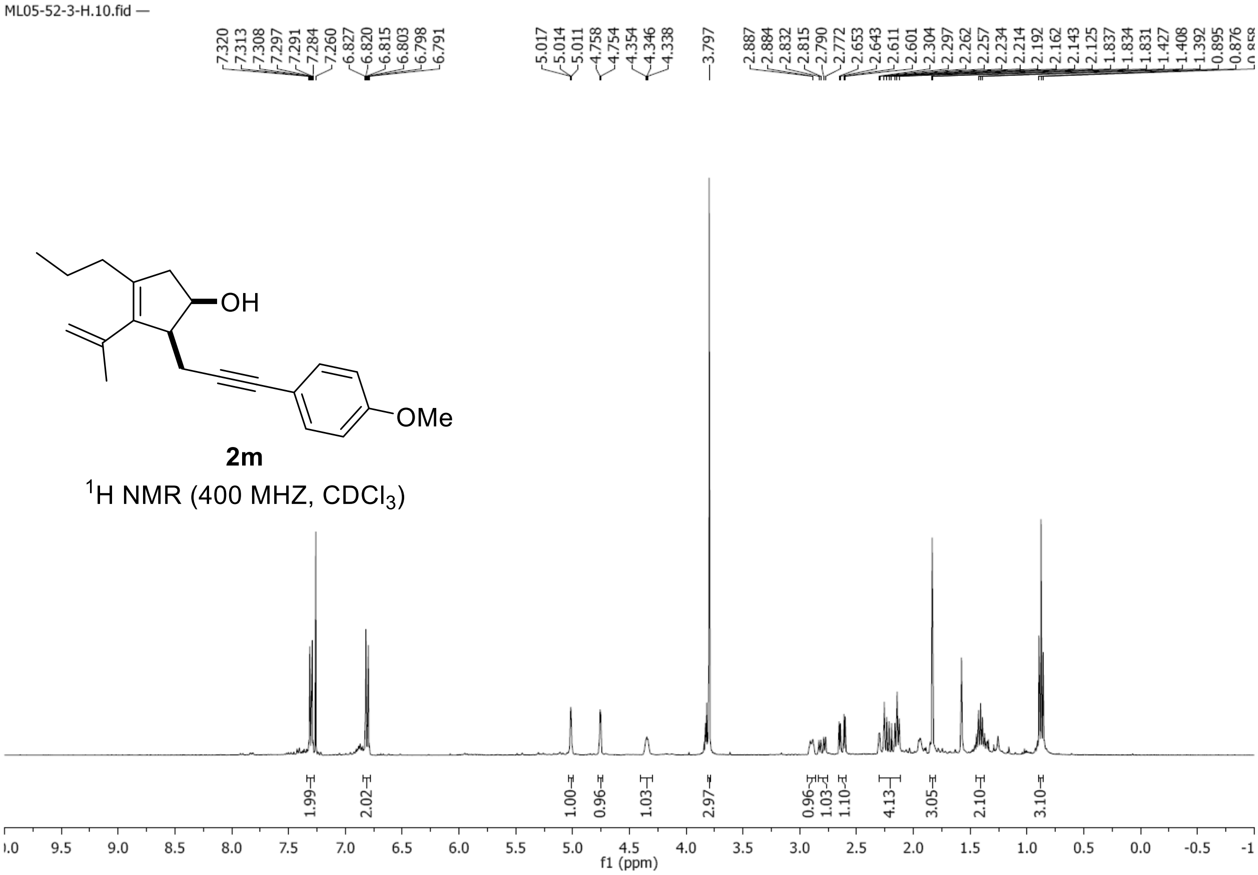
ML05-52-3-H.10.fid
7.320 7.313 7.308 7.297 7.291 7.284 7.260 6.827 6.820 6.815 6.803 6.798 6.791
5.017 5.014 5.011 4.758 4.754 4.354 4.346 4.338
3.797
2.887 2.884 2.832 2.815 2.790 2.772 2.653 2.643 2.611 2.601 2.304 2.297 2.262 2.257 2.234 2.214 2.192 2.162 2.143 2.125 1.837 1.834 1.831 1.427 1.408 1.392 0.895 0.876
OH
OMe
2m
^{1}H NMR (400 MHZ, CDCl$_3$)
1.99 2.02 1.00 0.96 1.03 2.97 0.96 1.03 1.10 4.13 3.05 2.10 3.10
f1 (ppm)

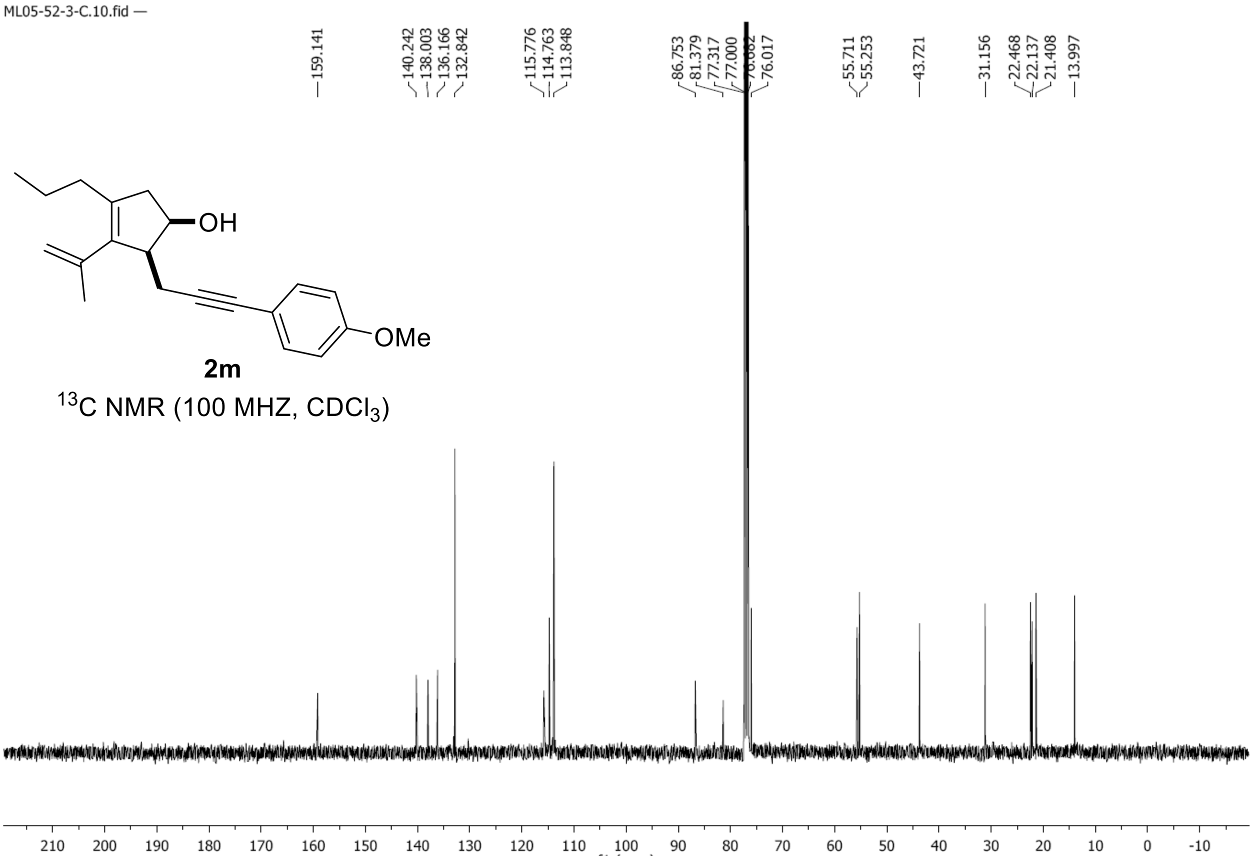
ML05-52-3-C.10.fid
159.141
140.242 138.003 136.166 132.842
115.776 114.763 113.848
86.753 81.379 77.317 77.000 76.682 76.017
55.711 55.253
43.721
31.156 22.468 22.137 21.408 13.997
OH
OMe
2m
^{13}C NMR (100 MHZ, CDCl$_3$)
f1 (ppm)

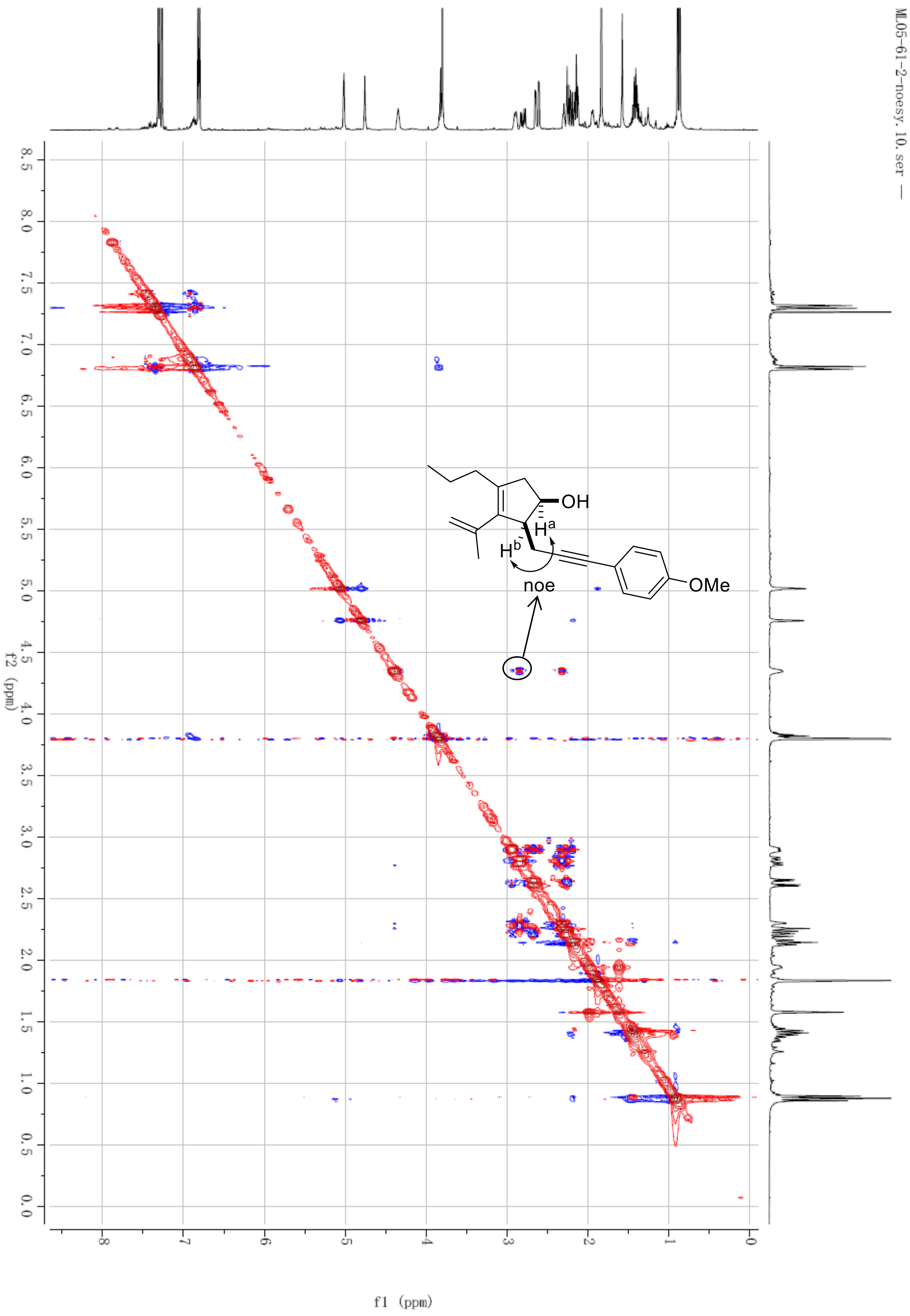
ML05-61-2-noesy.10.ser
OH
H^a
H^b
noe
OMe
f2 (ppm)
f1 (ppm)

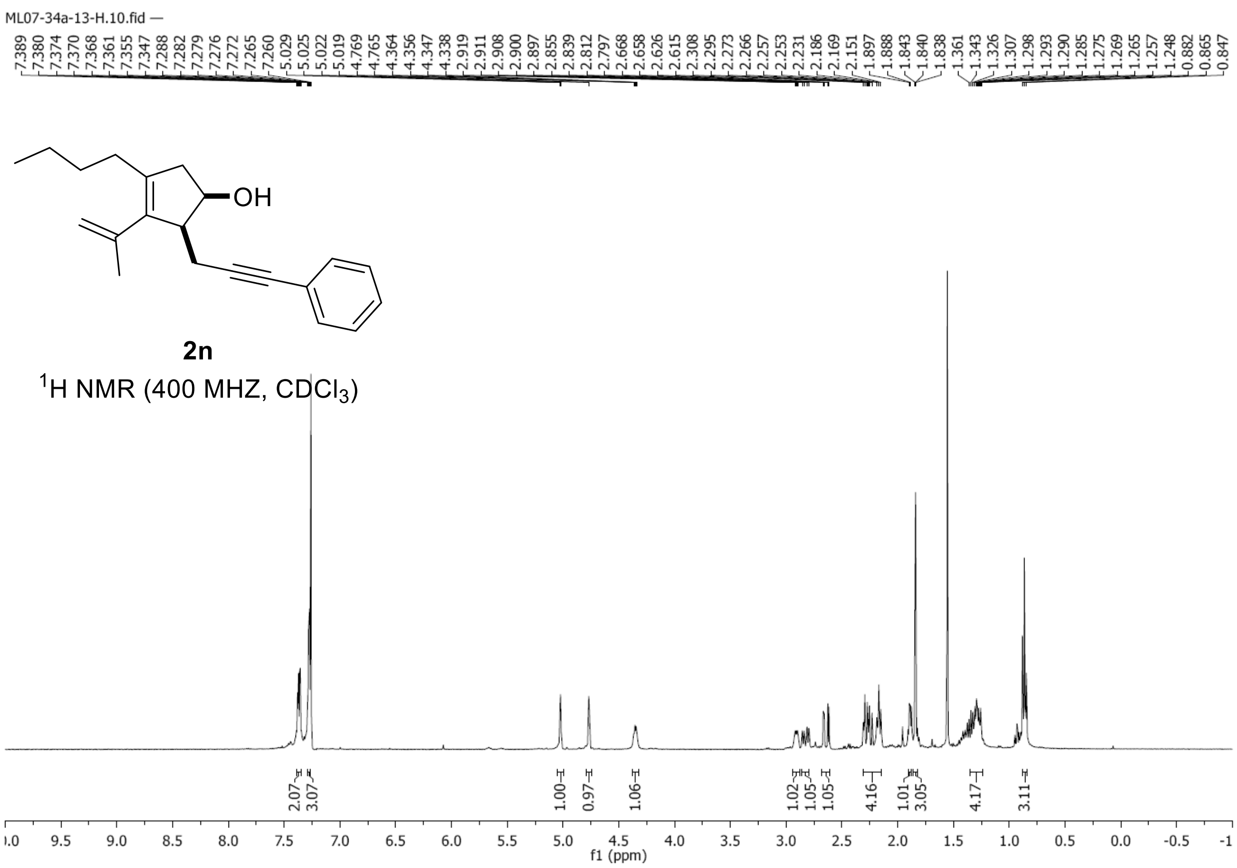

2n

^{1}H NMR (400 MHZ, $CDCl_3$)

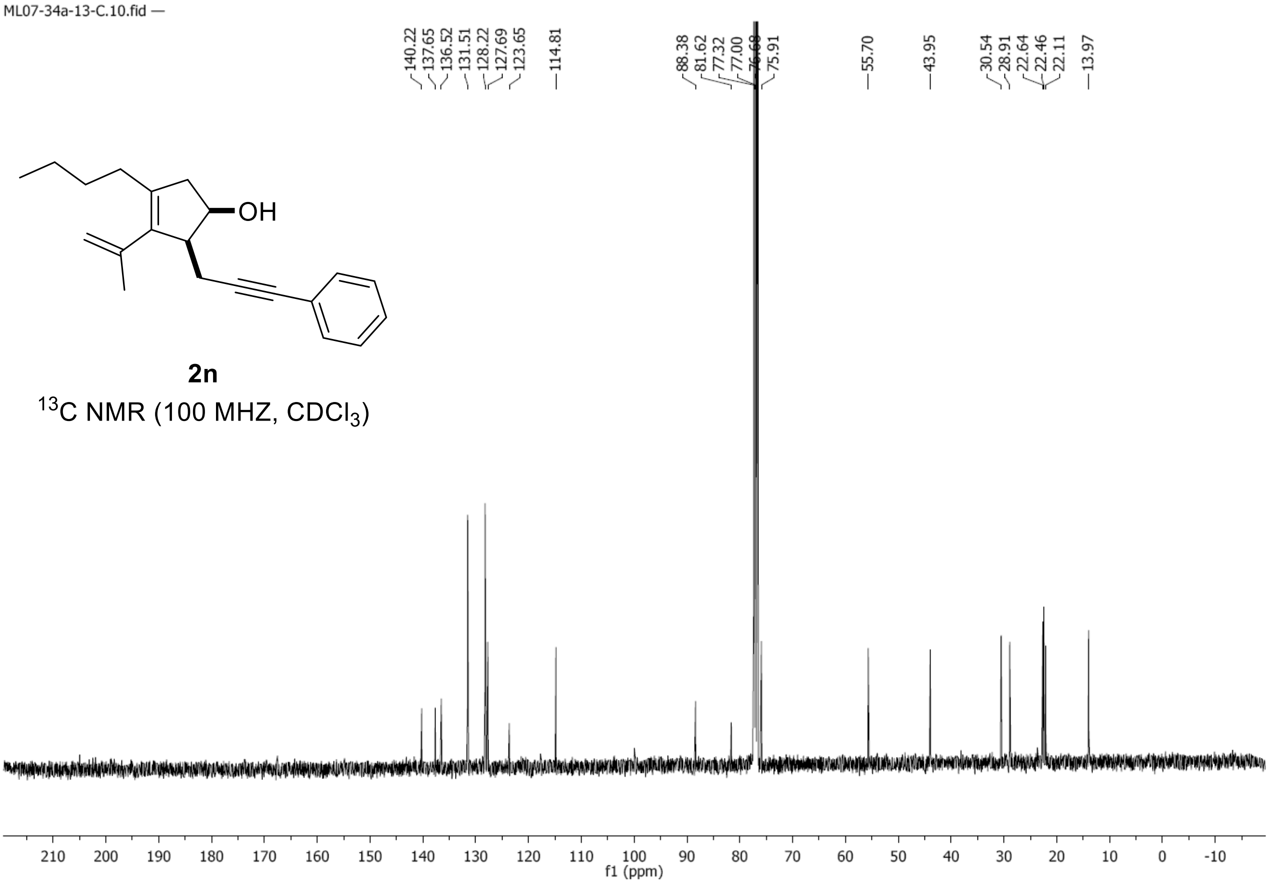

2n

^{13}C NMR (100 MHZ, $CDCl_3$)

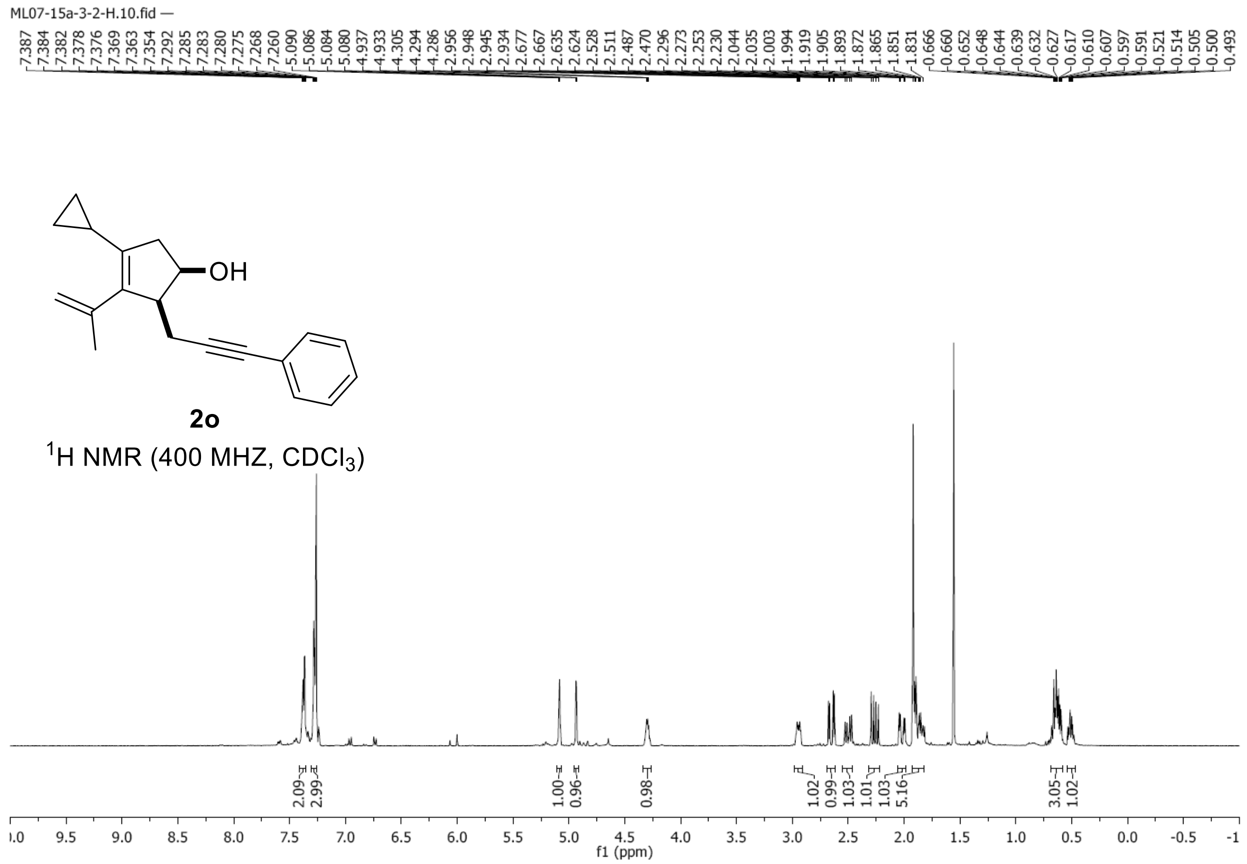
ML07-15a-3-2-H.10.fid
7.387 7.384 7.382 7.378 7.376 7.369 7.363 7.354 7.292 7.285 7.283 7.280 7.275 7.268 7.260 5.090 5.086 5.084 5.080 4.937 4.933 4.305 4.294 4.286 2.956 2.948 2.945 2.934 2.677 2.667 2.635 2.624 2.528 2.511 2.487 2.470 2.296 2.273 2.253 2.230 2.044 2.035 2.003 1.994 1.919 1.905 1.893 1.872 1.865 1.851 1.831 0.666 0.660 0.652 0.648 0.644 0.639 0.632 0.627 0.617 0.610 0.607 0.597 0.591 0.521 0.514 0.505 0.500 0.493
OH
2o
^{1}H NMR (400 MHZ, $CDCl_3$)
2.09 2.99 1.00 0.96 0.98 1.02 0.99 1.03 1.01 1.03 5.16 3.05 1.02
f1 (ppm)

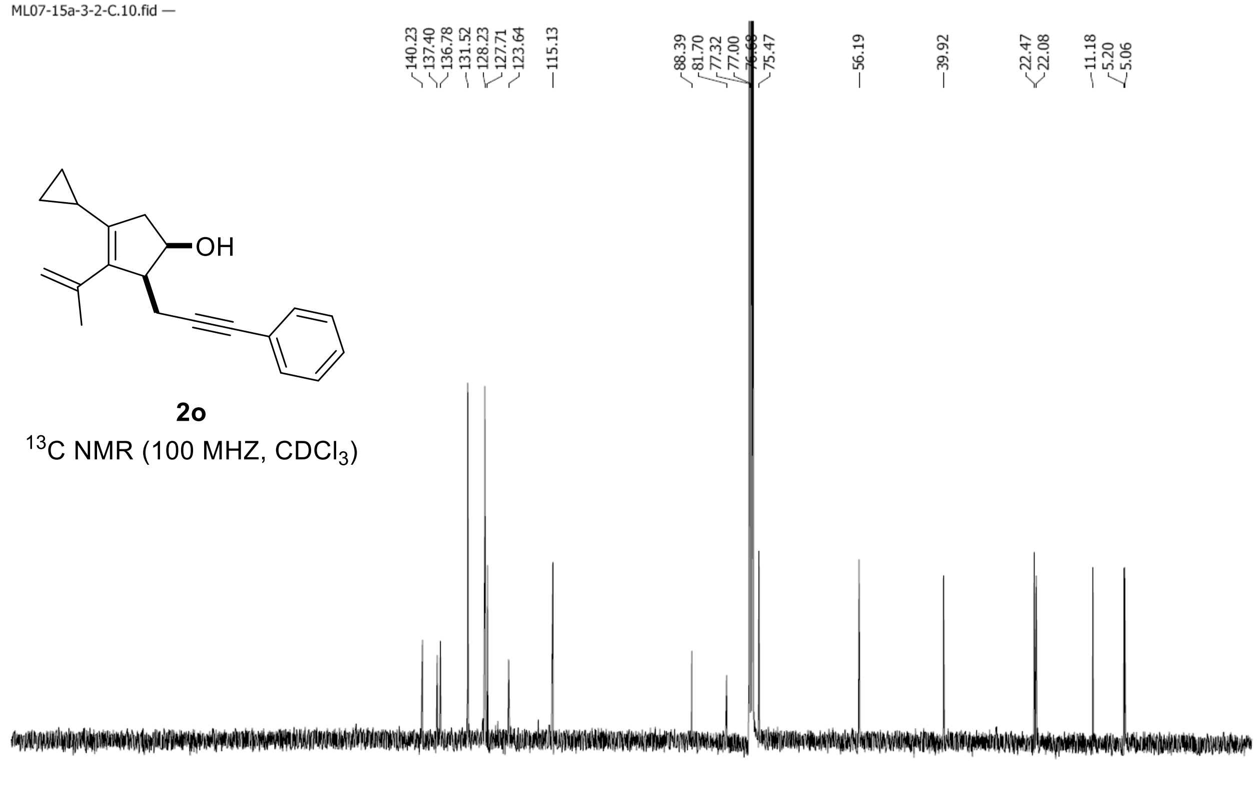
ML07-15a-3-2-C.10.fid
140.23 137.40 136.78 131.52 128.23 127.71 123.64 115.13 88.39 81.70 77.32 77.00 76.68 75.47 56.19 39.92 22.47 22.08 11.18 5.20 5.06
OH
2o
^{13}C NMR (100 MHZ, $CDCl_3$)
f1 (ppm)

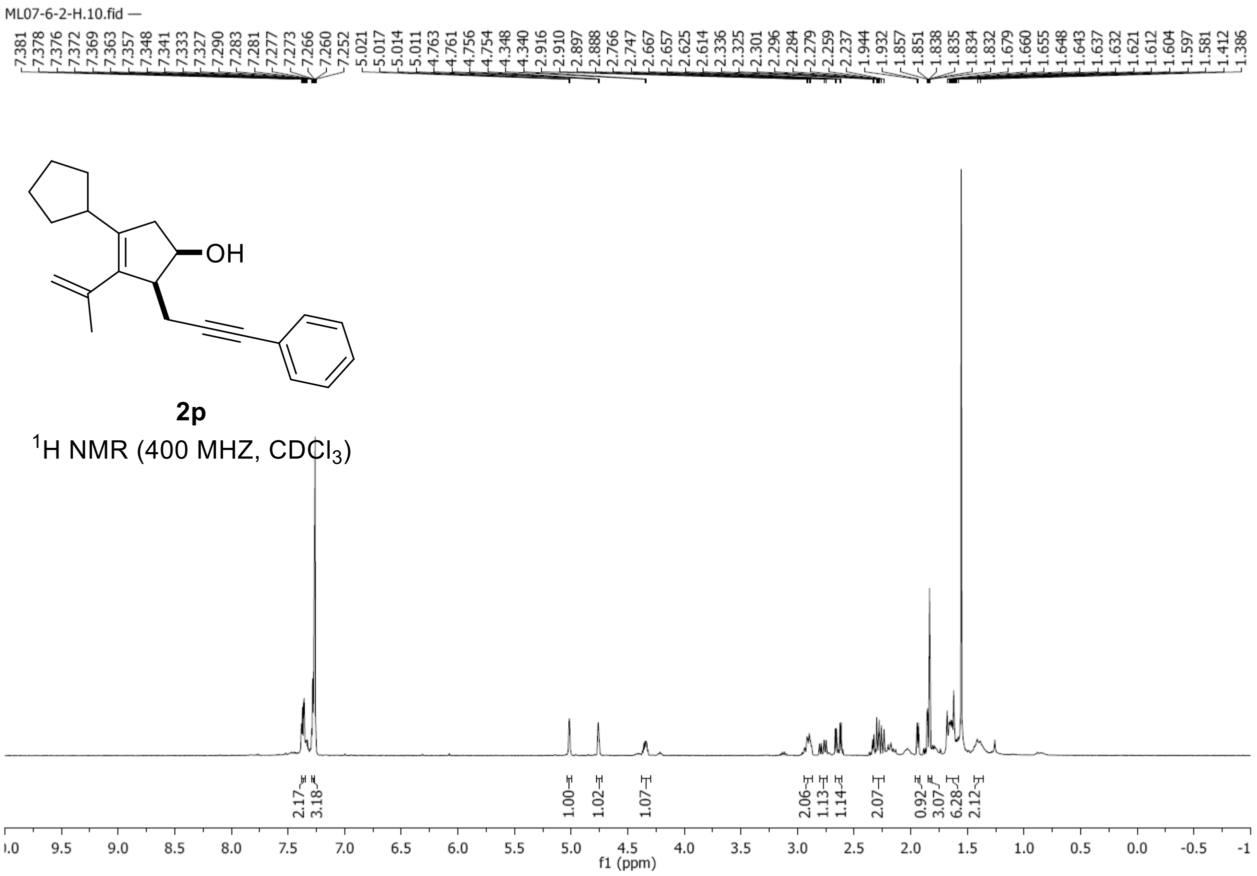

2p

^{1}H NMR (400 MHZ, $CDCl_3$)

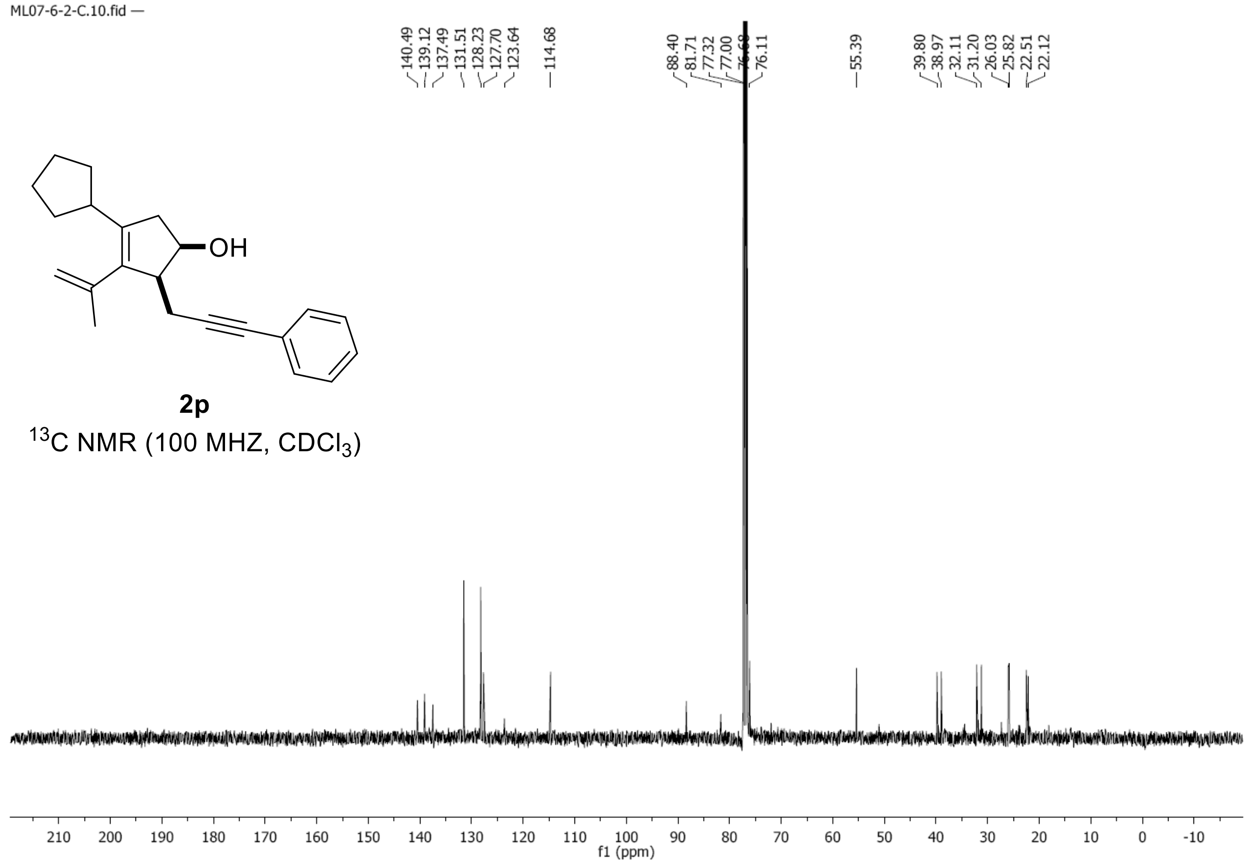

2p

^{13}C NMR (100 MHZ, $CDCl_3$)

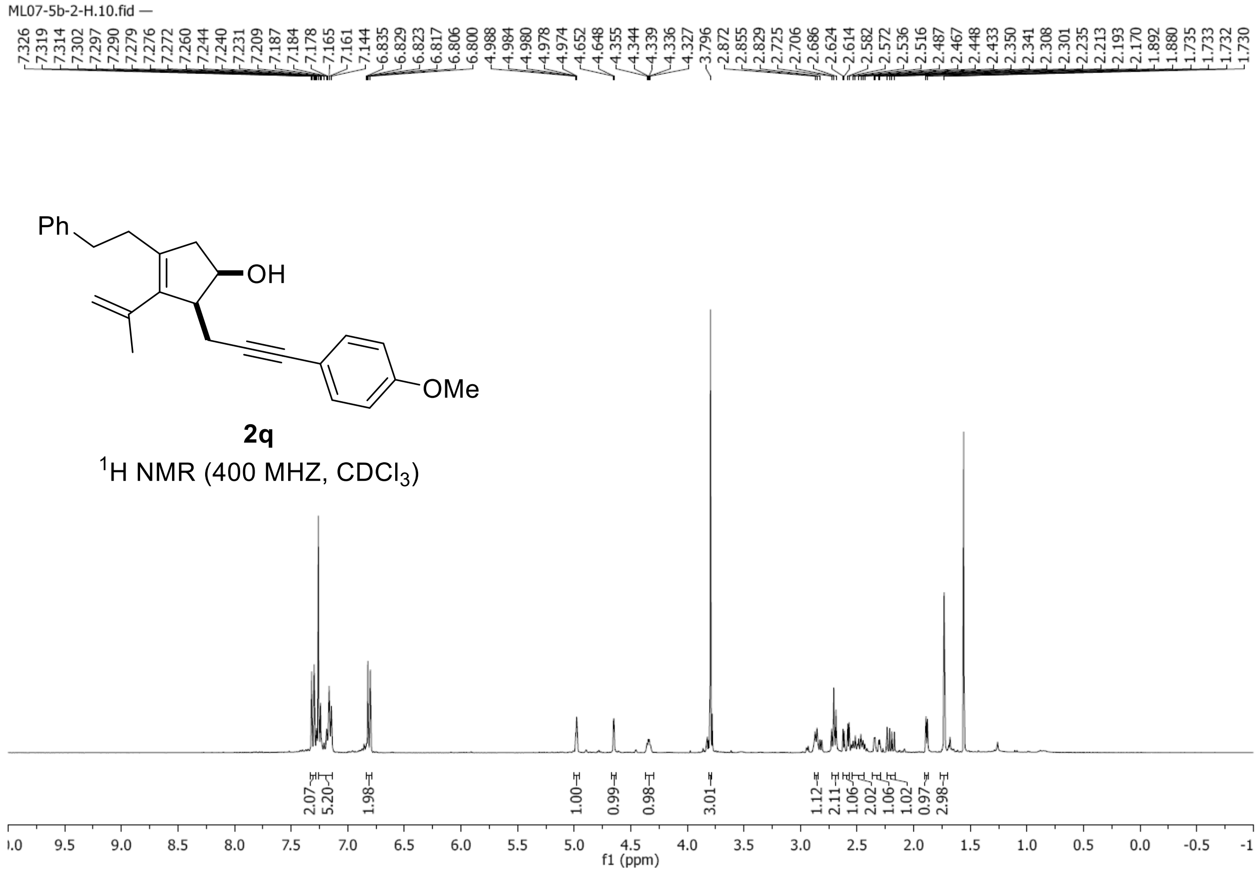
ML07-5b-2-H.10.fid —
7.326 7.319 7.314 7.302 7.297 7.290 7.279 7.276 7.272 7.260 7.244 7.240 7.231 7.209 7.187 7.184 7.178 7.165 7.161 7.144 6.835 6.829 6.823 6.817 6.806 6.800 4.988 4.984 4.980 4.978 4.974 4.652 4.648 4.355 4.344 4.339 4.336 4.327 3.796 2.872 2.855 2.829 2.725 2.706 2.686 2.624 2.614 2.582 2.572 2.536 2.516 2.487 2.467 2.448 2.433 2.350 2.341 2.308 2.301 2.235 2.213 2.193 2.170 1.892 1.880 1.735 1.733 1.732 1.730
Ph
OH
OMe
2q
1H NMR (400 MHZ, CDCl3)
2.07 5.20 1.98 1.00 0.99 0.98 3.01 1.12 2.11 1.06 2.02 1.06 1.02 0.97 2.98
f1 (ppm)

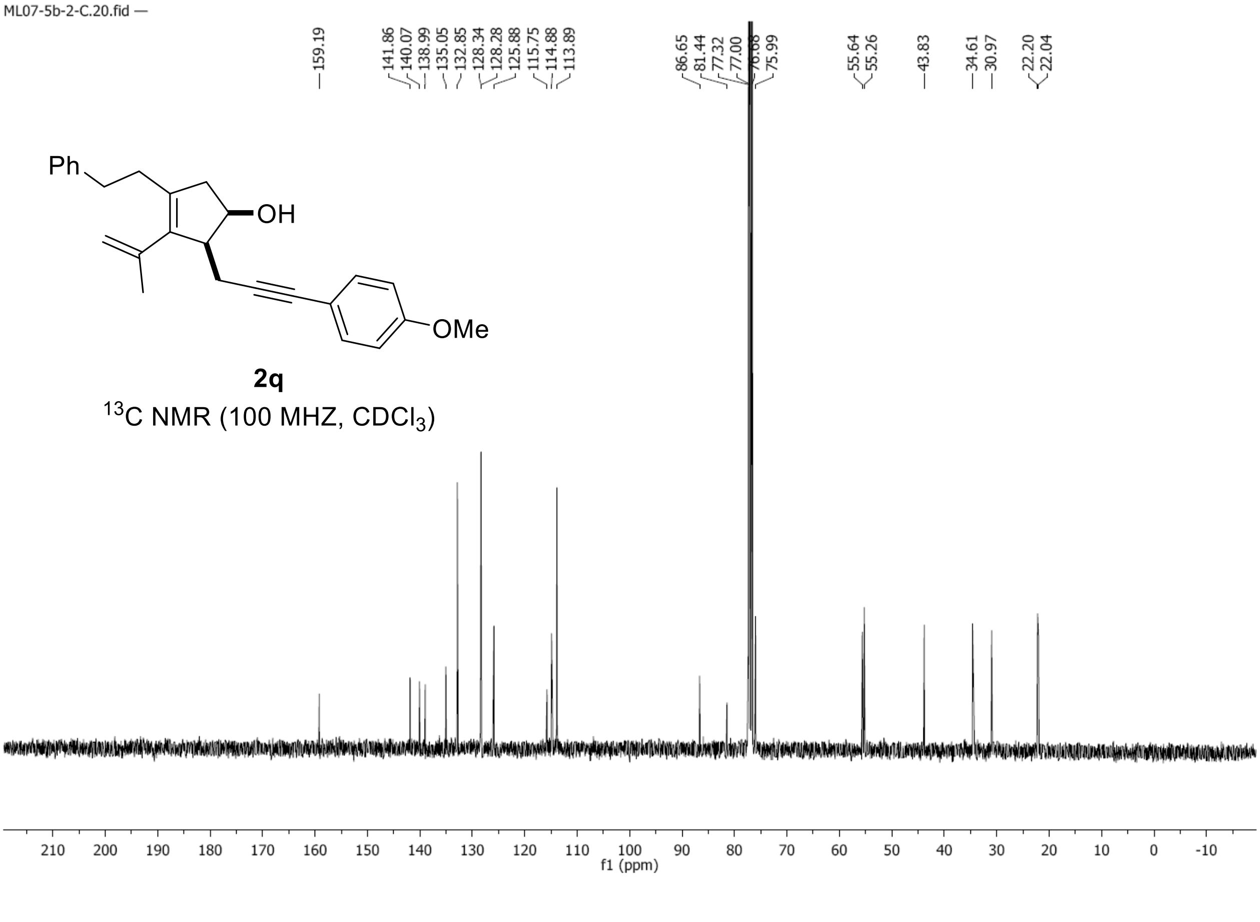
ML07-5b-2-C.20.fid —
159.19 141.86 140.07 138.99 135.05 132.85 128.34 128.28 125.88 115.75 114.88 113.89 86.65 81.44 77.32 77.00 76.68 75.99 55.64 55.26 43.83 34.61 30.97 22.20 22.04
Ph
OH
OMe
2q
13C NMR (100 MHZ, CDCl3)
f1 (ppm)

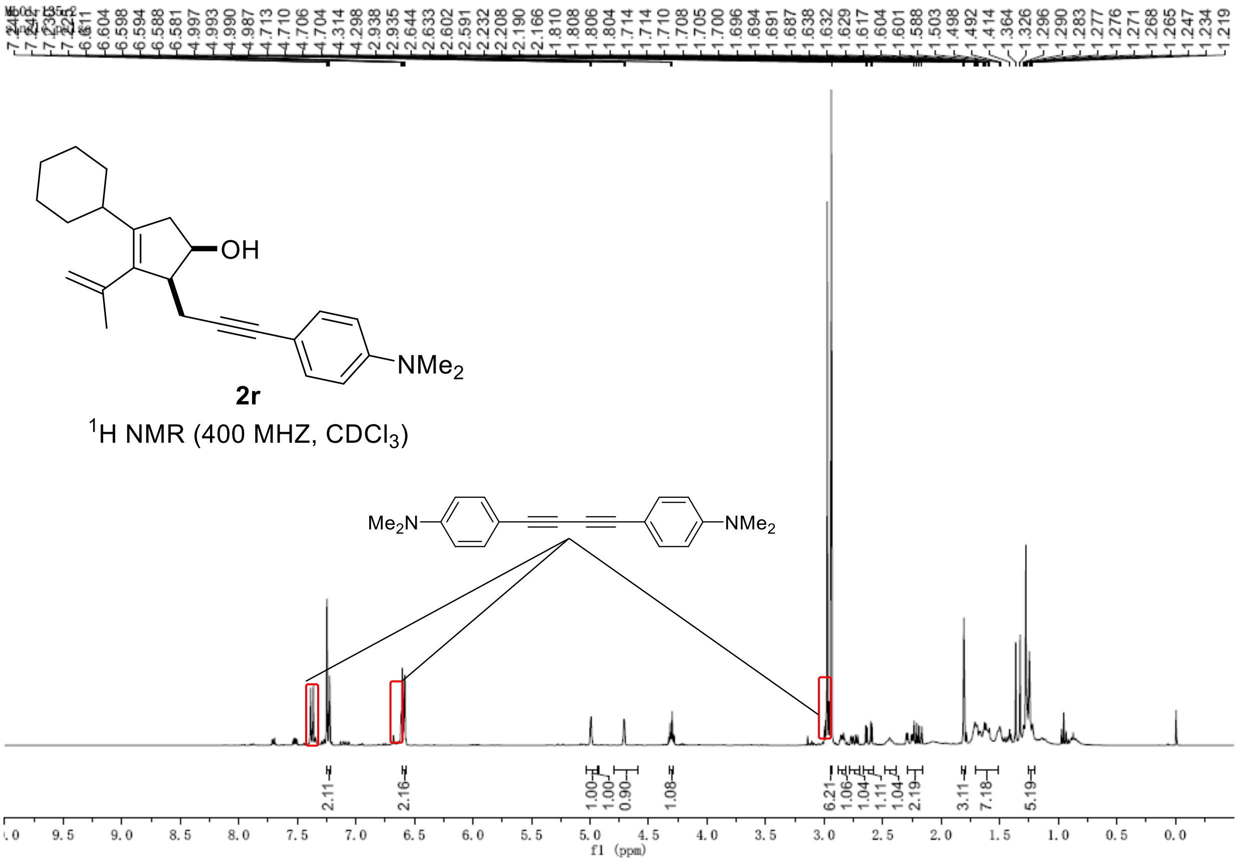
6.611 6.604 6.598 6.594 6.588 6.581 4.997 4.993 4.990 4.987 4.713 4.710 4.706 4.704 4.314 4.298 2.938 2.935 2.644 2.633 2.602 2.591 2.232 2.208 2.190 2.166 1.810 1.808 1.806 1.804 1.714 1.714 1.710 1.708 1.705 1.700 1.696 1.694 1.691 1.687 1.638 1.632 1.629 1.617 1.604 1.601 1.588 1.503 1.498 1.492 1.414 1.364 1.326 1.296 1.290 1.283 1.277 1.271 1.268 1.265 1.247 1.234 1.219
OH
NMe2
2r
1H NMR (400 MHZ, CDCl3)
Me2N
NMe2
2.11
2.16
1.00
1.00
0.90
1.08
6.21
1.06
1.04
1.11
1.04
2.19
3.11
7.18
5.19
f1 (ppm)

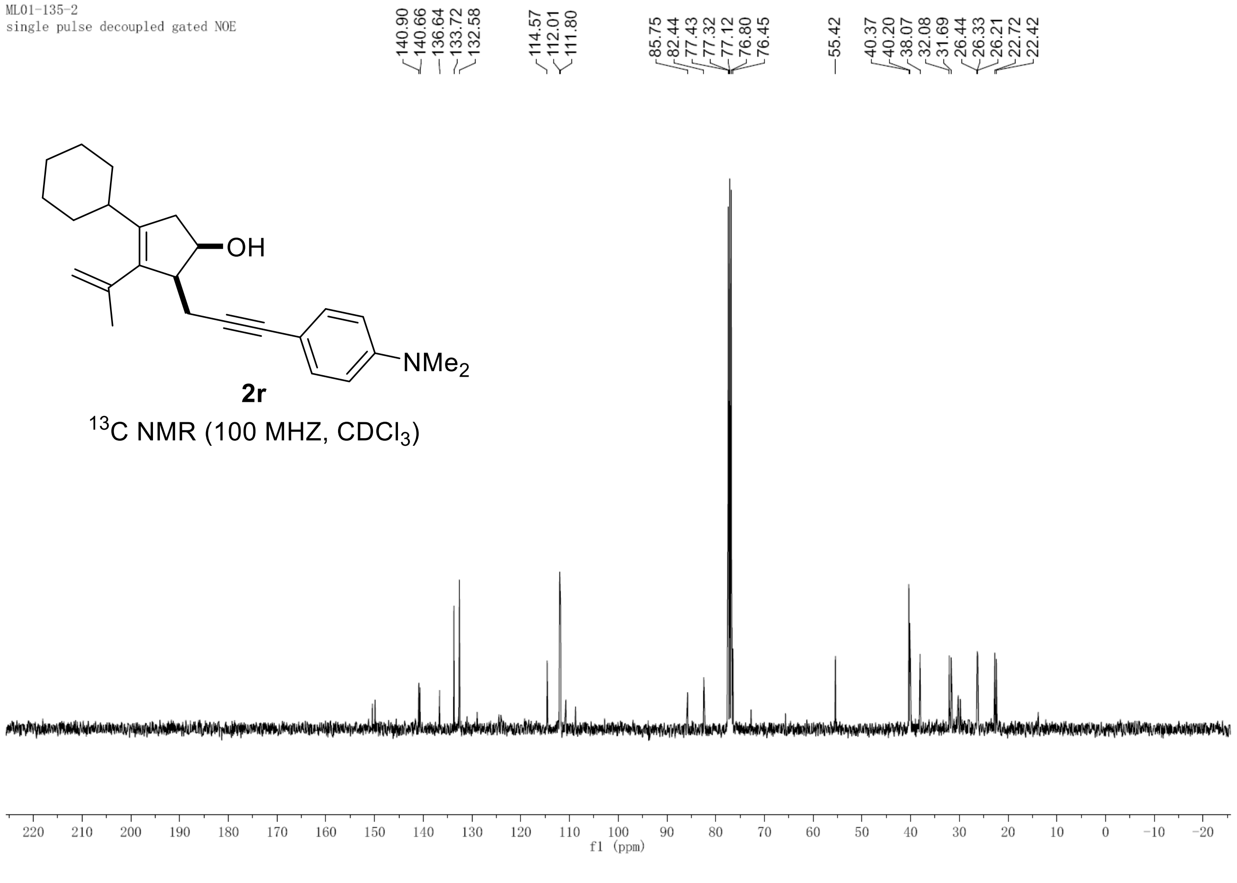
ML01-135-2
single pulse decoupled gated NOE
140.90 140.66 136.64 133.72 132.58
114.57 112.01 111.80
85.75 82.44 77.43 77.32 77.12 76.80 76.45
55.42
40.37 40.20 38.07 32.08 31.69 26.44 26.33 26.21 22.72 22.42
OH
NMe2
2r
13C NMR (100 MHZ, CDCl3)
f1 (ppm)

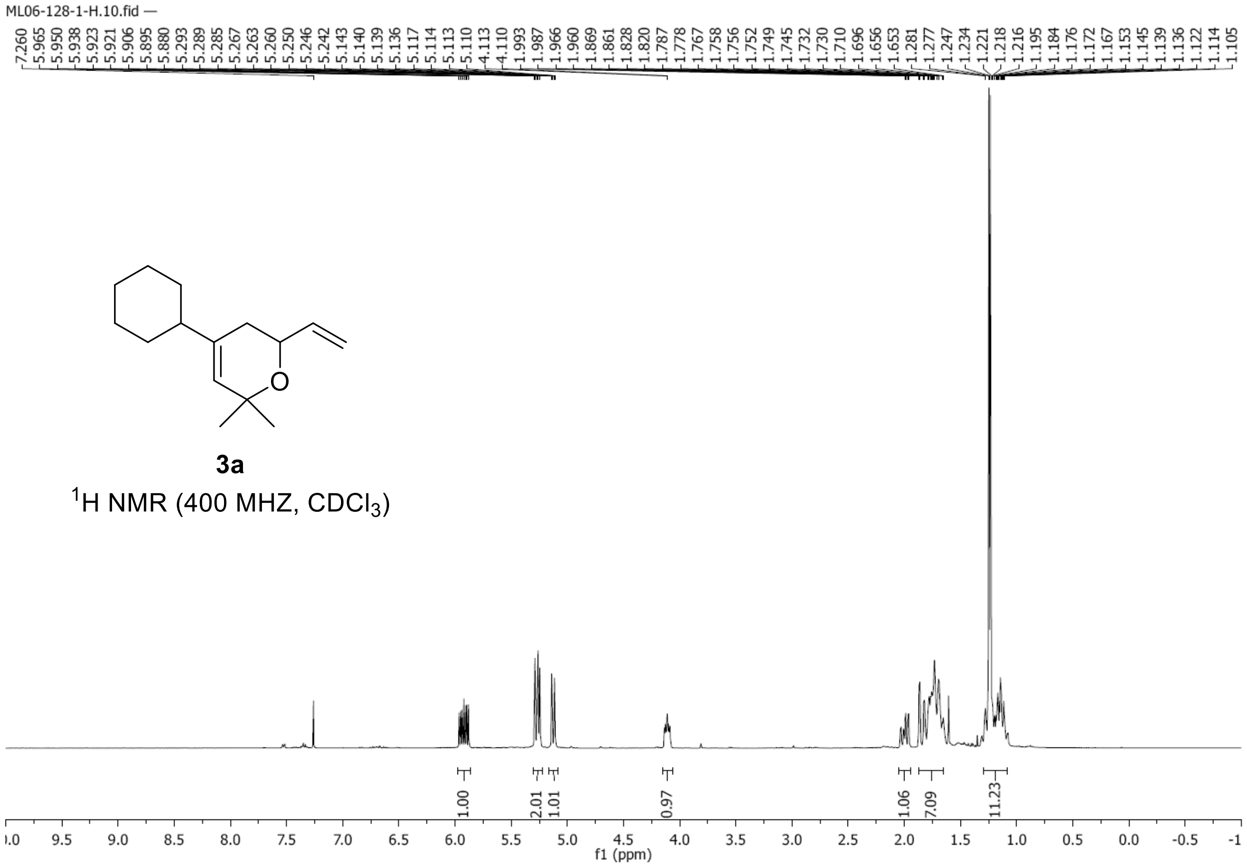
ML06-128-1-H.10.fid —
7.260 5.965 5.950 5.938 5.923 5.921 5.906 5.895 5.880 5.293 5.289 5.285 5.267 5.263 5.260 5.250 5.246 5.242 5.143 5.140 5.139 5.136 5.117 5.114 5.113 5.110 4.113 4.110 1.993 1.987 1.966 1.960 1.869 1.861 1.828 1.820 1.787 1.778 1.767 1.758 1.756 1.752 1.749 1.745 1.732 1.730 1.710 1.696 1.656 1.653 1.281 1.277 1.247 1.234 1.221 1.218 1.216 1.195 1.184 1.176 1.167 1.153 1.145 1.139 1.136 1.122 1.114 1.105
O
3a
^{1}H NMR (400 MHZ, CDCl$_3$)
1.00 2.01 1.01 0.97 1.06 7.09 11.23
f1 (ppm)

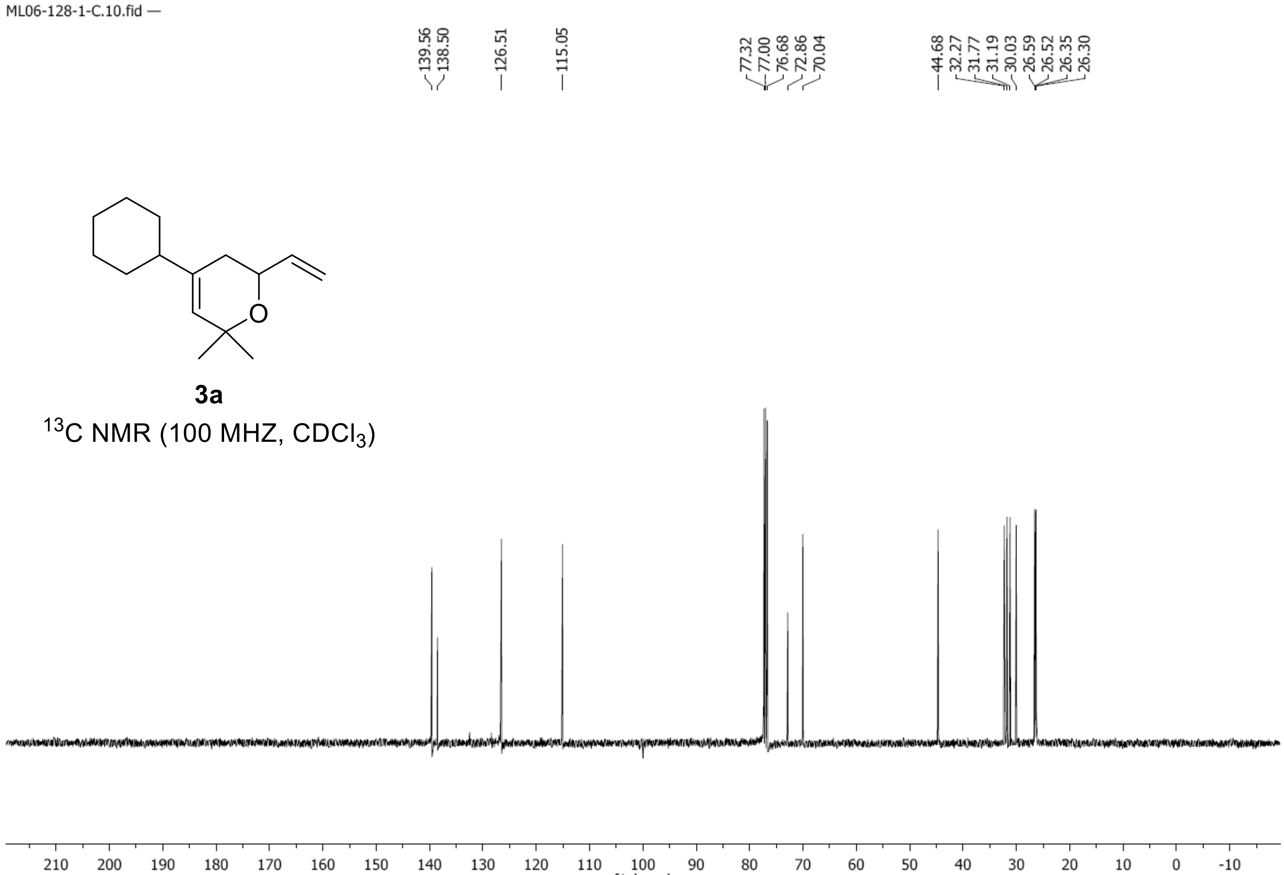
ML06-128-1-C.10.fid —
139.56 138.50 126.51 115.05 77.32 77.00 76.68 72.86 70.04 44.68 32.27 31.77 31.19 30.03 26.59 26.52 26.35 26.30
O
3a
^{13}C NMR (100 MHZ, CDCl$_3$)
f1 (ppm)

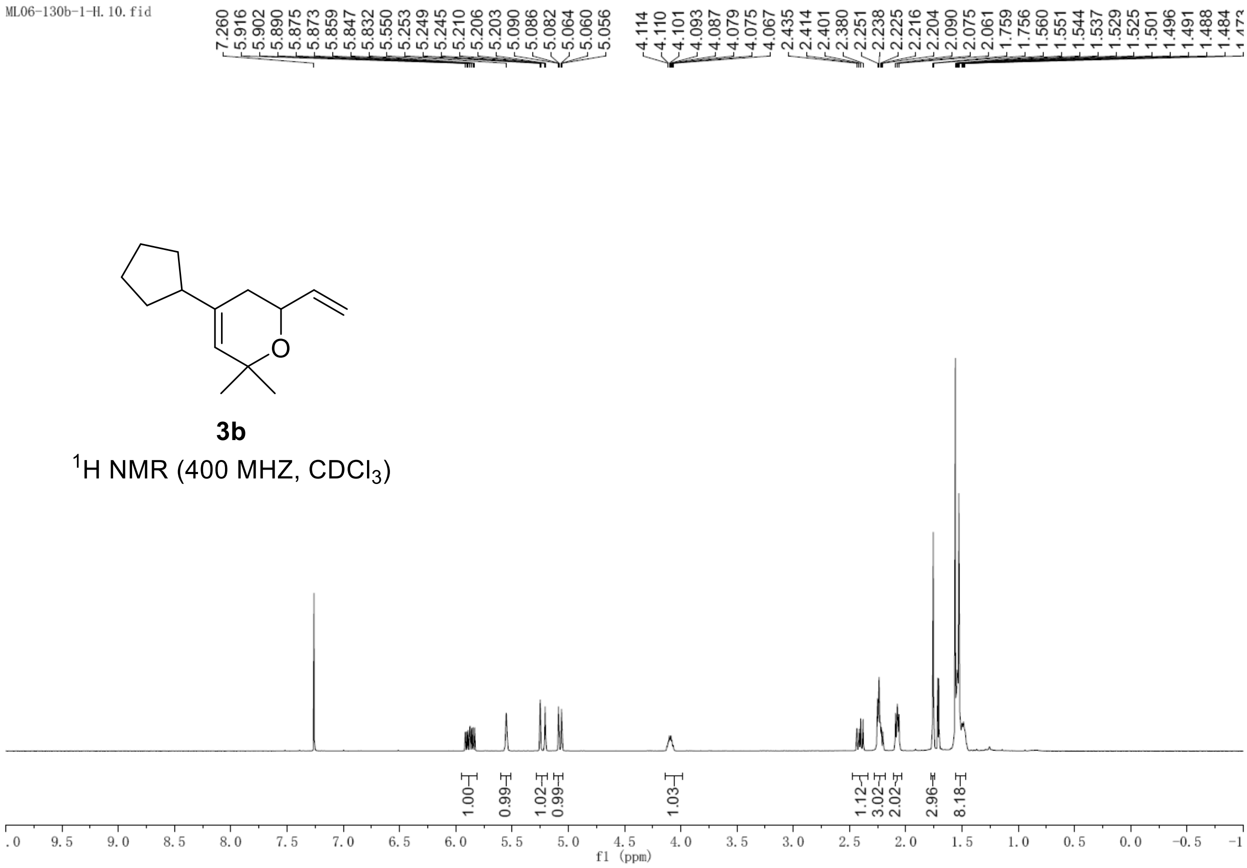

3b

^{1}H NMR (400 MHZ, $CDCl_3$)

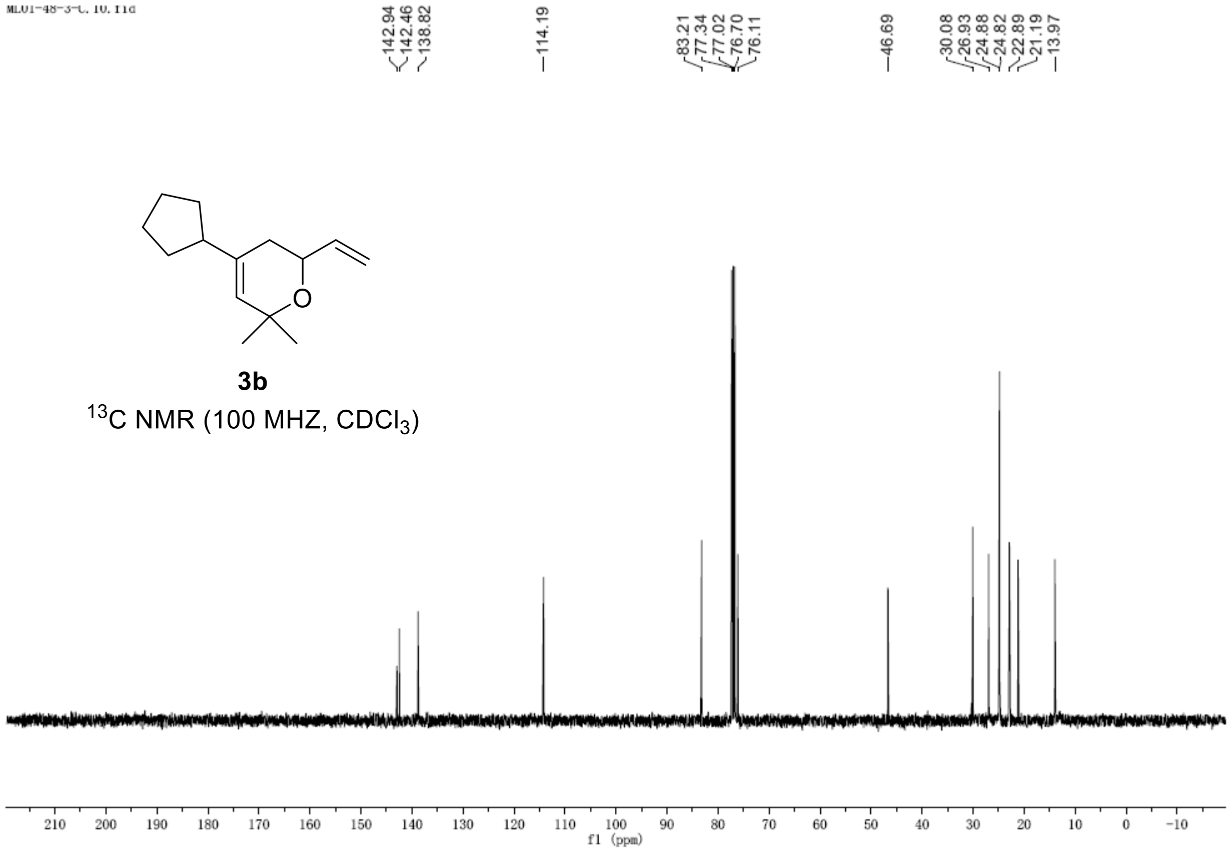

3b

^{13}C NMR (100 MHZ, $CDCl_3$)

3c

^{1}H NMR (400 MHZ, $CDCl_3$)

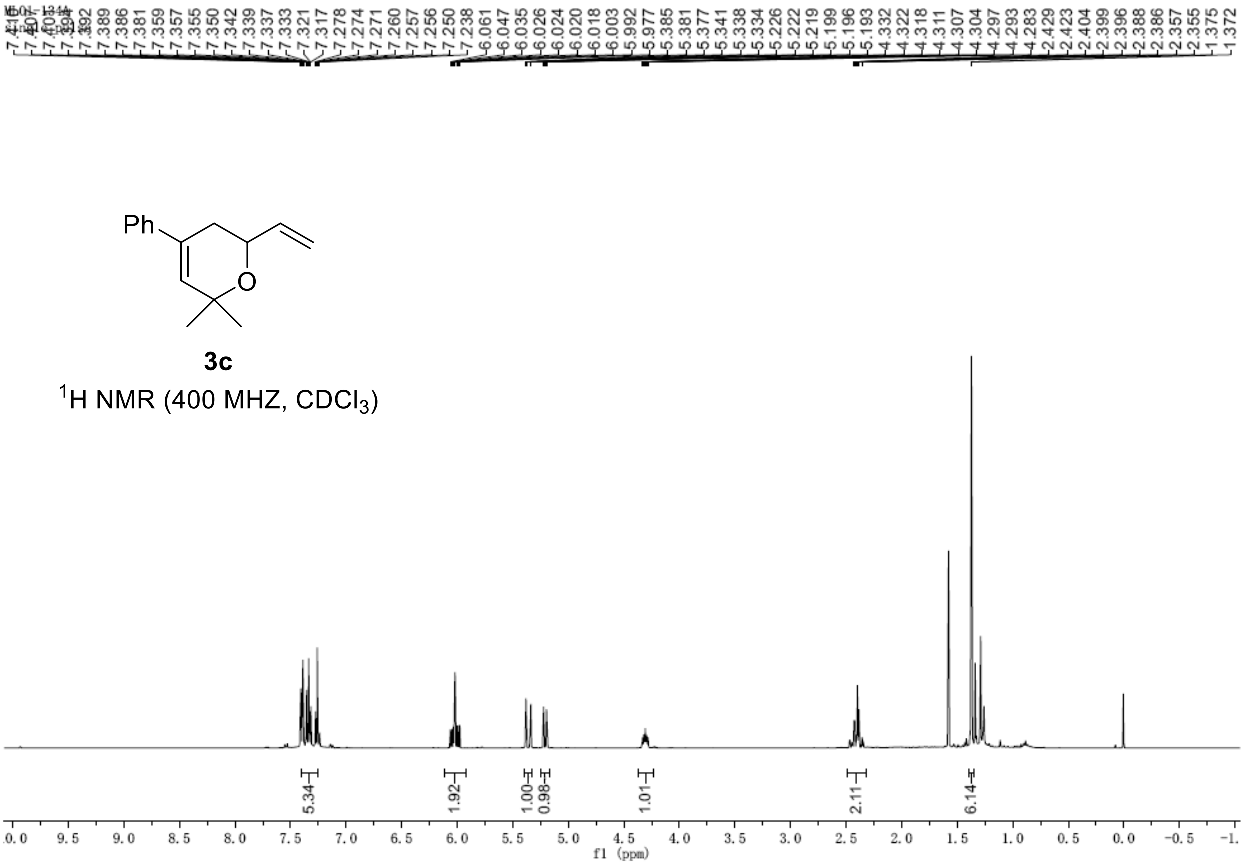

3c

^{13}C NMR (100 MHZ, $CDCl_3$)

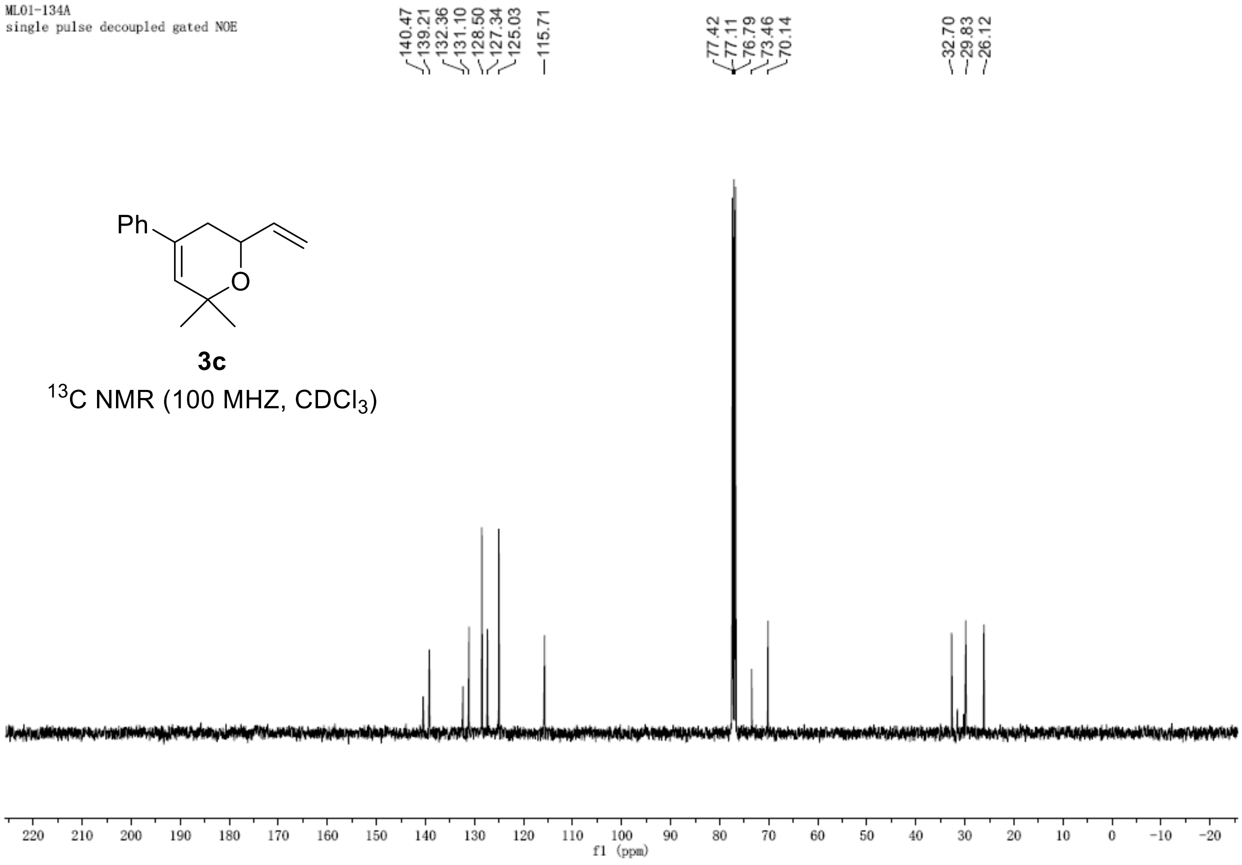

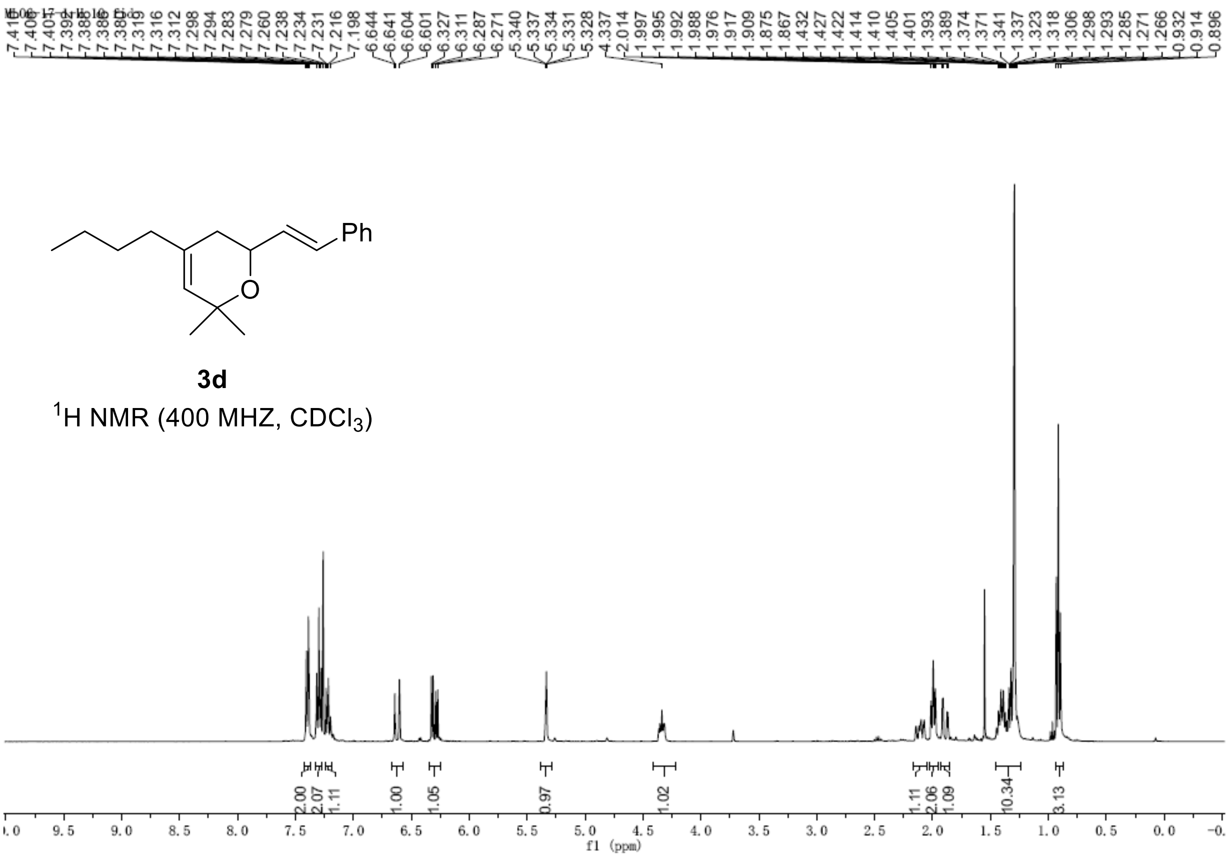
7.416
7.403
7.399
7.388
7.386
7.319
7.316
7.312
7.298
7.294
7.283
7.279
7.260
7.238
7.234
7.231
7.216
7.198
6.644
6.641
6.604
6.601
6.327
6.311
6.287
6.271
5.340
5.337
5.334
5.331
5.328
4.337
2.014
1.997
1.995
1.992
1.988
1.976
1.917
1.909
1.875
1.867
1.432
1.427
1.422
1.414
1.410
1.405
1.401
1.393
1.389
1.374
1.371
1.341
1.337
1.323
1.318
1.306
1.298
1.293
1.285
1.271
1.266
0.932
0.914
0.896
Ph
O
3d
1H NMR (400 MHZ, CDCl3)
2.00
2.07
1.11
1.00
1.05
0.97
1.02
1.11
2.06
1.09
10.34
3.13
f1 (ppm)

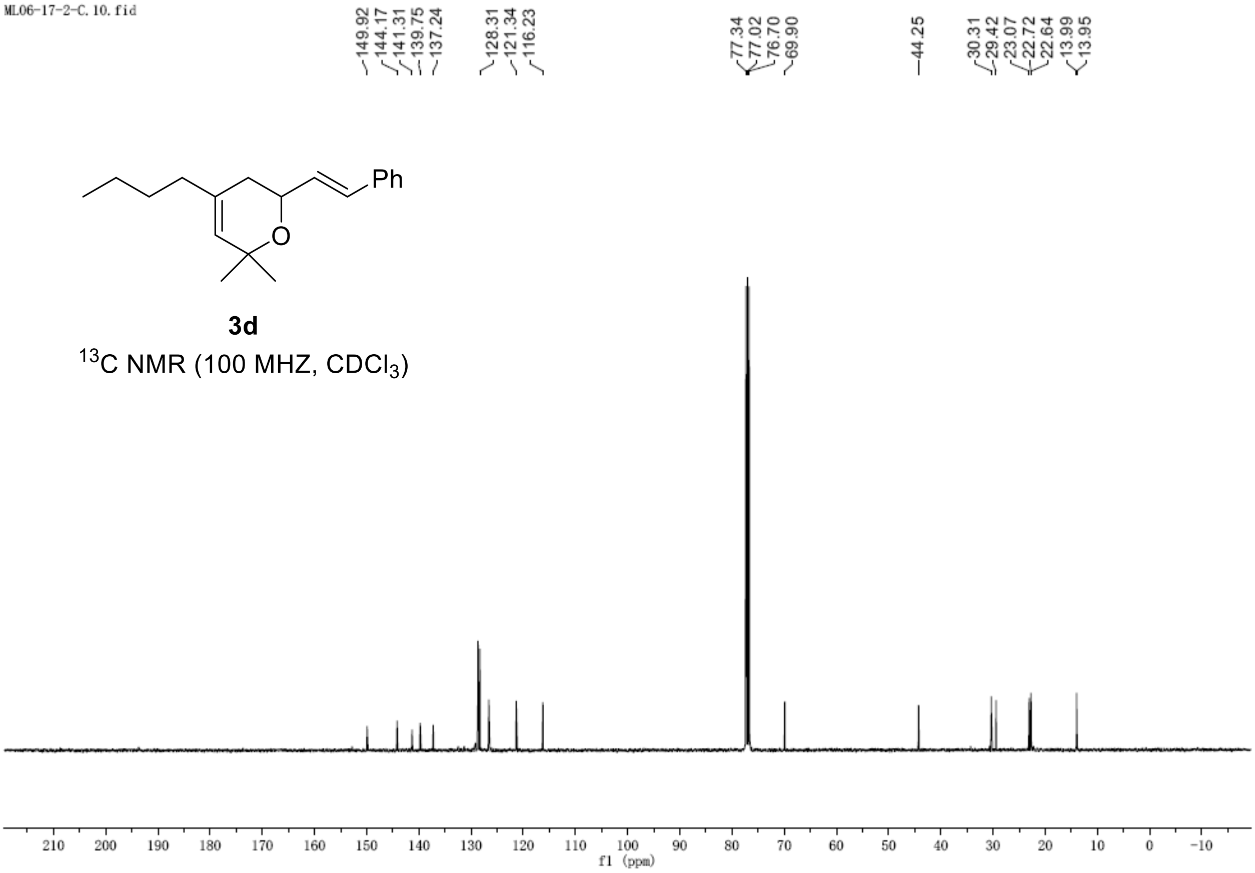
ML06-17-2-C.10.fid
149.92
144.17
141.31
139.75
137.24
128.31
121.34
116.23
77.34
77.02
76.70
69.90
44.25
30.31
29.42
23.07
22.72
22.64
13.99
13.95
Ph
O
3d
13C NMR (100 MHZ, CDCl3)
f1 (ppm)

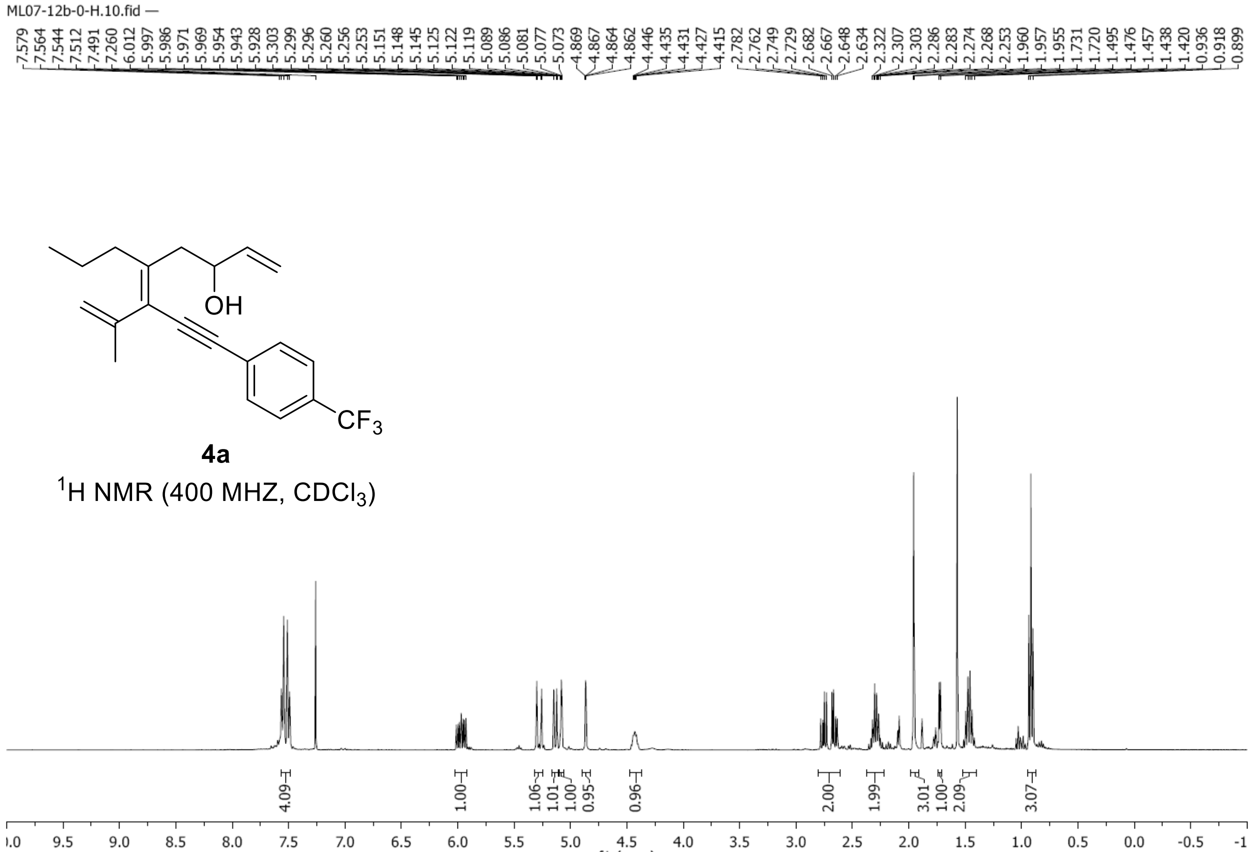
ML07-12b-0-H.10.fid —
7.579 7.564 7.544 7.512 7.491 7.260 6.012 5.997 5.986 5.971 5.969 5.954 5.943 5.928 5.303 5.299 5.296 5.260 5.256 5.253 5.151 5.148 5.145 5.125 5.122 5.119 5.089 5.086 5.081 5.077 5.073 4.869 4.867 4.864 4.862 4.446 4.435 4.431 4.427 4.415 2.782 2.762 2.749 2.729 2.682 2.667 2.648 2.634 2.322 2.307 2.303 2.286 2.283 2.274 2.268 2.253 1.960 1.957 1.955 1.731 1.720 1.495 1.476 1.457 1.438 1.420 0.936 0.918 0.899
OH
CF3
4a
1H NMR (400 MHZ, CDCl3)
4.09 1.00 1.06 1.01 1.00 0.95 0.96 2.00 1.99 3.01 1.00 2.09 3.07
f1 (ppm)

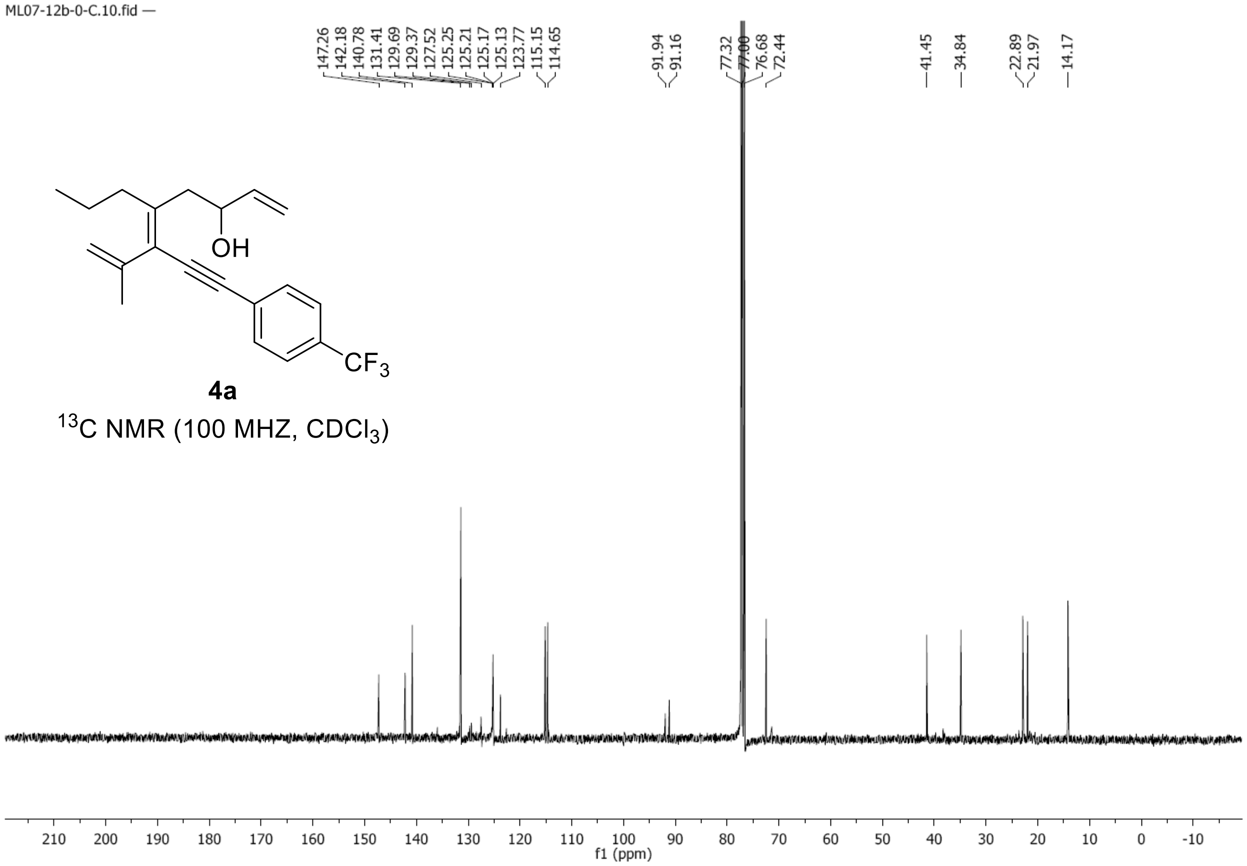
ML07-12b-0-C.10.fid —
147.26 142.18 140.78 131.41 129.69 129.37 127.52 125.25 125.21 125.17 125.13 123.77 115.15 114.65 91.94 91.16 77.32 77.00 76.68 72.44 41.45 34.84 22.89 21.97 14.17
OH
CF3
4a
13C NMR (100 MHZ, CDCl3)
f1 (ppm)

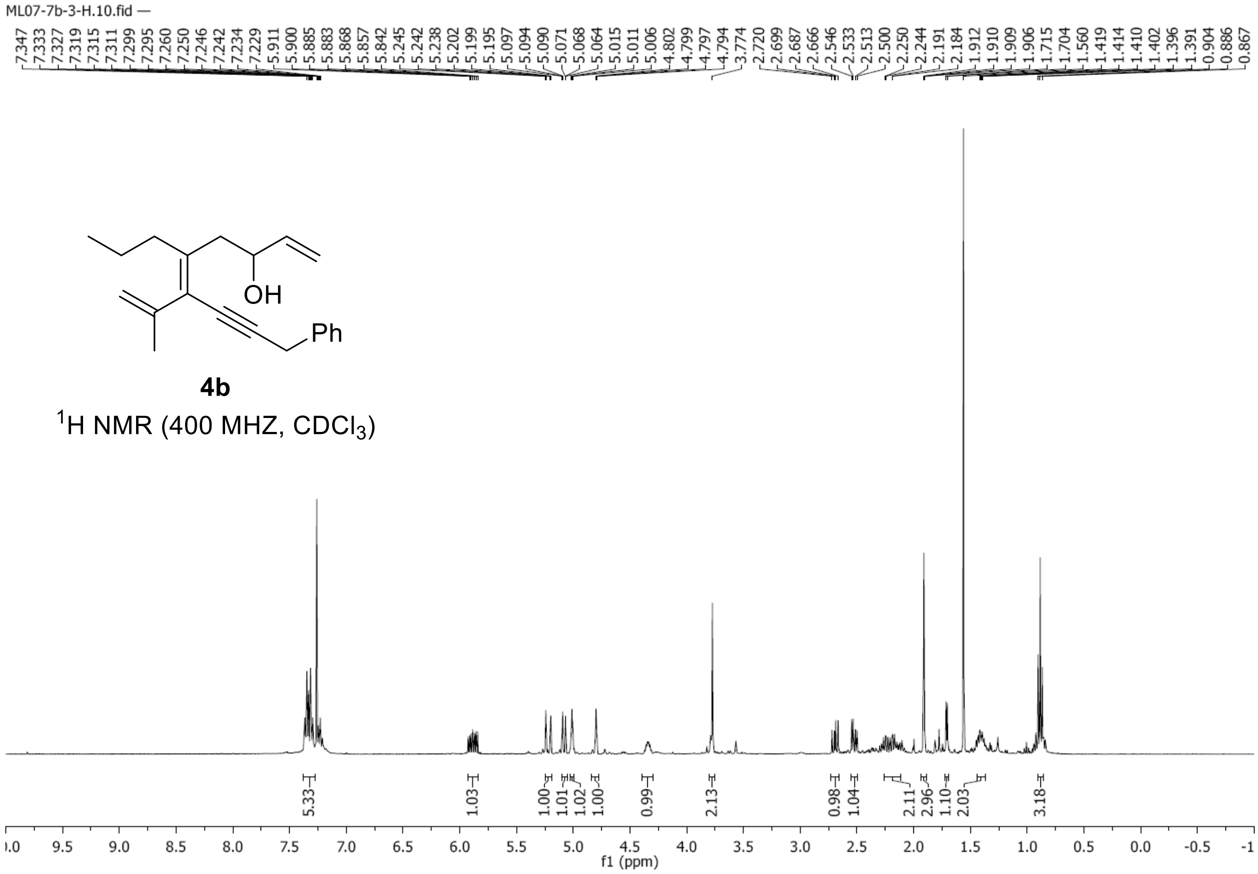

4b

^{1}H NMR (400 MHZ, $CDCl_3$)

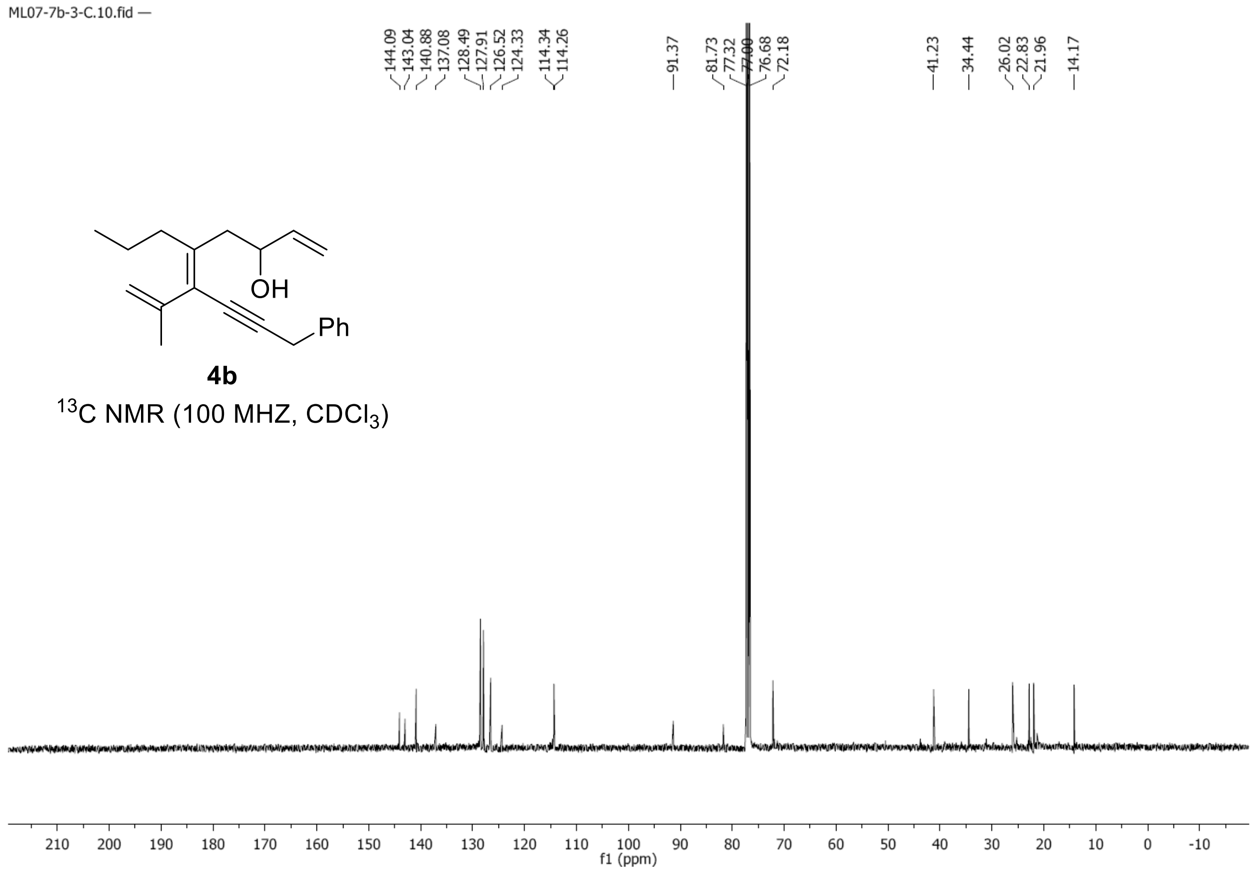

4b

^{13}C NMR (100 MHZ, $CDCl_3$)

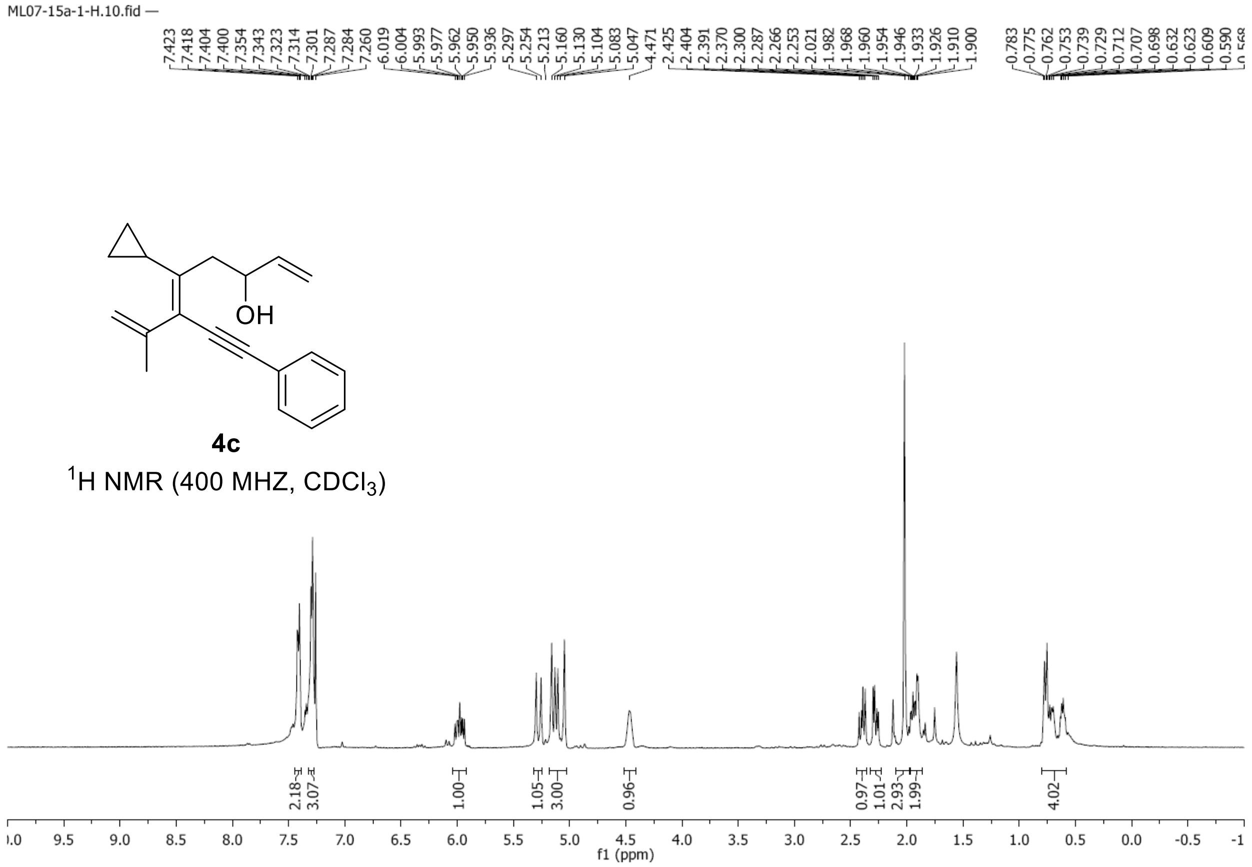
ML07-15a-1-H.10.fid —
7.423 7.418 7.404 7.400 7.354 7.343 7.323 7.314 7.301 7.287 7.284 7.260 6.019 6.004 5.993 5.977 5.962 5.950 5.936 5.297 5.254 5.213 5.160 5.130 5.104 5.083 5.047 4.471 2.425 2.404 2.391 2.370 2.300 2.287 2.266 2.253 2.021 1.982 1.968 1.960 1.954 1.946 1.933 1.926 1.910 1.900 0.783 0.775 0.762 0.753 0.739 0.729 0.712 0.707 0.698 0.632 0.623 0.609 0.590 [illegible]
OH
4c
1H NMR (400 MHZ, CDCl3)
2.18 3.07 1.00 1.05 3.00 0.96 0.97 1.01 2.93 1.99 4.02
f1 (ppm)

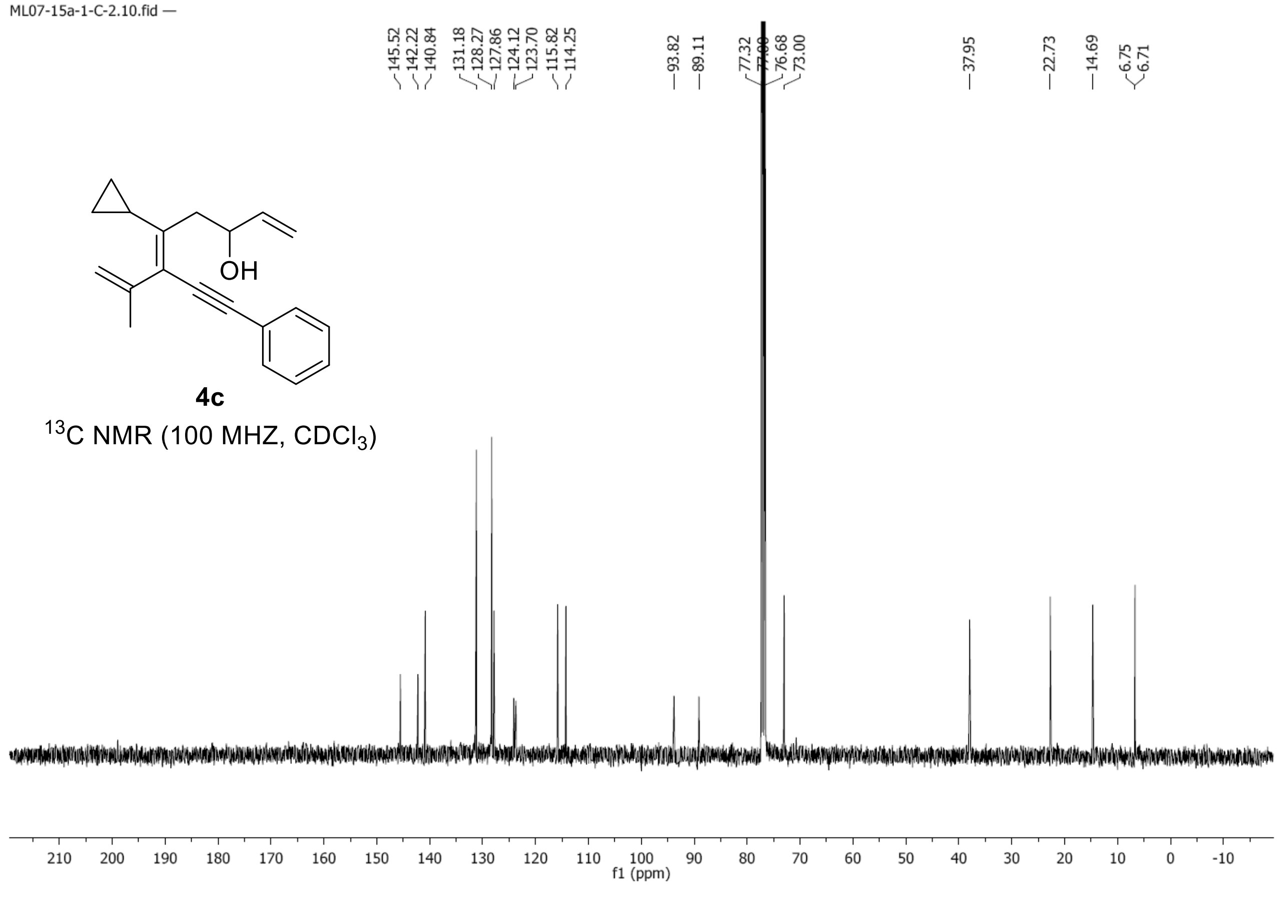
ML07-15a-1-C-2.10.fid —
145.52 142.22 140.84 131.18 128.27 127.86 124.12 123.70 115.82 114.25 93.82 89.11 77.32 77.00 76.68 73.00 37.95 22.73 14.69 6.75 6.71
OH
4c
13C NMR (100 MHZ, CDCl3)
f1 (ppm)

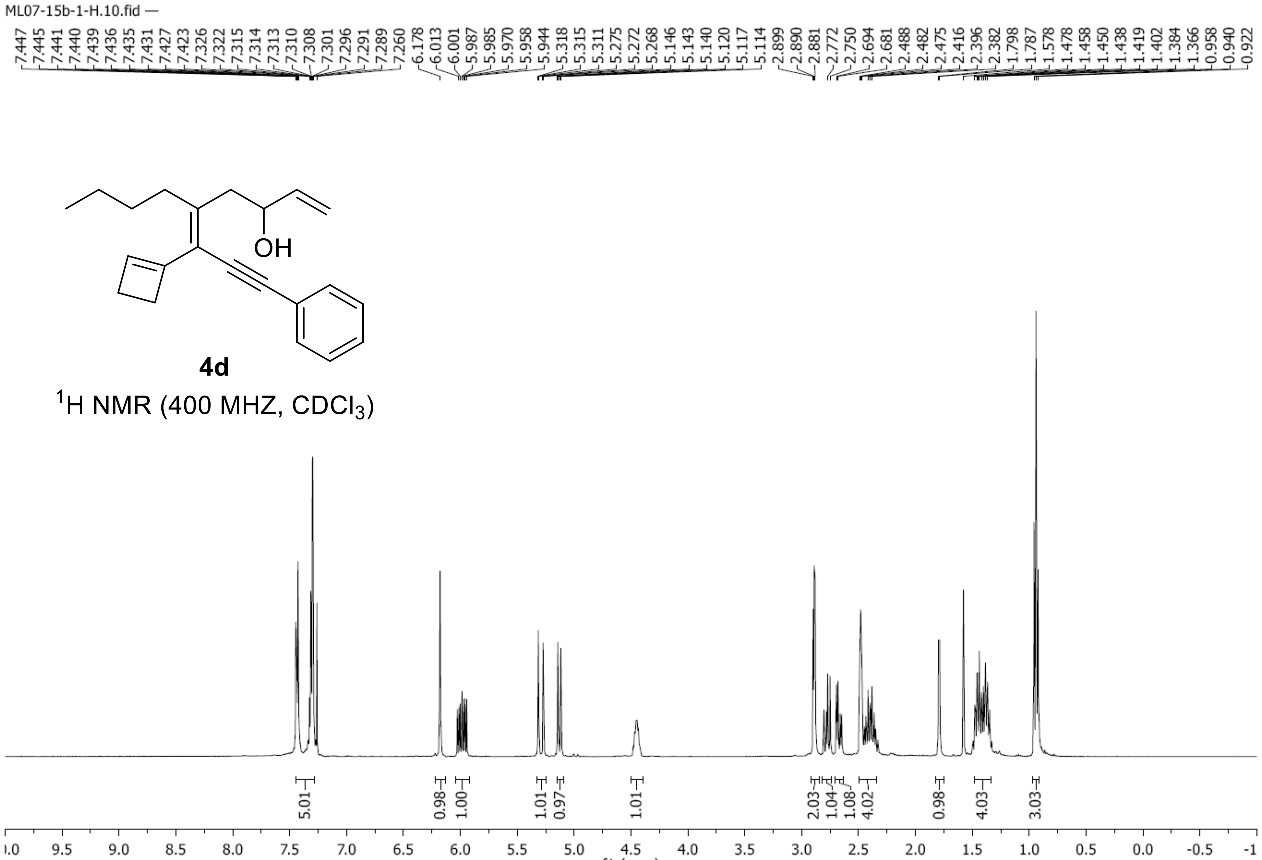
ML07-15b-1-H.10.fid —
7.447 7.445 7.441 7.440 7.439 7.436 7.435 7.431 7.427 7.423 7.326 7.322 7.315 7.314 7.313 7.310 7.308 7.301 7.296 7.291 7.289 7.260 6.178 6.013 6.001 5.987 5.985 5.970 5.958 5.944 5.318 5.315 5.311 5.275 5.272 5.268 5.146 5.143 5.140 5.120 5.117 5.114 2.899 2.890 2.881 2.772 2.750 2.694 2.681 2.488 2.482 2.475 2.416 2.396 2.382 1.798 1.787 1.578 1.478 1.458 1.450 1.438 1.419 1.402 1.384 1.366 0.958 0.940 0.922
OH
4d
1H NMR (400 MHZ, CDCl3)
5.01 0.98 1.00 1.01 0.97 1.01 2.03 1.04 1.08 4.02 0.98 4.03 3.03
f1 (ppm)

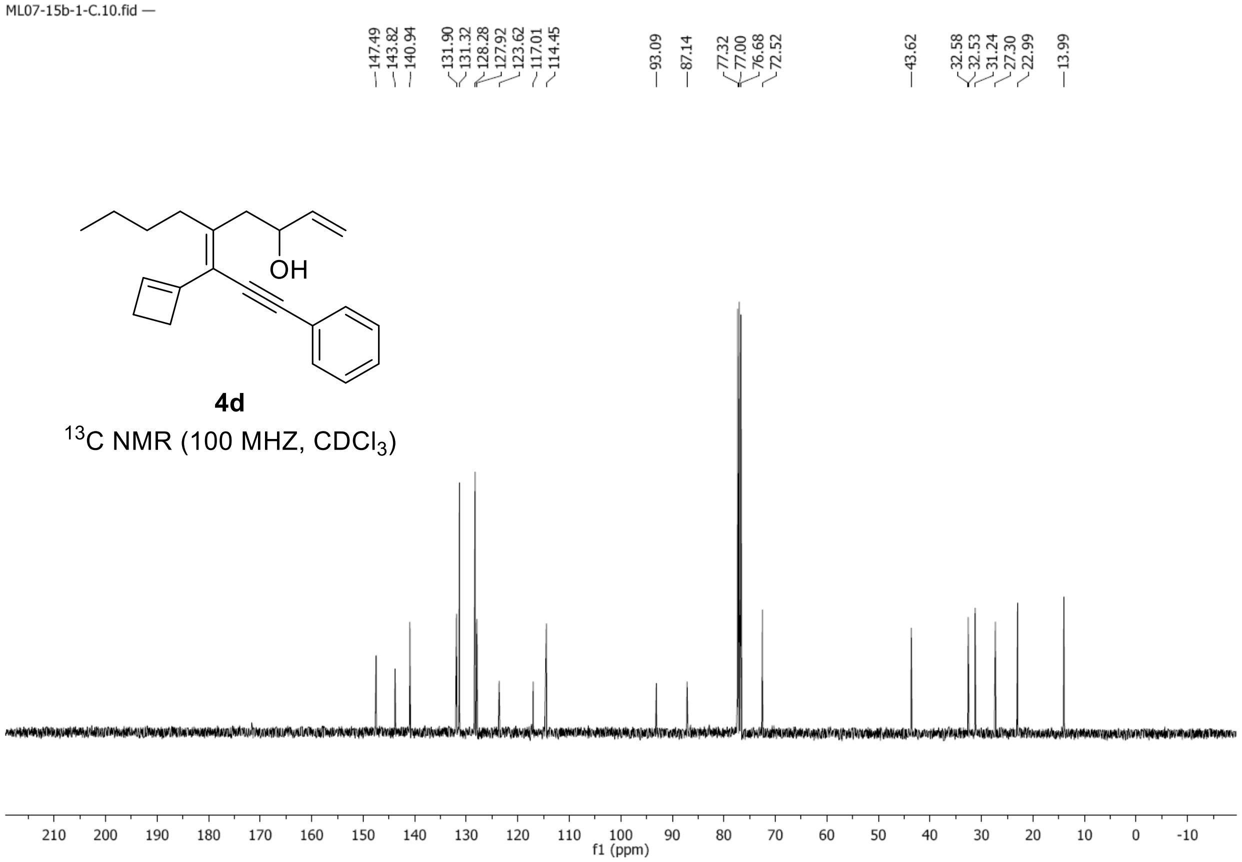
ML07-15b-1-C.10.fid —
147.49 143.82 140.94 131.90 131.32 128.28 127.92 123.62 117.01 114.45 93.09 87.14 77.32 77.00 76.68 72.52 43.62 32.58 32.53 31.24 27.30 22.99 13.99
OH
4d
13C NMR (100 MHZ, CDCl3)
f1 (ppm)

4e

^{1}H NMR (400 MHZ, $CDCl_3$)

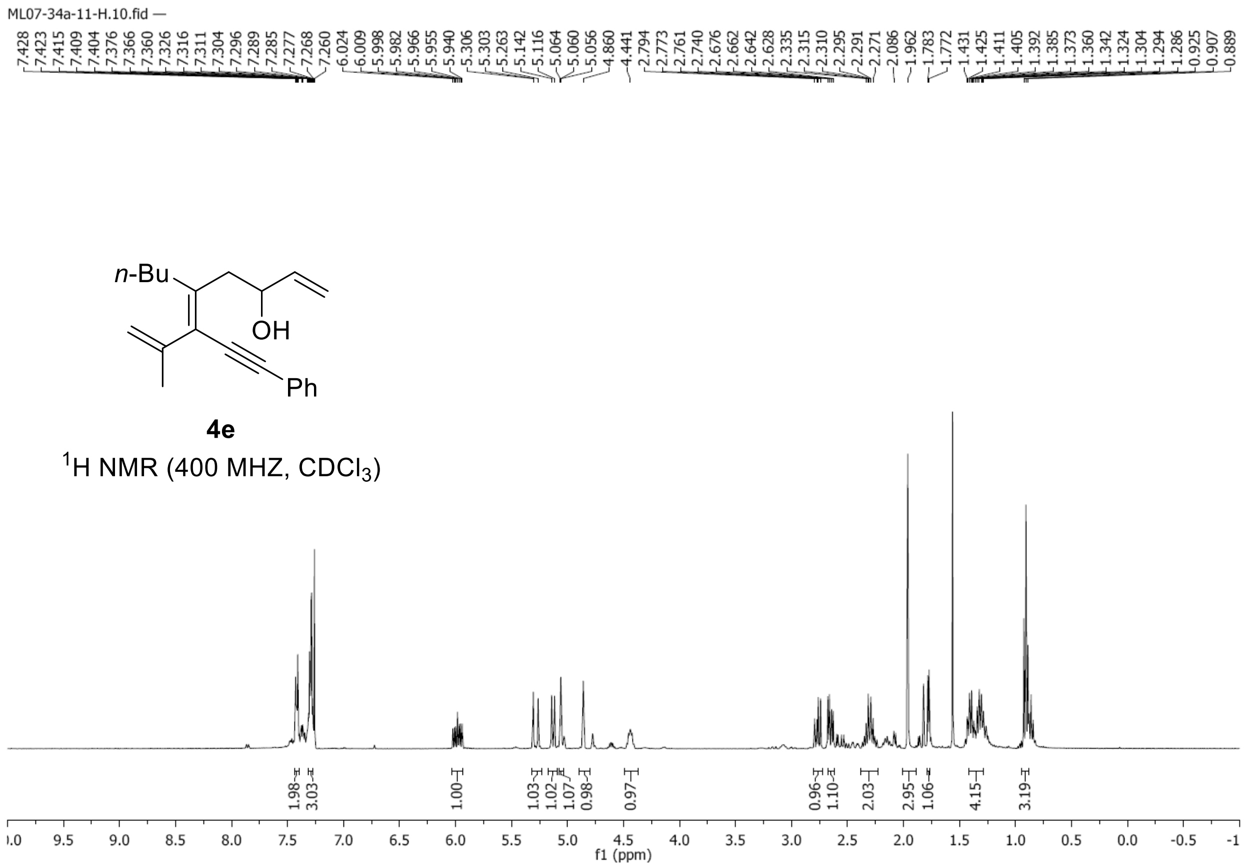

4e

^{13}C NMR (100 MHZ, $CDCl_3$)

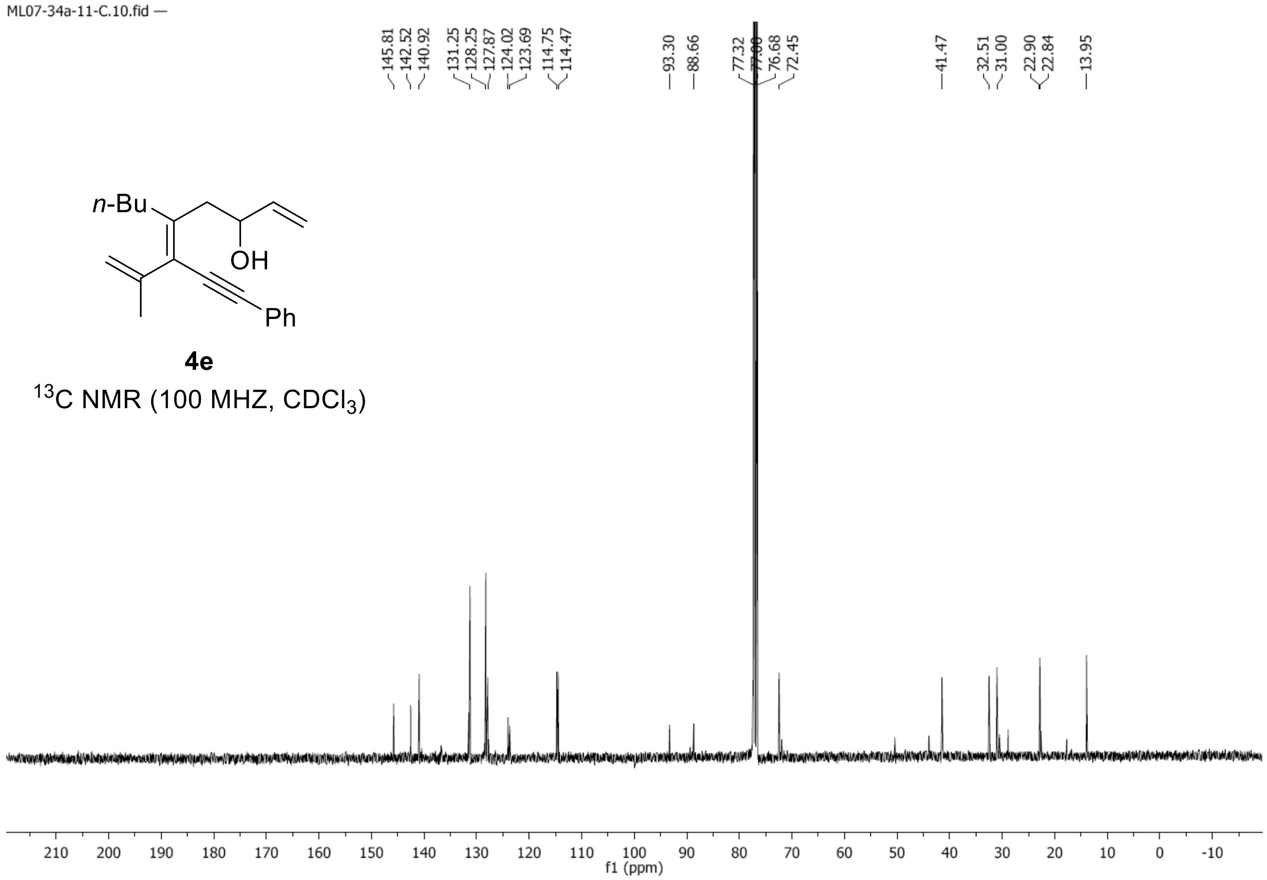

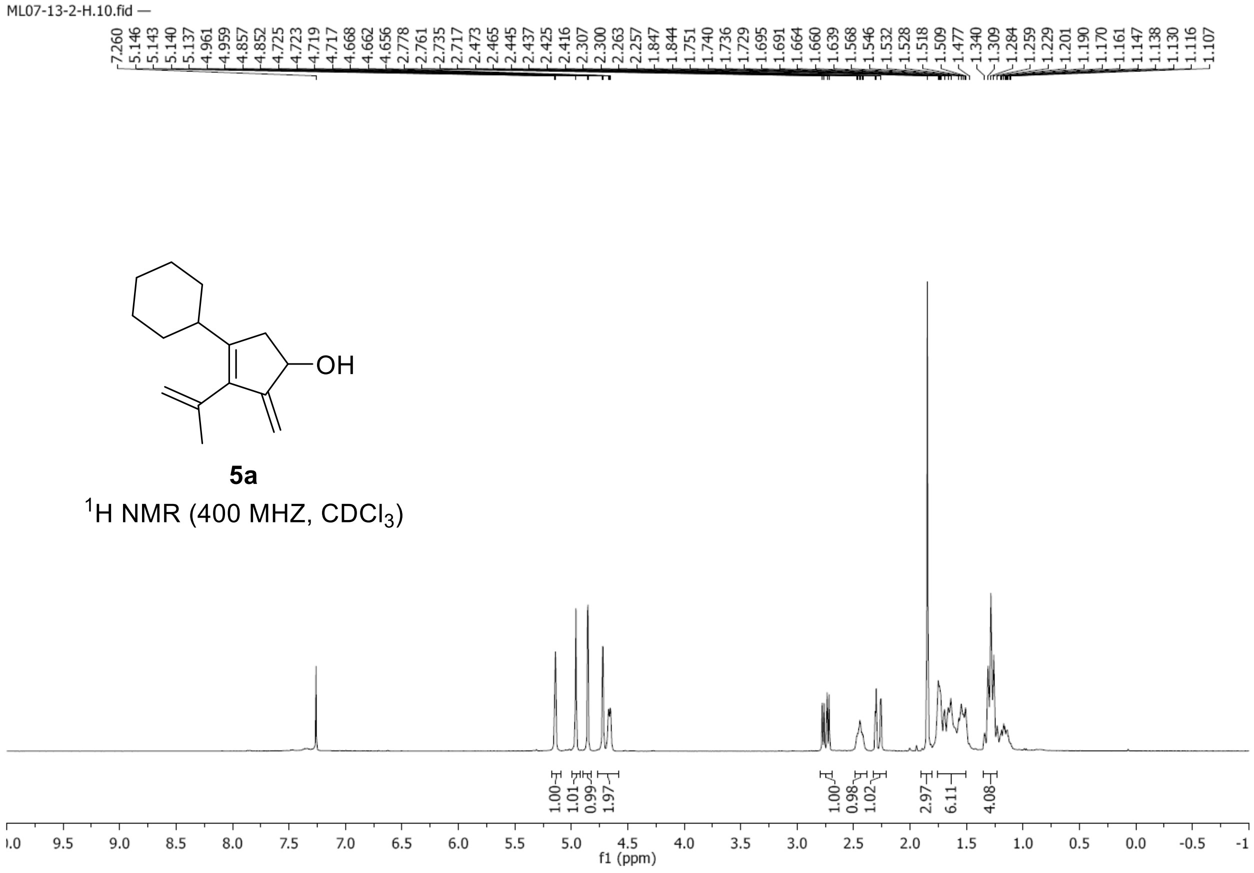
ML07-13-2-H.10.fid
5a
^{1}H NMR (400 MHZ, CDCl$_3$)
f1 (ppm)

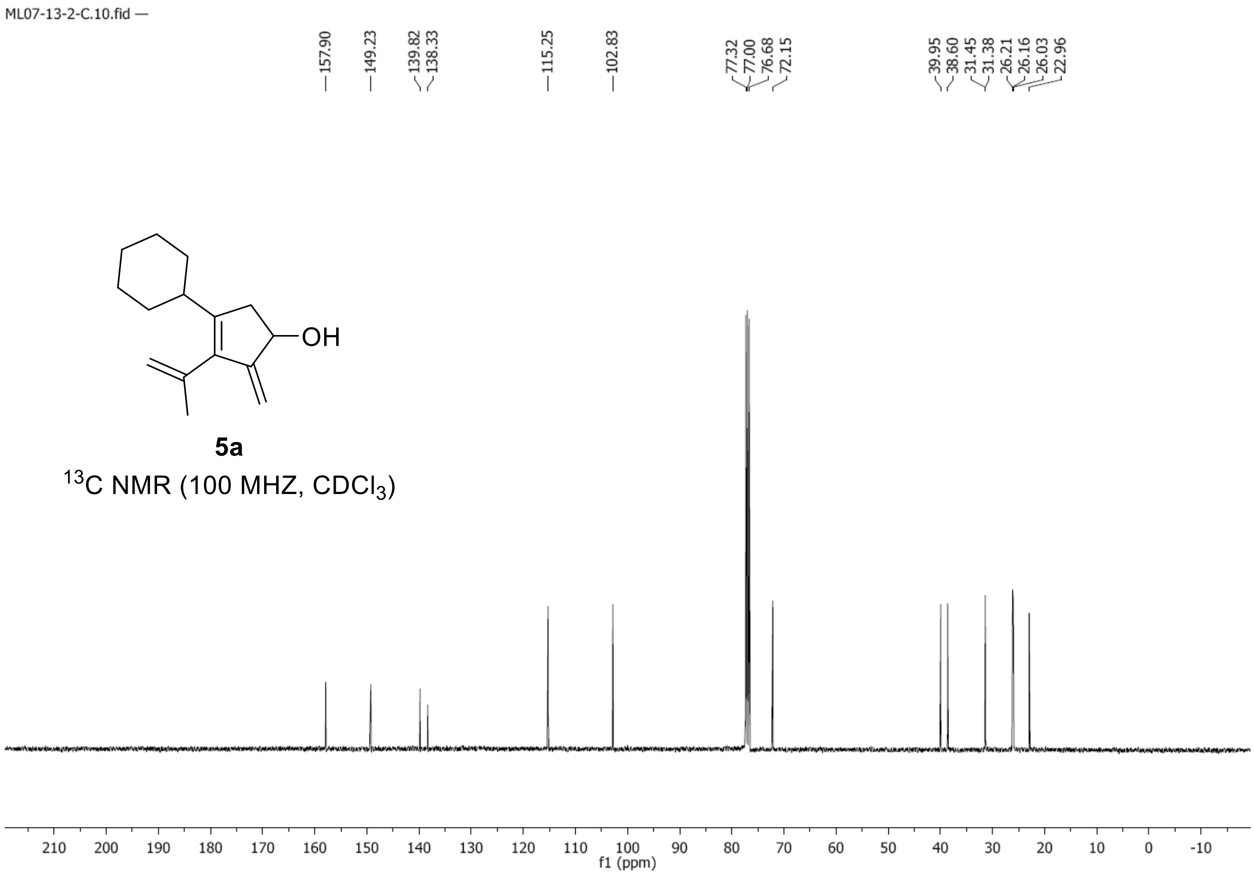
ML07-13-2-C.10.fid
157.90
149.23
139.82
138.33
115.25
102.83
77.32
77.00
76.68
72.15
39.95
38.60
31.45
31.38
26.21
26.16
26.03
22.96
5a
^{13}C NMR (100 MHZ, CDCl$_3$)
f1 (ppm)

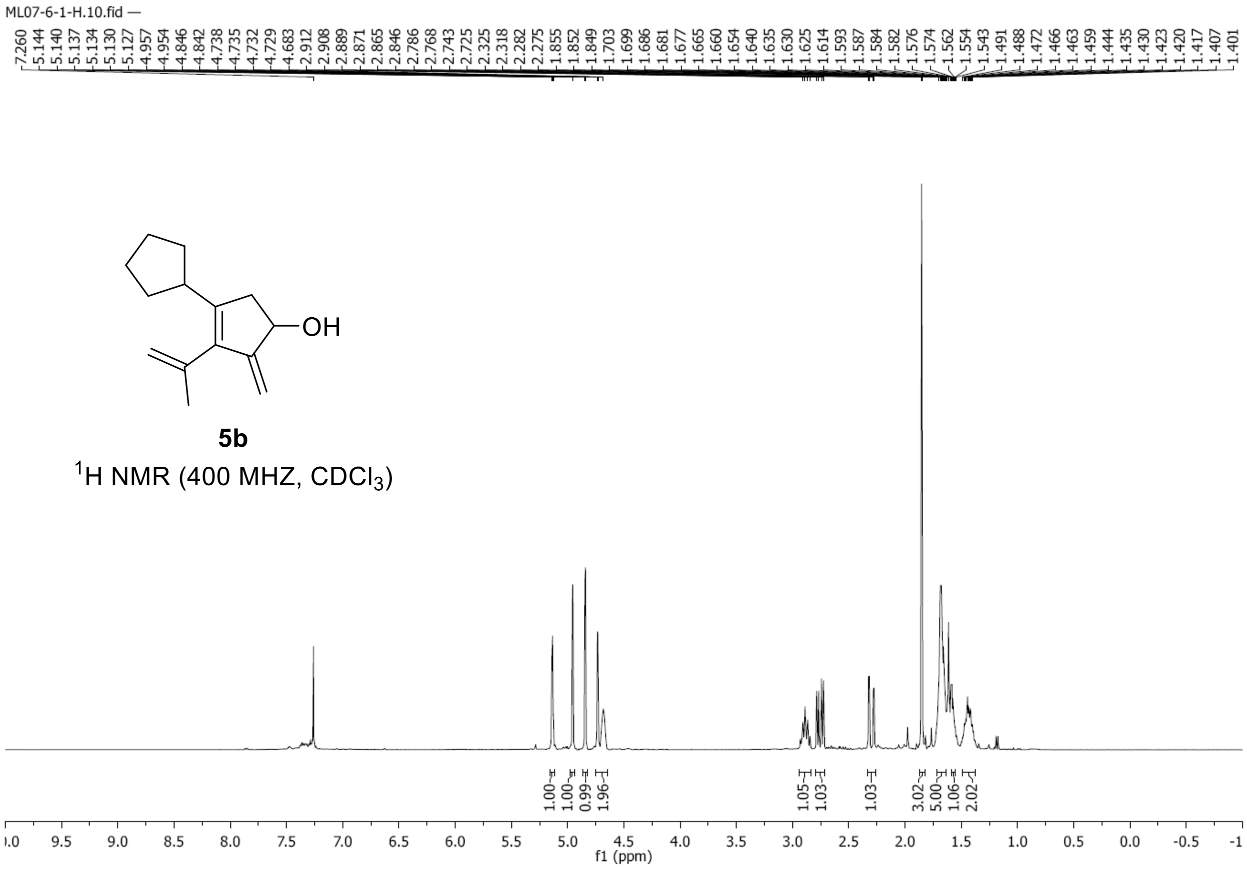
ML07-6-1-H.10.fid
7.260 5.144 5.140 5.137 5.134 5.130 5.127 4.957 4.954 4.846 4.842 4.738 4.735 4.732 4.729 4.683 2.912 2.908 2.889 2.871 2.865 2.846 2.786 2.768 2.743 2.725 2.325 2.318 2.282 2.275 1.855 1.852 1.849 1.703 1.699 1.686 1.681 1.677 1.665 1.660 1.654 1.640 1.635 1.630 1.625 1.614 1.593 1.587 1.584 1.582 1.576 1.574 1.562 1.554 1.543 1.491 1.488 1.472 1.466 1.463 1.459 1.444 1.435 1.430 1.423 1.420 1.417 1.407 1.401
OH
5b
^{1}H NMR (400 MHZ, CDCl$_3$)
1.00 1.00 0.99 1.96 1.05 1.03 1.03 3.02 5.00 1.06 2.02
f1 (ppm)

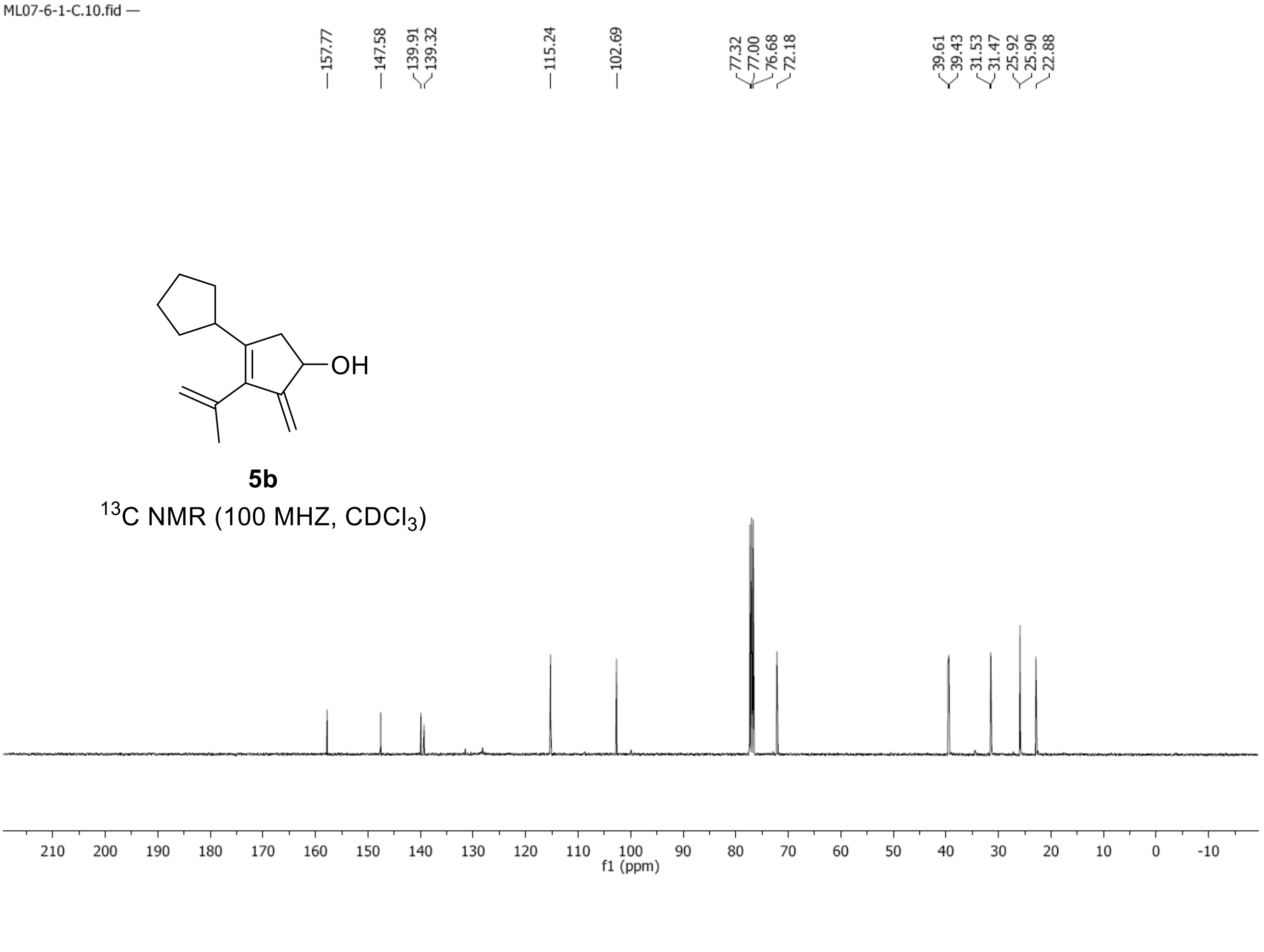
ML07-6-1-C.10.fid
157.77 147.58 139.91 139.32 115.24 102.69 77.32 77.00 76.68 72.18 39.61 39.43 31.53 31.47 25.92 25.90 22.88
OH
5b
^{13}C NMR (100 MHZ, CDCl$_3$)
f1 (ppm)

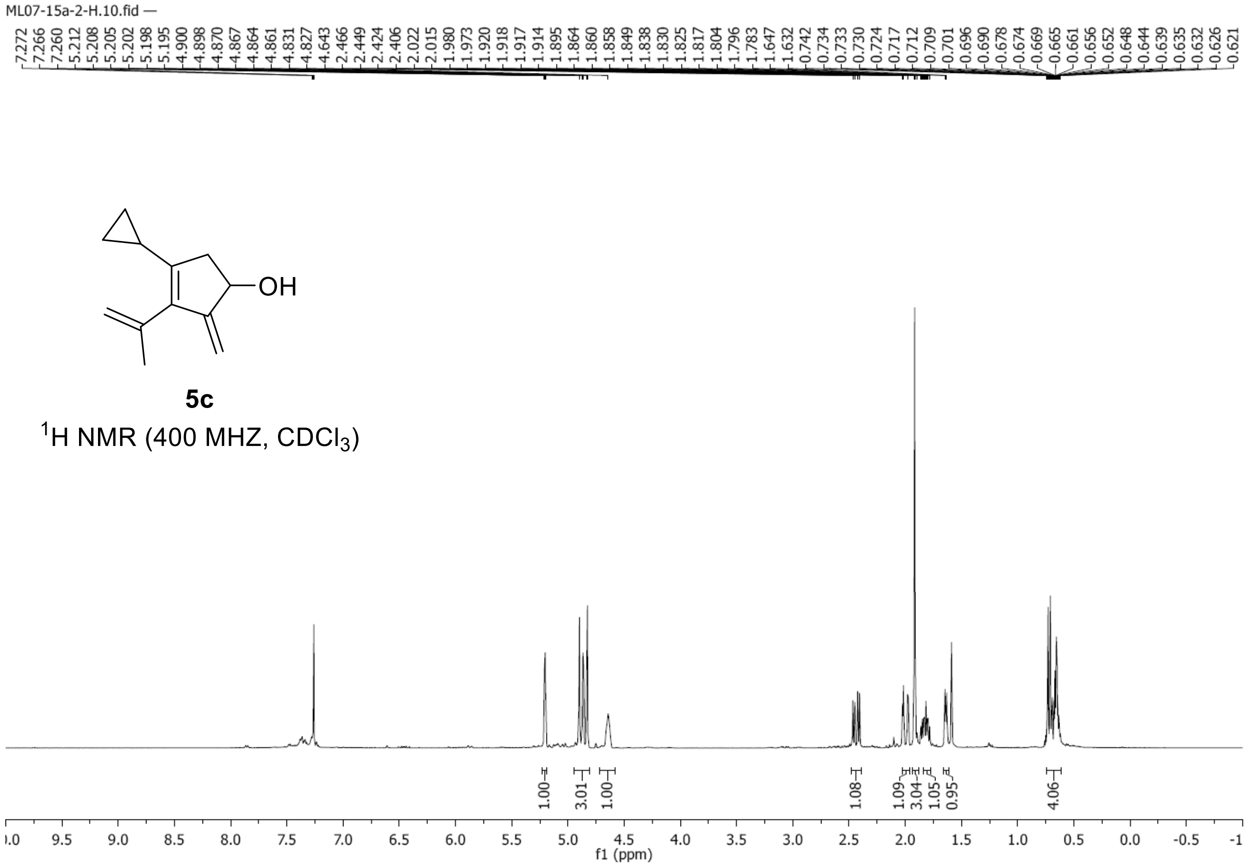
ML07-15a-2-H.10.fid
7.272 7.266 7.260 5.212 5.208 5.205 5.202 5.198 5.195 4.900 4.898 4.870 4.867 4.864 4.861 4.831 4.827 4.643 2.466 2.449 2.424 2.406 2.022 2.015 1.980 1.973 1.920 1.918 1.917 1.914 1.895 1.864 1.860 1.858 1.849 1.838 1.830 1.825 1.817 1.804 1.796 1.783 1.647 1.632 0.742 0.734 0.733 0.730 0.724 0.717 0.712 0.709 0.701 0.696 0.690 0.678 0.674 0.669 0.665 0.661 0.656 0.652 0.648 0.644 0.639 0.635 0.632 0.626 0.621
OH
5c
1H NMR (400 MHZ, CDCl3)
1.00 3.01 1.00 1.08 1.09 3.04 1.05 0.95 4.06
f1 (ppm)

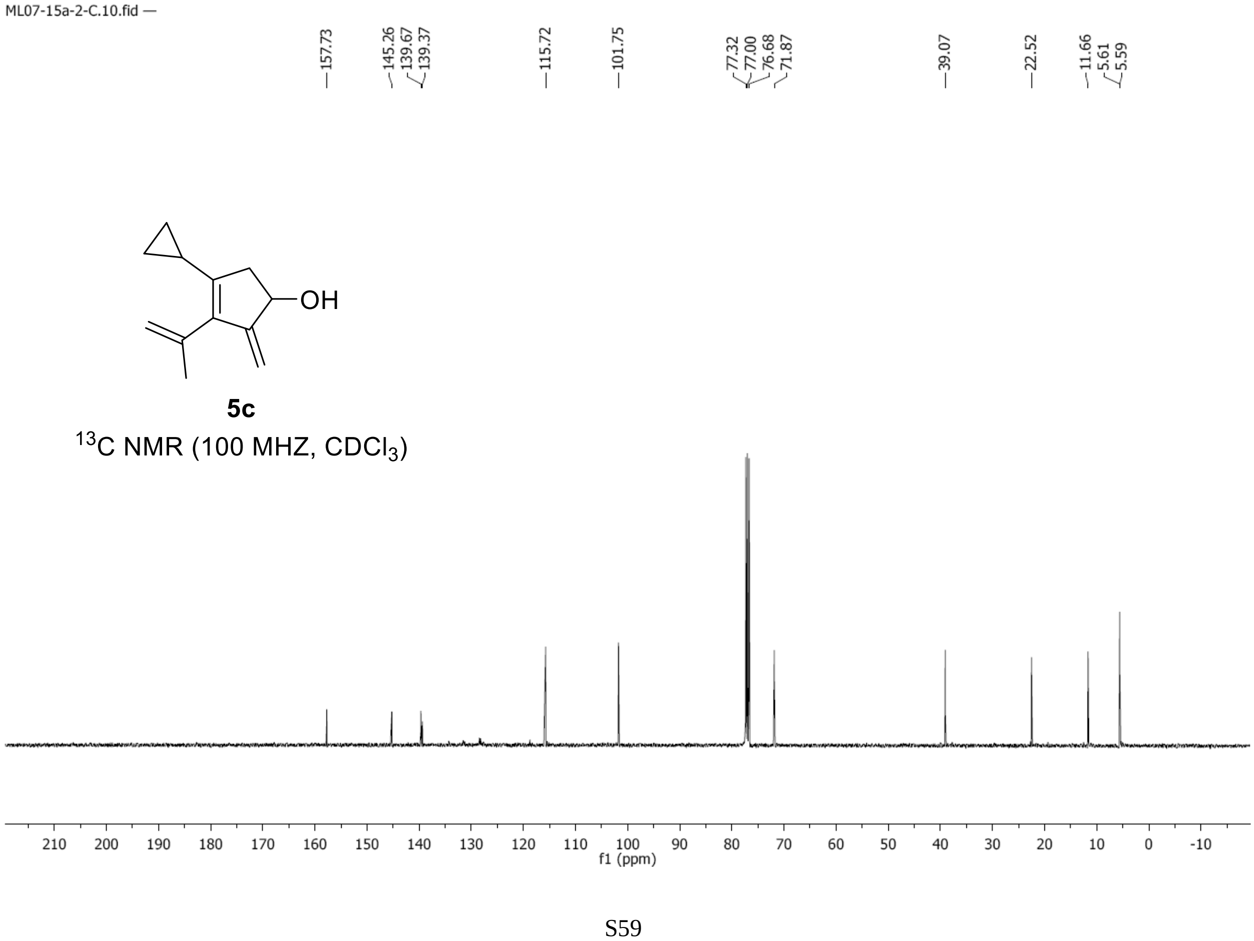
ML07-15a-2-C.10.fid
157.73 145.26 139.67 139.37 115.72 101.75 77.32 77.00 76.68 71.87 39.07 22.52 11.66 5.61 5.59
OH
5c
13C NMR (100 MHZ, CDCl3)
f1 (ppm)

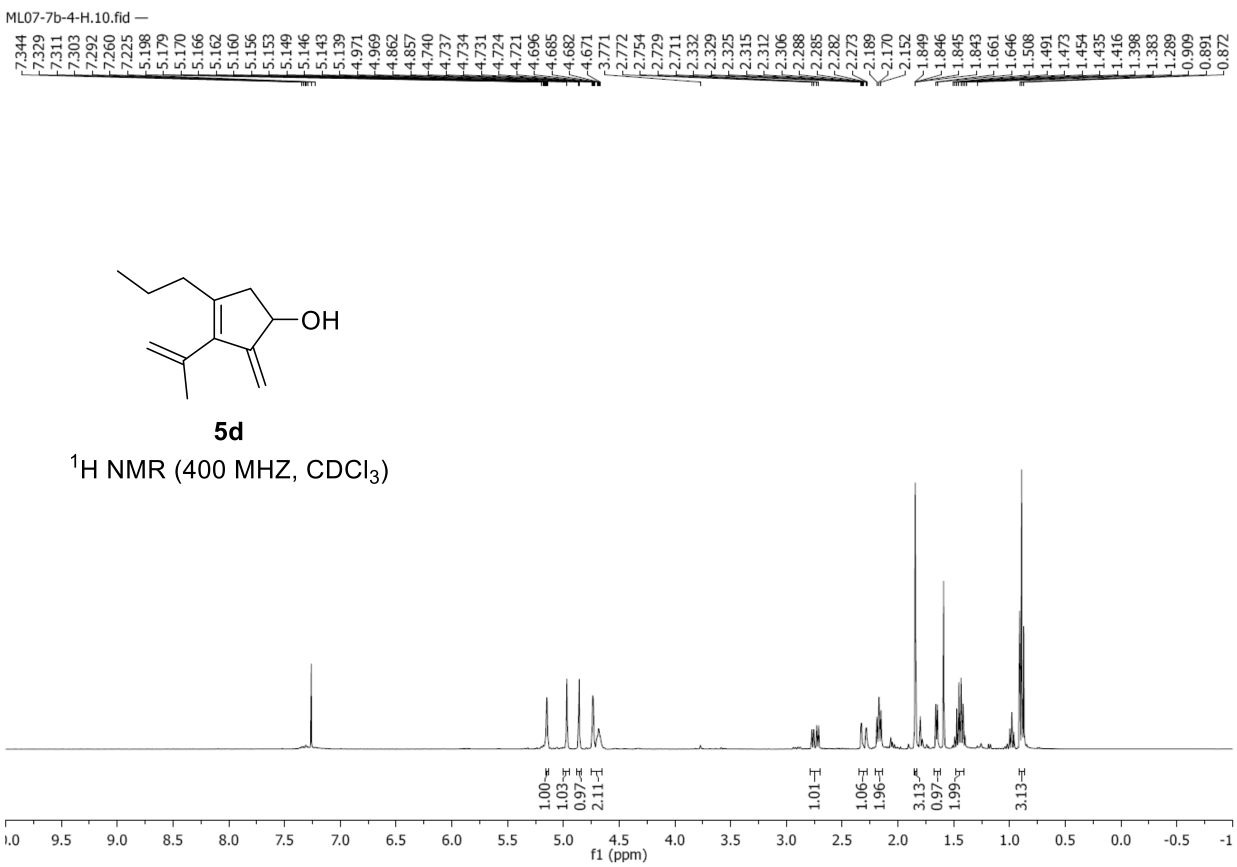
ML07-7b-4-H.10.fid
7.344 7.329 7.311 7.303 7.292 7.260 7.225 5.198 5.179 5.170 5.166 5.162 5.160 5.156 5.153 5.149 5.146 5.143 5.139 4.971 4.969 4.862 4.857 4.740 4.737 4.734 4.731 4.724 4.721 4.696 4.685 4.682 4.671 3.771 2.772 2.754 2.729 2.711 2.332 2.329 2.325 2.315 2.312 2.306 2.288 2.285 2.282 2.273 2.189 2.170 2.152 1.849 1.846 1.845 1.843 1.661 1.646 1.508 1.491 1.473 1.454 1.435 1.416 1.398 1.383 1.289 0.909 0.891 0.872
OH
5d
¹H NMR (400 MHZ, CDCl₃)
1.00 1.03 0.97 2.11 1.01 1.06 1.96 3.13 0.97 1.99 3.13
f1 (ppm)

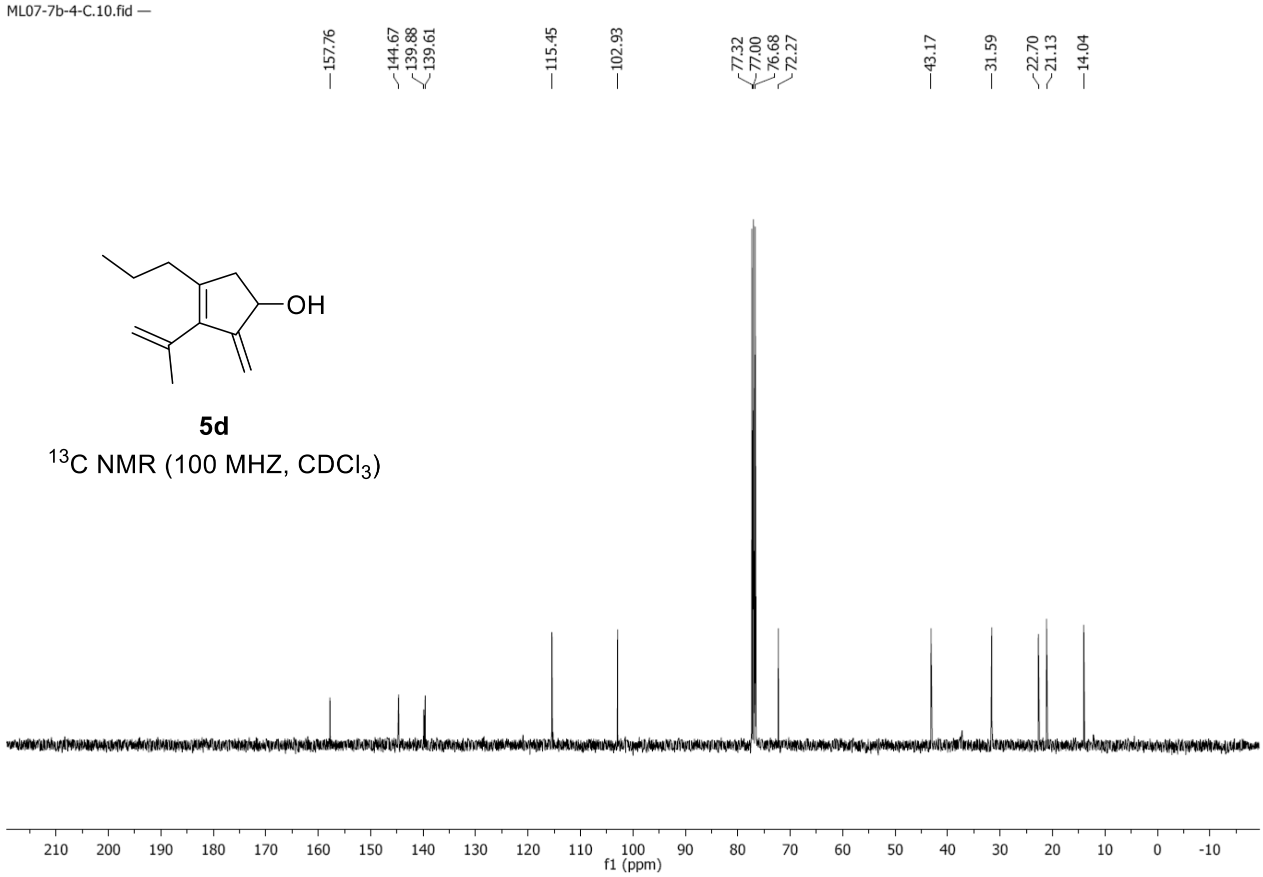
ML07-7b-4-C.10.fid
157.76 144.67 139.88 139.61 115.45 102.93 77.32 77.00 76.68 72.27 43.17 31.59 22.70 21.13 14.04
OH
5d
¹³C NMR (100 MHZ, CDCl₃)
f1 (ppm)

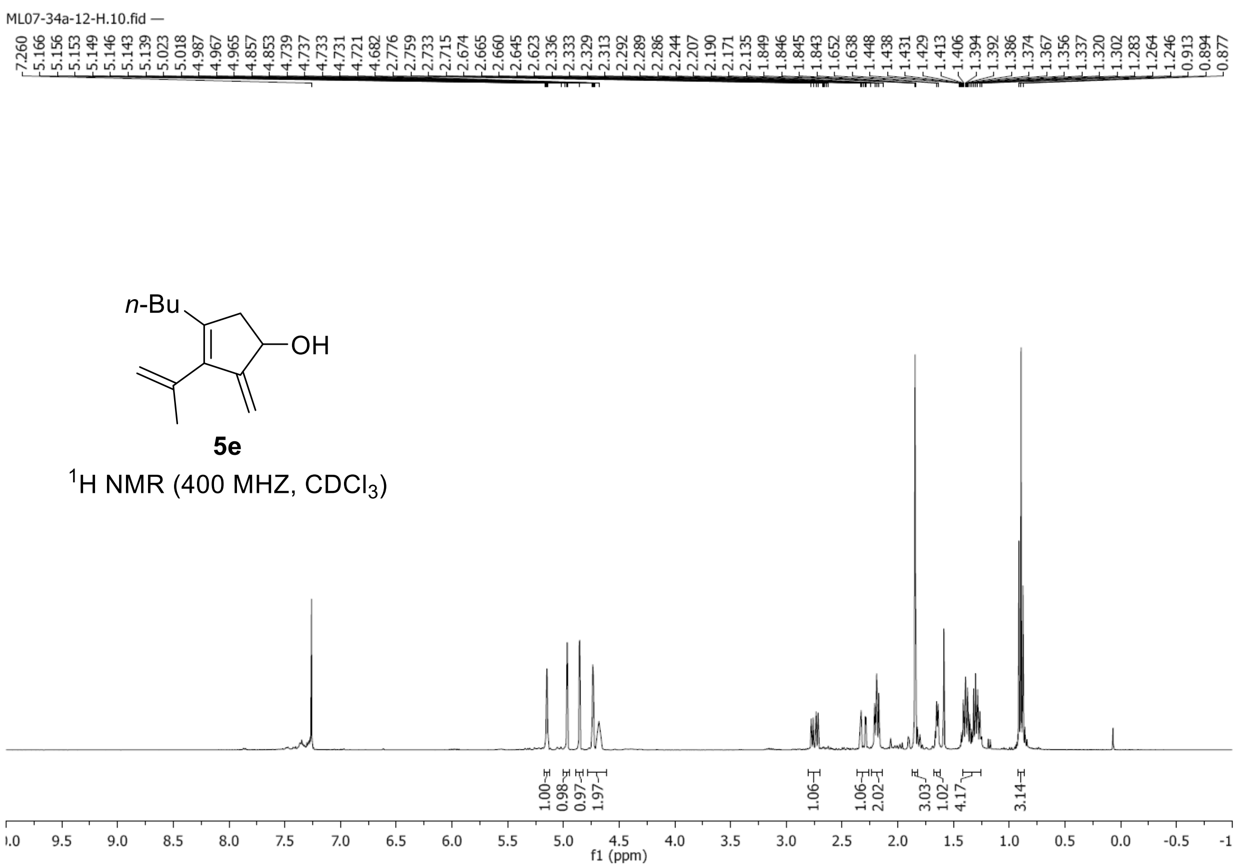
ML07-34a-12-H.10.fid
n-Bu
OH
5e
1H NMR (400 MHZ, CDCl3)
f1 (ppm)

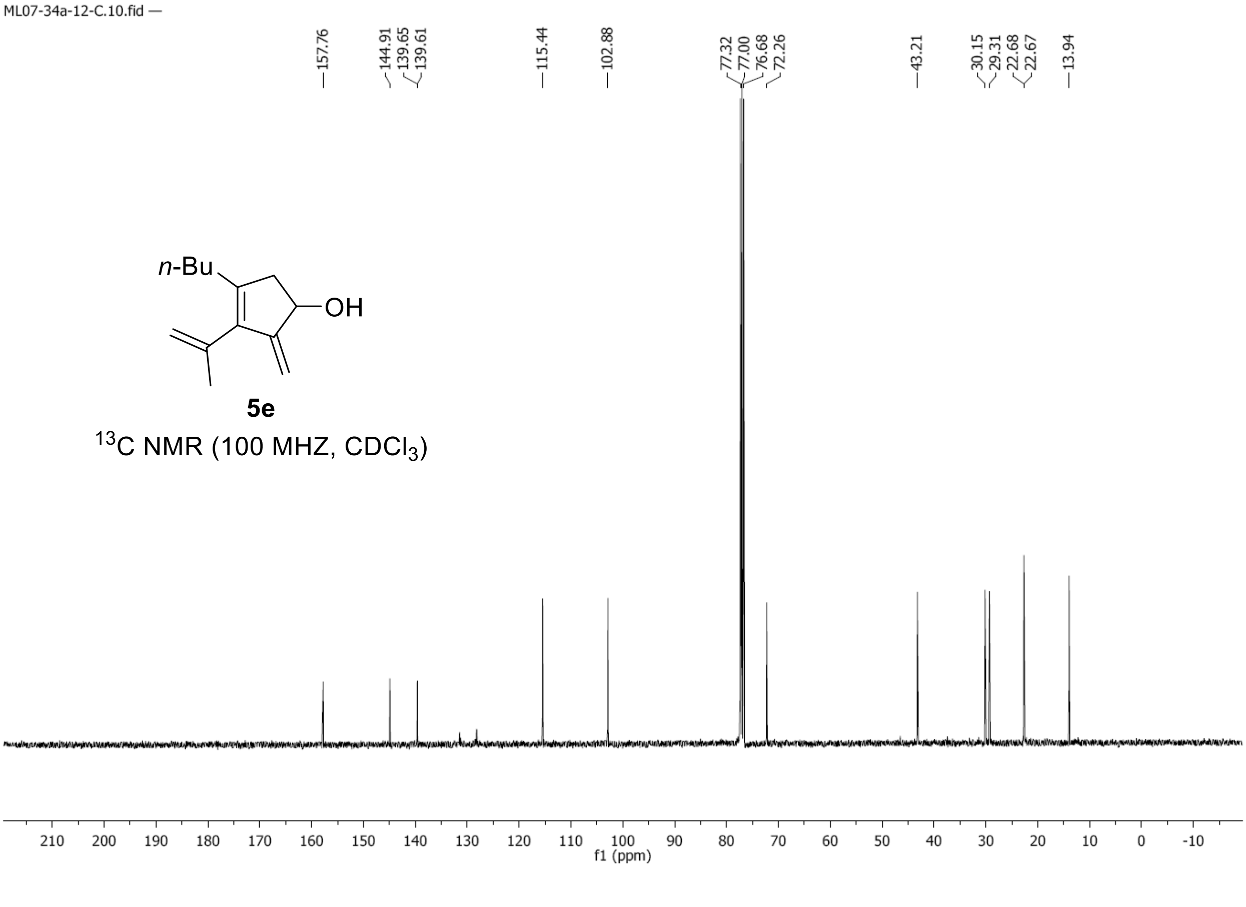
ML07-34a-12-C.10.fid
157.76
144.91
139.65
139.61
115.44
102.88
77.32
77.00
76.68
72.26
43.21
30.15
29.31
22.68
22.67
13.94
n-Bu
OH
5e
13C NMR (100 MHZ, CDCl3)
f1 (ppm)

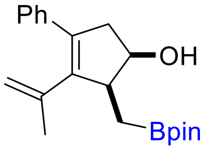

6

^{1}H NMR (400 MHZ, $CDCl_3$)

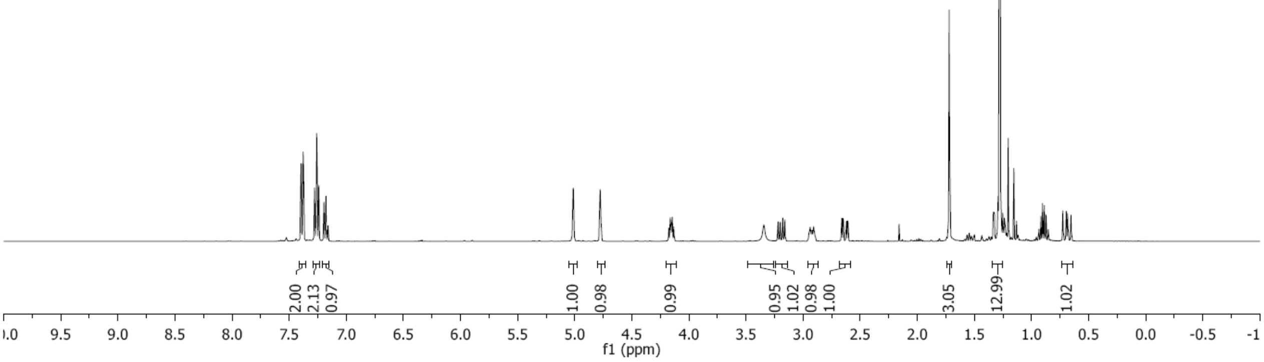

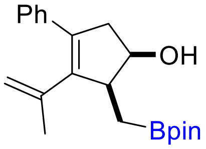

6

^{13}C NMR (100 MHZ, $CDCl_3$)

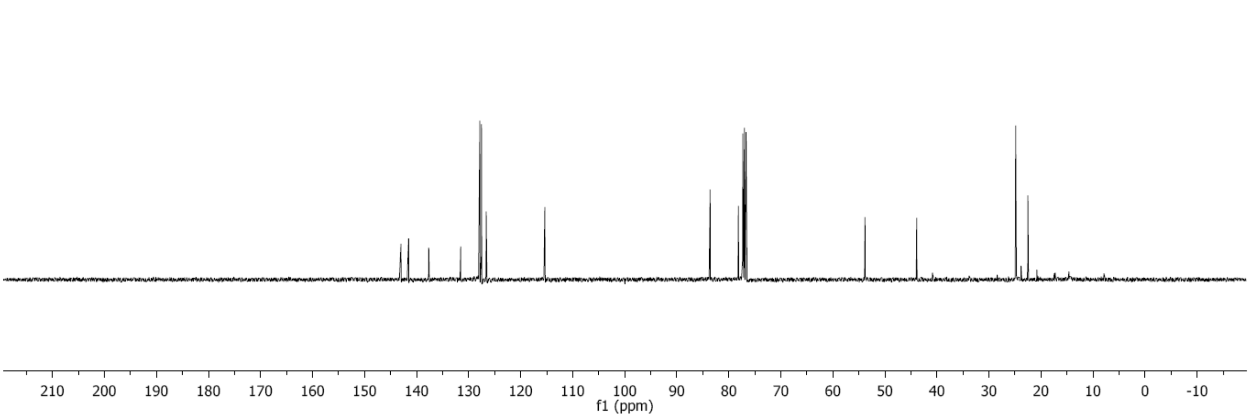

Supplement: Supplementary file 1 — Supplementary [file ANIE-60-670-s001.pdf]
